# One-pot syntheses of blue-luminescent 4-aryl-1*H*-benzo[*f*]isoindole-1,3(2*H*)-diones by T3P^®^ activation of 3-arylpropiolic acids

**DOI:** 10.3762/bjoc.13.231

**Published:** 2017-11-03

**Authors:** Melanie Denißen, Alexander Kraus, Guido J Reiss, Thomas J J Müller

**Affiliations:** 1 Institut für Organische Chemie und Makromolekulare Chemie, Heinrich-Heine-Universität Düsseldorf, Universitätsstraße 1, D-40225 Düsseldorf, Germany; 2 Institut für Anorganische Chemie und Strukturchemie, Heinrich-Heine-Universität Düsseldorf, Universitätsstraße 1, D-40225 Düsseldorf, Germany

**Keywords:** absorption, cycloaddition, diversity-oriented synthesis, fluorescence, heterocycles, imidation

## Abstract

In situ activation of 3-arylpropiolic acids with T3P^®^ (*n*-propylphosphonic acid anhydride) initiates a domino reaction furnishing 4-arylnaphtho[2,3-*c*]furan-1,3-diones in excellent yields. Upon employing these anhydrides as reactive intermediates blue-luminescent 4-aryl-1*H*-benzo[*f*]isoindole-1,3(2*H*)-diones are formed by consecutive pseudo three-component syntheses in a one-pot fashion. The Stokes shifts correlate excellently with the Hammett–Taft σ_R_ parameter indicating an extended degree of resonance stabilization in the vibrationally relaxed excited singlet state.

## Introduction

Luminophores as functional π-electron systems [1] are crucial in modern illumination technologies, such as organic light-emitting diodes (OLEDs) [2–5]. As a consequence efficient and efficacious syntheses of fluorescent molecules can be most advantageously addressed by diversity-oriented syntheses [6–8], namely by multicomponent [9–21] and domino reactions [22–24] where fundamental organic reactions are combined in one-pot sequences [25–29]. Multicomponent reactions (MCR) take advantage of creating and transforming reactive functionalities in the same reaction vessel without intermediary work-up [30]. Syntheses of functional chromophores by MCR [31–32] have indeed become a powerful tool in synthetic chemistry for convergently approaching substance libraries of luminescent molecules. In particular, blue-emissive heterocyclic chromophores, intensively requested in illumination research, are equally accessible by MCR strategies [33].

Cyclic imides are often prepared by condensation of acid anhydrides and amines [34]. Their applications are widespread, ranging from pharmaceutically active compounds [35] to agrochemicals [36] and fluorophores [37]. The characteristic photophysical properties of 1,8- and 2,3-naphthalene imides render the substance class particularly attractive for the development of novel sensors and fluorescent dyes [38], for instance 6-chloro-2,3-naphthaleneimide derivatives were successfully used for labeling amino acids, and for studying peptide protein interactions [39]. Even the superficial attachment to a binding domain can be detected and monitored by absorption and emission spectroscopy. Furthermore, the investigation of a substance library of various 2,3- and 1,8-naphthalene imides has shown that the electronic nature of the ground and the excited state is decisively influenced by variation of the substitution pattern of the naphthalene scaffold. Even the smallest polarity change in the solvent system effects their absorption and emission behavior [34,40]. Very recent investigations on naphthaleneimide derivatives revealed enormous phosphorescence lifetimes that are particularly interesting for imaging, sensing and display applications [41].

4-Phenylnaphtho[2,3-*c*]furan-1,3-diones can well serve as reactive intermediates in multicomponent reactions, e.g., for synthesizing the corresponding imides. A particularly intriguing access to 4-phenylnaphtho[2,3-*c*]furan-1,3-diones is the intramolecular [4 + 2]-cycloaddition of phenylpropiolic acid anhydrides. Usually, this synthesis requires harsh reaction conditions starting from phenylpropiolic acids [42–43]. However, employing bis(2-oxooxazolidin-3-yl)phosphinic chloride as an activating agent represents a suitable access [44]. Alternative approaches include the use of phenylpropiolic acid chloride and phenylpropiolic acid as starting materials [45], and as well oxidative arene–alkyne cyclization with dichloro-5,6-dicyano-benzoquinone (DDQ) [46].

Based upon our experience in using propylphosphonic acid anhydride (T3P^®^) [47] as a condensation agent for in situ activation of benzyl alcohols in the synthesis of *N*-benzylphenothiazine derivatives [48], we reasoned that T3P^®^ might be equally well suited for furnishing 4-phenylnaphtho[2,3-*c*]furan-1,3-diones, and thereby opening a straightforward entry to 4-aryl-1*H*-benzo[*f*]isoindole-1,3(2*H*)-diones in a diversity-oriented one-pot process. Here, we report the development of the one-pot synthesis of these title compounds by a consecutive pseudo three-component approach and the investigation of the luminescence behavior by absorption and emission spectroscopy.

## Results and Discussion

### Synthesis and structure of 4-arylnaphtho[2,3-*c*]furan-1,3-diones and 4-aryl-1*H*-benzo[*f*]isoindole-1,3(2*H*)-diones

**Scheme 1:**
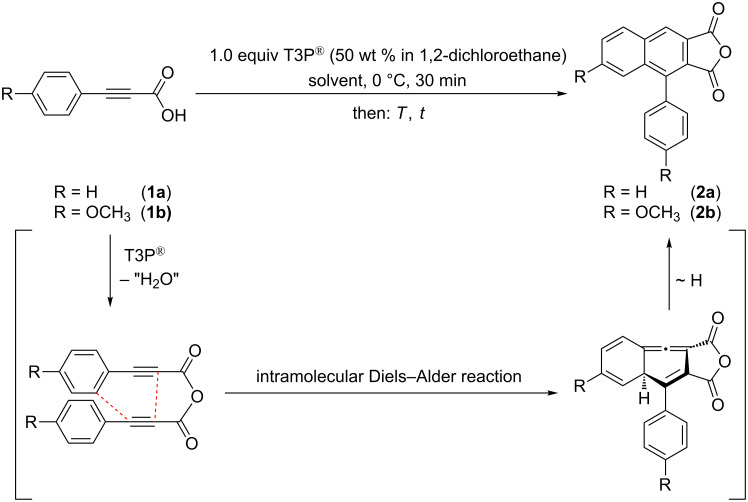
Mechanistic rationale and optimization of the domino synthesis of 4-arylnaphtho[2,3-*c*]furan-1,3-diones **2**.

Starting from phenylpropiolic acids **1** we first set out to optimize the reaction conditions for the domino synthesis of the 4-arylnaphtho[2,3-*c*]furan-1,3-diones **2a** and **2b** with T3P^®^ as an activating agent, varying temperature and reaction time (Scheme 1, Table 1).

**Table 1:** Optimization of the reaction conditions for the synthesis of 4-arylnaphtho[2,3-*c*]furan-1,3-diones **2**.

entry	arylpropiolic acid **1**	solvent	*T* [°C]	*t* [h]	4-arylnaphtho[2,3-*c*]furan-1,3-dione **2** (isolated yield) [%]

1	R = H **1a**	CH_2_Cl_2_	20	4	**2a** (81)
2	R = H **1a**	CH_2_Cl_2_	20	20	**2a** (81)
3	R = H **1a**	CH_2_Cl_2_	40	20	**2a** (21)
4^a^	R = H **1a**	CH_2_Cl_2_	20	20	**2a** (–)
5	R = OCH_3 _**1b**	CH_2_Cl_2_	20	20	**2b** (3)
6	R = OCH_3 _**1b**	THF	20	20	**2b** (2)

^a^Without addition of T3P^®^.

T3P^®^, employed as a 50 wt % solution in 1,2-dichloroethane (DCE), is added to a solution of phenylpropiolic acid (**1a**) in dichloromethane at 0 °C over the course of 30 min. After warming the reaction mixture to room temperature, stirring for 4 h and aqueous work-up the desired product **2a** can be obtained in good yield (Table 1, entry 1). A prolonged reaction time indicates that product **2a** is stable under the reaction conditions (Table 1, entry 2). However, a temperature increase to 40 °C already causes decomposition as indicated by a diminished isolated yield (Table 1, entry 3). The addition of T3P^®^ as an activation agent is a prerequisite for achieving the transformation (Table 1, entry 4).

**Scheme 2:**
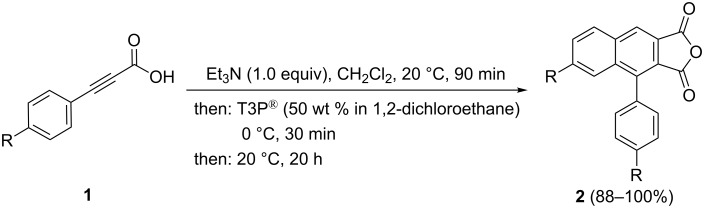
Domino synthesis of 4-arylnaphtho[2,3-*c*]furan-1,3-diones **2** via in situ activation of arylpropiolic acids **1**.

With these conditions in hand, 3-(4-methoxyphenyl)propiolic acid (**1b**) was also chosen as a model substrate (Table 1, entries 5 and 6). The optimized reaction conditions for the synthesis of 4-phenylnaphtho[2,3-*c*]furan-1,3-dione (**2a**) cannot directly be transferred to give the desired product **2b**, which was only obtained in traces. This is probably caused by the poor solubility of starting material **1b**. This problem can be circumvented by the addition of triethylamine as a deprotonating solubilizer [44]. Under these modified conditions four substituted 4-arylnaphtho[2,3-*c*]furan-1,3-dione derivatives **2** can be synthesized in very good to quantitative yields (Scheme 2, Table 2).

**Table 2:** Synthesized 4-arylnaphtho[2,3-*c*]furan-1,3-diones **2**.

entry	arylpropiolic acid **1**	4-arylnaphtho[2,3-*c*]furan-1,3-dione **2** (isolated yield [%])

1	R = H (**1a**)	**2a** (100)
2	R = OCH_3_ (**1b**)	**2b** (94)
3	R = CH_3_ (**1c**)	**2c** (91)
4	R = Cl (**1d**)	**2d** (88)

Interestingly, in this domino synthesis of 4-phenylnaphtho[2,3-*c*]furan-1,3-diones electron-rich and electro-neutral substrates are equally well tolerated (Table 2, entries 1–3), while the electron-poor derivative **1d** results in a slightly decreased yield of 88% due to the increased formation of side products (Table 2, entry 4). Expectedly, the products **2** are not stable under acidic aqueous conditions (e.g., hydrochloric acid, silica gel and saturated aqueous solution of ammonium chloride) [49]. After 48 h in the presence of deuterated hydrochloric acid (36% in D_2_O), complete conversion of compound **2a** to the free deuterated dicarboxylic acid was observed (20% conversion after 60 min) by NMR spectroscopy. For verification of the structure by NMR and IR spectroscopy, the hydrolysis was performed on a preparative scale with ammonium chloride in a mixture of water and acetone. After complete removal of water, the IR spectrum shows the characteristic OH-stretching vibrations of the free carboxylic acid (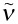 2901–3082 cm^−1^). The signals at δ 12.70 and 12.98 in the ^1^H NMR spectrum can be assigned to the two carboxylic acid functionalities (see Supporting Information File 1). The extraction of the crude anhydrides with a saturated aqueous solution of sodium bicarbonate can be achieved uneventfully, leaving the anhydride unimpaired.

**Scheme 3:**
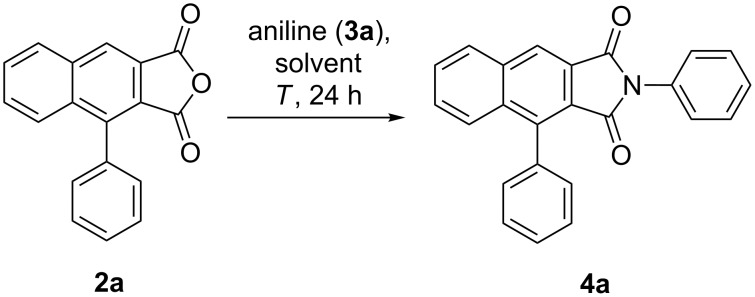
Optimization of the synthesis of 2,4-diphenyl-1*H*-benzo[*f*]isoindole-1,3(2*H*)-dione (**4a**) by imidation of 4-phenylnaphtho[2,3-*c*]furan-1,3-dione (**2a**) with aniline (**3a**).

For employing 4-phenylnaphtho[2,3-*c*]furan-1,3-diones **2** as reactive intermediates for the en route conversion with primary amines **3** into 4-aryl-1*H*-benzo[*f*]isoindole-1,3(2*H*)-diones **4** the reaction conditions were optimized with 4-phenylnaphtho[2,3-*c*]furan-1,3-dione (**2a**) and aniline (**3a**) as model substrates (Scheme 3, Table 3).

**Table 3:** Optimization of the reaction conditions for the synthesis of 2,4-diphenyl-1*H*-benzo[*f*]isoindole-1,3(2*H*)-dione (**4a**).

entry	equivalents of aniline (**3a**)	solvent *c*(**3a**) = 0.125 M	*T* [°C]	2,4-diphenyl-1*H*-benzo[*f*]isoindole-1,3(2*H*)-dione (**4a**) (isolated yield [%])

1	1.0	CH_2_Cl_2_	50	–
2	1.0	CH_2_Cl_2_/DMF 1:1 (v/v)	80	15
3	1.0	CH_2_Cl_2_/DMF 1:1 (v/v)	115	47
4	1.0	CH_2_Cl_2_/DMF 1:1 (v/v)	160	33
5	2.0	CH_2_Cl_2_/DMF 1:1 (v/v)	160	71
6	2.0	CH_2_Cl_2_/DMF 1:1 (v/v)	115	80

In dichloromethane at only slightly elevated temperatures imide **4a** is not formed (Table 3, entry 1). Upon addition of *N,N-*dimethylformamide as a cosolvent at 80 °C the desired product **4a** can be isolated in 15% yield (Table 3, entry 2). The yield of **4a** can be increased to 47% upon raising the reaction temperature to 115 °C (Table 3, entry 3), however, higher temperatures, such as 160 °C, cause a significant drop in yield. Finally, at 115 °C with two equivalents of aniline the highest yield can be achieved (Table 3, entry 6).

**Scheme 4:**
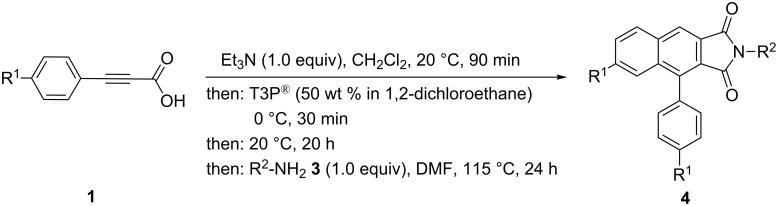
Pseudo three-component synthesis of 4-aryl-1*H*-benzo[*f*]isoindole-1,3(2*H*)-diones **4**.

These imidation conditions are well-suited for concatenating the arylpropiolic anhydride formation, intramolecular cycloaddition and imidation in a one-pot fashion in the sense of a consecutive pseudo three-component synthesis of 4-aryl-1*H*-benzo[*f*]isoindole-1,3(2*H*)-diones **4**, which was first probed with an electroneutral (**1a**), an electron-rich (**1b**) and electron-poor (**1c**) propiolic acid substrate (Scheme 4, Table 4). Although the electroneutral and electron-rich substrates give good to excellent yields in the sequence (Table 4, entries 1–3), even higher (Table 4, entry 1) than for a stepwise synthesis furnishing an overall yield of **4a** of 80%, the cyano-substituted substrate **1c** furnishes a significantly lower yield of 27% (Table 4, entry 4), originating from the lower reactivity in the anhydride formation.

**Table 4:** Pseudo three-component synthesis of 1*H*-benzo[*f*]isoindole-1,3(2*H*)-diones **4**.

entry	arylpropiolic acid **1**	amine **3**	4-aryl-1*H*-benzo[*f*]isoindole-1,3(2*H*)-dione **4** (isolated yield [%])

1	R^1^ = H (**1a**)	R^2^ = C_6_H_5_ (**3a**)	**4a** (95)
2	R^1^ = OCH_3_ (**1b**)	**3a**	**4b** (79)
3^a^	**1a**	R^2^ = CH_2_(10-methyl-10*H*- phenothiazine-3-yl)∙HCl (**3b**)	**4c** (85)
4	R^1^ = CN (**1c**)	**3a**	**4d** (27)

^a^Compound **3b** was employed as the corresponding hydrochloride with 202 mg (2.00 mmol) Et_3_N in CH_2_Cl_2_ (1.0 mL) at rt for 1 h.

**Scheme 5:**
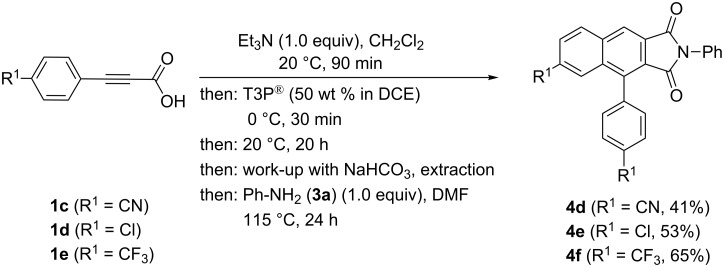
Modified sequence for the synthesis of acceptor-substituted 4-aryl-1*H*-benzo[*f*]isoindole-1,3(2*H*)-diones **4**.

Therefore, for accessing acceptor-substituted derivatives of **4**, an intermediate extraction with bicarbonate after the anhydride formation–cycloaddition was attempted (Scheme 5). With this variation acceptor-substituted arylpropiolic acids **1c–e** can be transformed to the corresponding 1*H*-benzo[*f*]isoindole-1,3(2*H*)-diones **4d–f** in moderate yields.

Upon lowering the amount of T3P^®^ to 0.5 equiv, electron-neutral and electron-donating phenylpropiolic acids **1** can be employed with comparable efficiency applying various amines **3** and giving rise to a series of 4-aryl-1*H*-benzo[*f*]isoindole-1,3(2*H*)-diones **4** in moderate to good yield (Table 5). Variation of the amine nucleophile **3** allows for the introduction of functional groups for consecutive transformations. For instance, *para*-substituted halogenated anilines **3** (Table 5, entries 3–5) and ethyl 4-aminobenzoate (Table 5, entry 6) furnish the desired products in 51–65% yield. Interestingly, also sterically hindered anilines, such as 2,6-dimethylaniline can be obtained in 56% yield (Table 5, entry 8). In addition, aliphatic amines (Table 5, entries 10–13) are readily tolerated in the established reaction sequence.

**Table 5:** Variation of the amine **3** in pseudo three-component syntheses of 1*H*-benzo[*f*]isoindole-1,3(2*H*)-diones **4**.

entry	arylpropiolic acid **1**	amine **3**	1*H*-benzo[*f*]isoindole-1,3(2*H*)-dione **4** (isolated yield [%])

1	**1a**	R^2^ = C_6_H_5_ (**3a**)	**4a** (92)
2	R^1^ = CH_3_ (**1f**)	**3a**	**4g** (48)
3	R^1^ = **1a**	R^2^ = *p*-FC_6_H_4_ (**3c**)	**4h** (55)
4	**1a**	R^2^ = *p*-ClC_6_H_4_ (**3d**)	**4i** (65)
5	**1a**	R^2^ = *p*-IC_6_H_4_ (**3e**)	**4j** (51)
6	**1a**	R^2^ = *p*-EtO_2_CC_6_H_4_ (**3f**)	**4k** (57)
7	**1a**	R^2^ = 3,5-Me_2_C_6_H_3_ (**3g**)	**4l** (69)
8	**1a**	R^2^ = 2,6-Me_2_C_6_H_3_ (**3h**)	**4m** (56)
9	**1a**	R^2^ = 3,5-(MeO)_2_C_6_H_3_ (**3i**)	**4n** (64)
10	**1a**	R^2^ = CH_2_Ph (**3j**)	**4o** (41)
11^a^	**1a**	R^2^ = CH_2_CCH (**3k**)	**4p** (26)
12	**1a**	R^2^ = *n*-hexyl (**3l**)	**4q** (53)
13	**1a**	R^2^ = *n*-butyl (**3m**)	**4r** (61)
14	**1b**	**3h**	**4s** (57)

**Figure 1:**
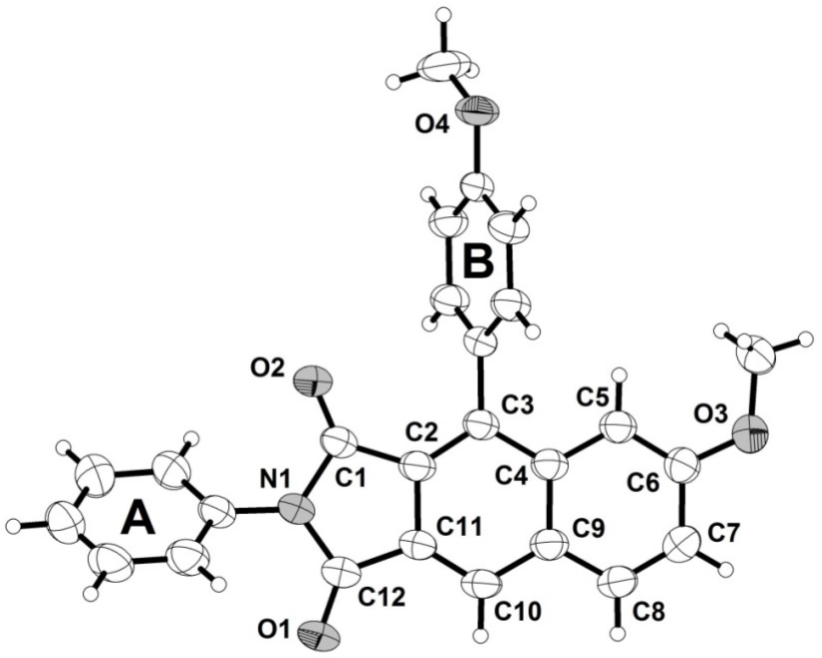
The ORTEP-style plot of crystal structure **4b** (ellipsoids are draw at the 40% probability level).

Besides comprehensive NMR spectroscopic and mass spectrometric characterization the structures of the title compounds **4** were additionally corroborated by a crystal structure determination of compound **4b** (Figure 1). The twist angle of the phenyl substituent (ring A) and the 1*H*-pyrrole-2,5-dionyl moiety is 51.37(7)°, whereas the *p*-anisyl substituent (ring B) is considerably twisted against the adjacent six-membered ring by 70.95(7)° (Figure 1) [50].

**Scheme 6:**
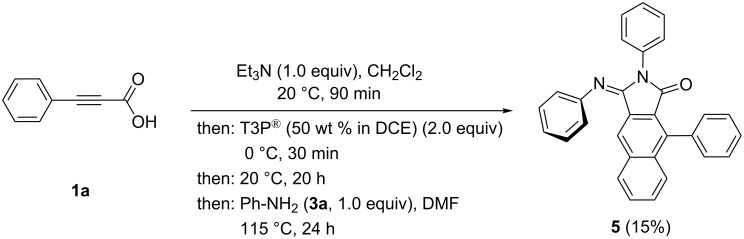
Pseudo four-component synthesis of (*E*)-2,9-diphenyl-3-(phenylimino)-2,3-dihydro-1*H*-benzo[*f*]isoindol-1-one (**5**).

Upon slightly varying the reaction conditions the potential of the sequence can be extended. An increase of the T3P^®^ concentration to 2.0 equiv leads to the regioselective formation of (*E*)-2,9-diphenyl-3-(phenylimino)-2,3-dihydro-1*H*-benzo[*f*]isoindol-1-one (**5**) in 15% yield in the sense of a pseudo four-component reaction (Scheme 6).

**Scheme 7:**
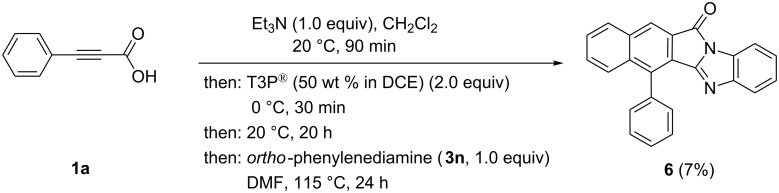
Synthesis of 6-phenyl-12*H*-benzo[*f*]benzo[4,5]imidazo[2,1-*a*]isoindol-12-one (**6**).

The increased amount of the condensation agent T3P^®^ obviously enables further activation of the initially formed 1*H*-benzo[*f*]isoindole-1,3(2*H*)-dione by electrophilic attack on the sterically easier accessible carbonyl group. As a consequence the imine condensation proceeds also with thermodynamic control giving exclusively the formation of the *E*-configured product **5**. Interestingly the corresponding reaction with *ortho*-phenylenediamine (**3n**) gives rise to the regioselective formation of the pentacyclic condensation product **6**, where the intramolecular imine formation formally occurred at the sterically more biased carbonyl group (Scheme 7).

**Figure 2:**
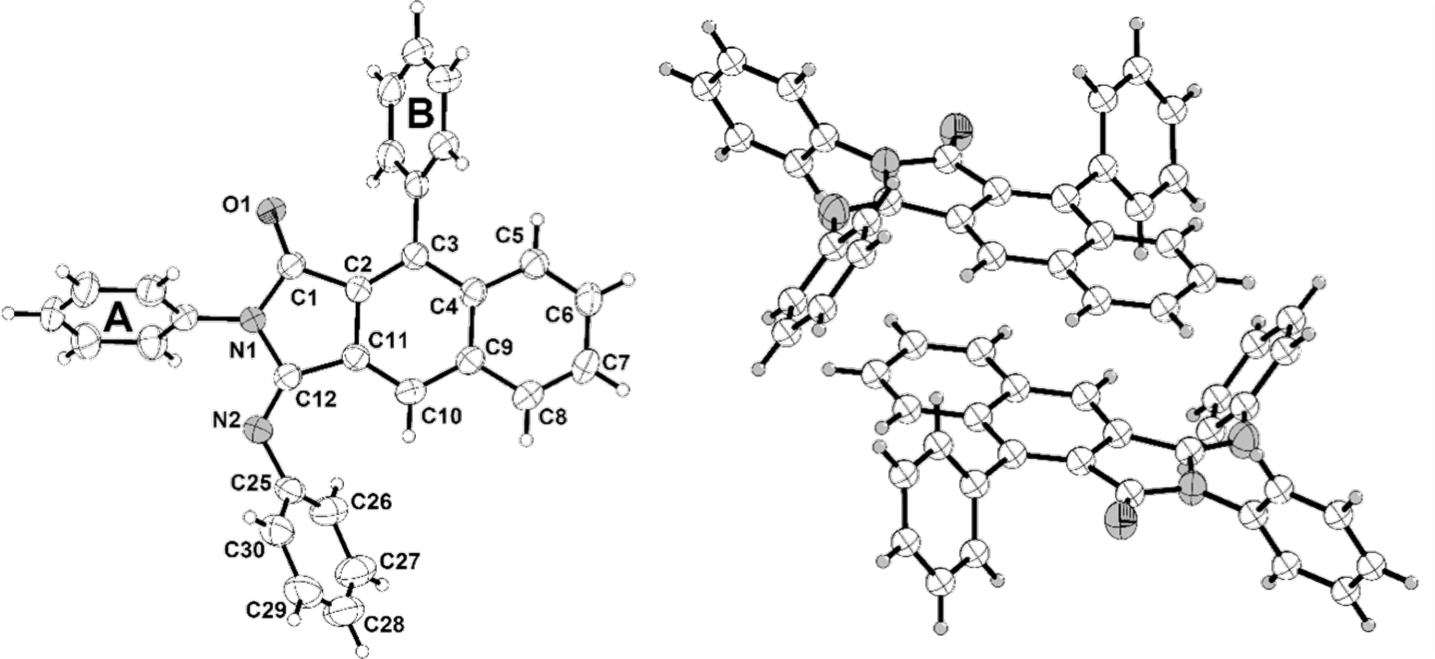
The ORTEP-type plot of the crystal structure **5** (left) and a centrosymmetric dimer formation by π–π interactions (right) (ellipsoids are drawn at the 40% probability level).

**Figure 3:**
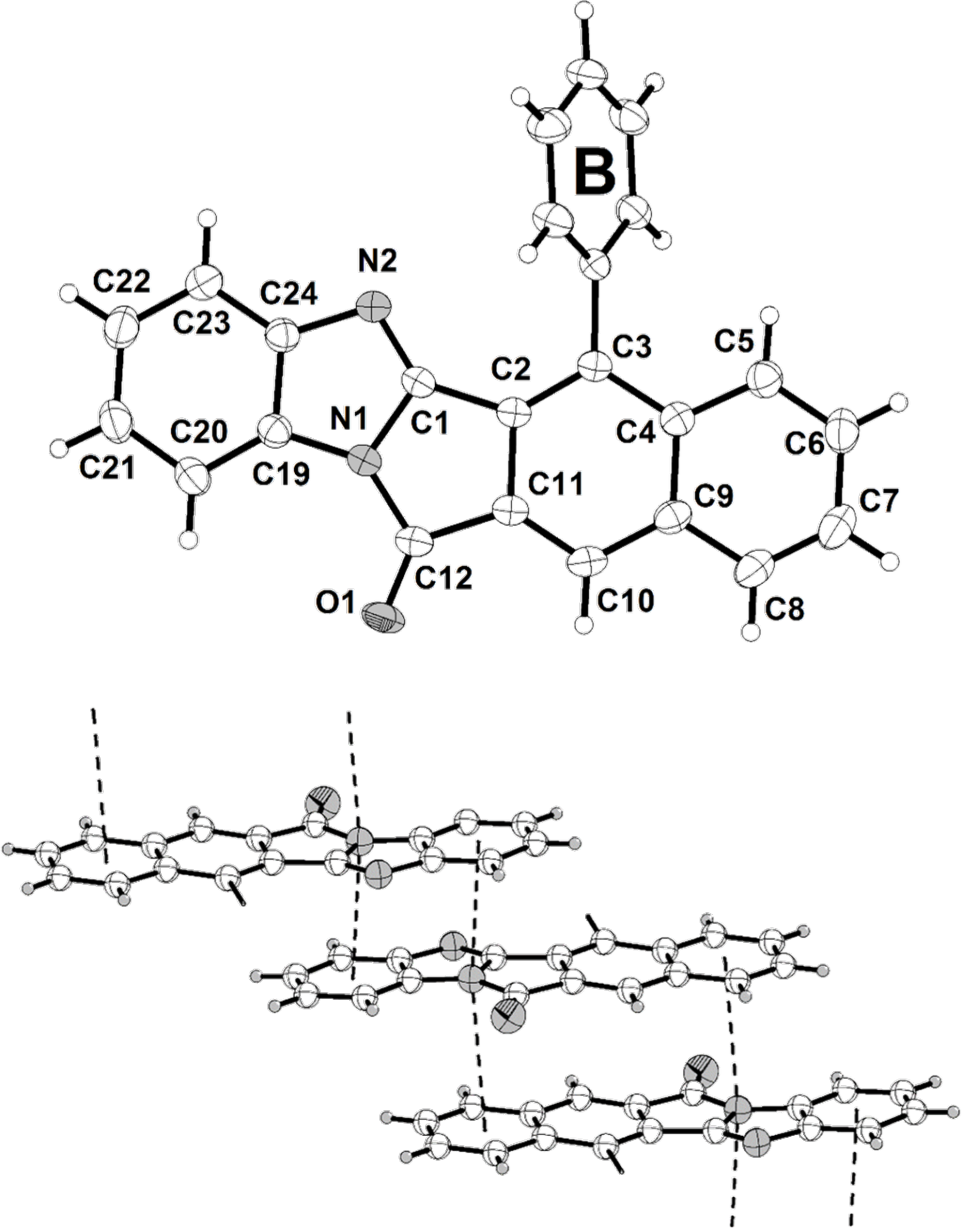
The ORTEP-type plot of the asymmetric unit of the crystal structure **6** (top) and π-stacking interactions (bottom) (ellipsoids are drawn at the 40% probability level).

In addition to NMR spectroscopic and mass spectrometric characterization the crystal structures of the imine condensation products **5** and **6** were determined (Figure 2 and Figure 3) [50]. In similarity to **4b** the twist angles of the phenyl substituents (rings A and B) are 61.1(1)° and 66.8(1)°, respectively (Figure 2, left part). Interestingly, the crystal structure of **5** shows centrosymmetric dimers, formed by a close π–π interaction of the planar naphthyl moieties (Figure 2, right part). The intermolecular distance of the naphthalene moieties of the two molecules accounts to 3.435(7) Å.

This intermolecular plane distance of the two adjacent naphthyl moieties (C2–C11) must be understood as a π–π interaction of two fused aromatic systems [51]. The twist angle of the attached phenyl substituent (ring B) of the asymmetric unit of the crystal structure **6** were determined as 62.28(5)° (Figure 3). The significant difference of the twist angles in **4b**, **5**, and **6** could be a consequence of packing in the individual crystal structure. The crystal structure of the pentacyclic molecules **6** shows stacks with an antiparallel arrangement of the monomers. The found interplanar distance is 3.437(4) Å. Consequently, π–π interactions must be considered for this structure [51–52]. Bond lengths and angles of the reported crystal structures **4b**, **5**, and **6** are in the expected range. The tricyclic 1*H*-benzo[*f*]isoindole-1,3(2*H*)-dionyl moiety in **4b**, **5** and the corresponding 3-imino-1-oxo-2,3-dihydro-1*H*-benzo[*f*]isoindolyl moiety in **6** are absolutely planar.

### Photophysical properties

**Figure 4:**
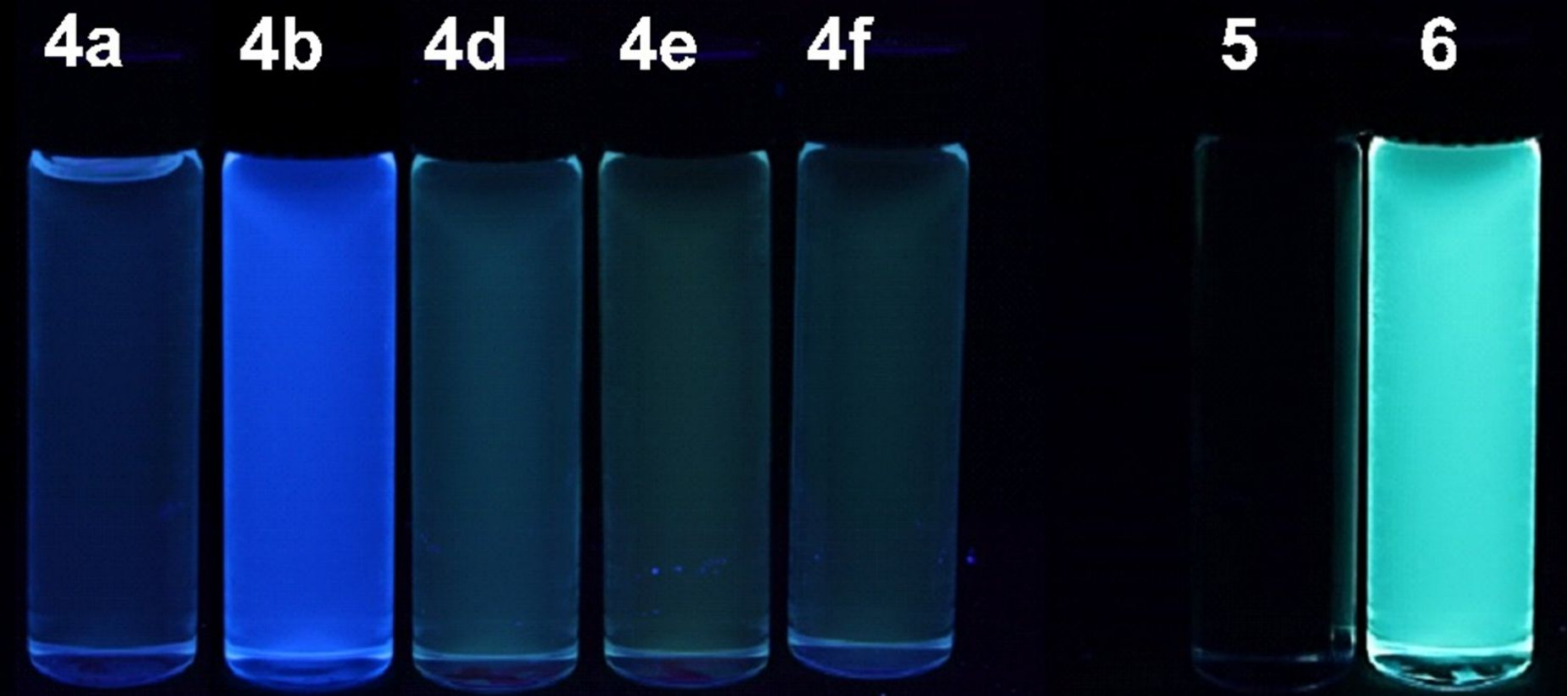
Emission properties of compounds **4a**,**b**,**d–f**, **5**, and **6** under handheld UV-lamp (λ_exc_ ≈ 350 nm).

The pseudo three-component synthesis of 1*H*-benzo[*f*]isoindole-1,3(2*H*)-diones **4** furnishes a substance library with electronically diverse substitution patterns and already upon eyesight several derivatives are intensively blue and greenish luminescent in solution at low concentration (Figure 4). Therefore, the 1*H*-benzo[*f*]isoindole-1,3(2*H*)-diones **4** and **5** and the pentacyclic compound **6** were investigated with absorption and static fluorescence spectroscopy (Table 6).

**Table 6:** Selected photophysical properties (absorption and emission maxima,^a,b^ fluorescence quantum yields (Φ_f_ [53]),^c^ and Stokes shifts Δ
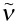
^d^) of compounds **4a–g**,**m**,**n**,**s**, **5**, and **6**.

entry	compound	λ_max,abs_ (ε) [nm] ([L∙(mol∙cm)^−1^])	λ_max,em_ [nm] (Φ_f_)	Stokes shift Δ 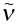 [cm^−1^]

1	**4a**	364.5 (3900), 264.0 (49500)	468.0 sh, 408.5 (0.01)	3000
2	**4b**	379.0 (4000), 273.5 (55300)	444.0 (0.14)	3900
3	**4c**	363.0 (5080), 300.5 (13510), 262.5 (77840)	525.0 sh, 450.0	5300
4	**4d**	365.0 (3600), 266.5 (57300)	512.5, 401.0 sh	2500
5	**4e**	366.0 (3000), 268.5 (52600)	499.5, 417.0 sh	3300
6	**4f**	359.0 (3100), 343.0 (2600), 258.5 (50700)	500.0, 397.0 sh	2700
7	**4g**	369.5 (4200), 270.5 (53000)	415.0	3000
8	**4m**	364.0 (5400), 263.5 (58000)	391.0	2000
9	**4n**	364.0 (5600), 264.5 (67000)	520.0	8200
10	**4s**	371.5 (4700), 271.5 (56300)	437.0 (0.17)	4000
11	**5**	362.5 (5900), 350.0 (4800) 265.5 (52900), 261.0 (52500)	–	–
12	**6**	388.5 sh (21600), 360.0 (54900) 296.0 (53000), 255.5 (21500)	500.0 (0.10)^d^	5700

^a^Recorded in CH_2_Cl_2_ UVASOL^®^ at *T* = 293 K. ^b^Recorded in CH_2_Cl_2_ UVASOL^®^ at *T* = 293 K with λ_exc_ = 360.0/380.0 nm. ^c^Quantum yields Φ_f_ were determined with coumarine 1 in ethanol as a standard, Φ_f_ = 0.73, at *T* = 293 K with λ_exc_ = 360.0 nm. ^d^Δ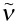 = 1/λ_max,abs_ – 1/λ_max,em_ [cm^−1^]. ^e^Quantum yields Φ_f_ were determined with coumarine 30 in acetonitrile as a standard, Φ_f_ = 0.67, at *T* = 293 K with λ_exc_ = 380.0 nm.

All 1-aryl-2,3-naphthaleneimides **4** possess two characteristic absorption maxima λ_max,abs_ between 258.5 and 273.5 nm with molar extinction coefficients ε of 55000 L∙mol^−1^∙cm^−1^ and between 359.0 and 379.0 nm with molar extinction coefficients ε of about 3500 L∙mol^−1^∙cm^−1^. While electron-withdrawing substituents R^1^ in tendency shift the absorption maxima slightly hypsochromically (Table 6, entries 1, and 3–6) the electron-donating methoxy group (Table 6, entry 2) clearly causes a red shift. The absorption spectra of the imine condensation products **5** and **6** essentially display a similar appearance; however, the pentacycle **6** possesses a considerably more intense longest wavelength absorption, which appears in the spectrum at 388.5 nm as a shoulder with a molar extinction coefficients ε of 21600 L∙mol^−1^∙cm^−1^ (Table 6, entry 12).

**Figure 5:**
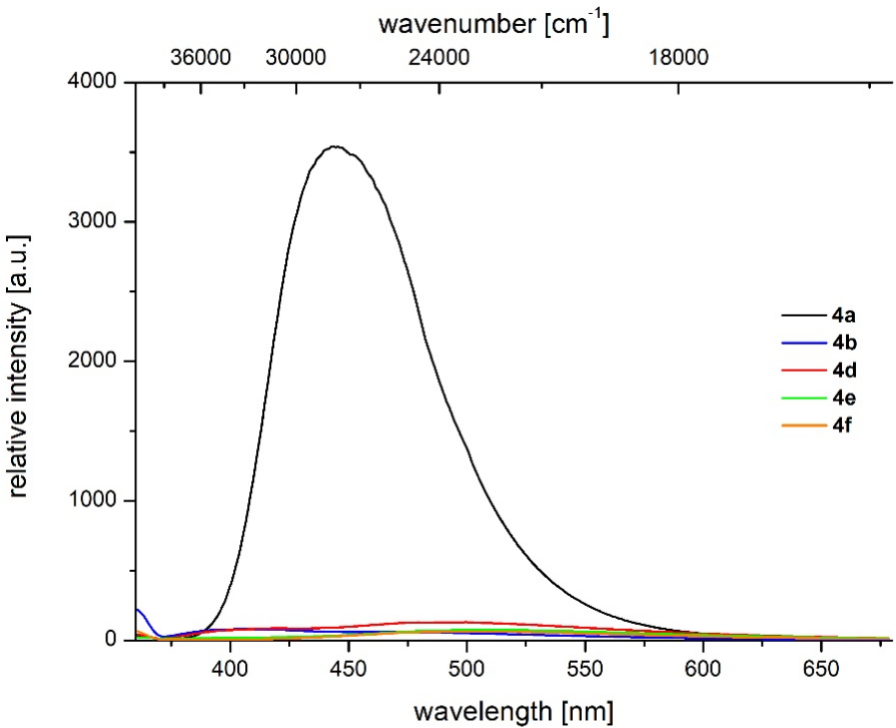
Relative emission intensities of compounds **4a**,**b**,**d–f** (recorded in CH_2_Cl_2_ UVASOL^®^ at *T* = 293 K; λ_exc_ = 350 nm.

**Figure 6:**
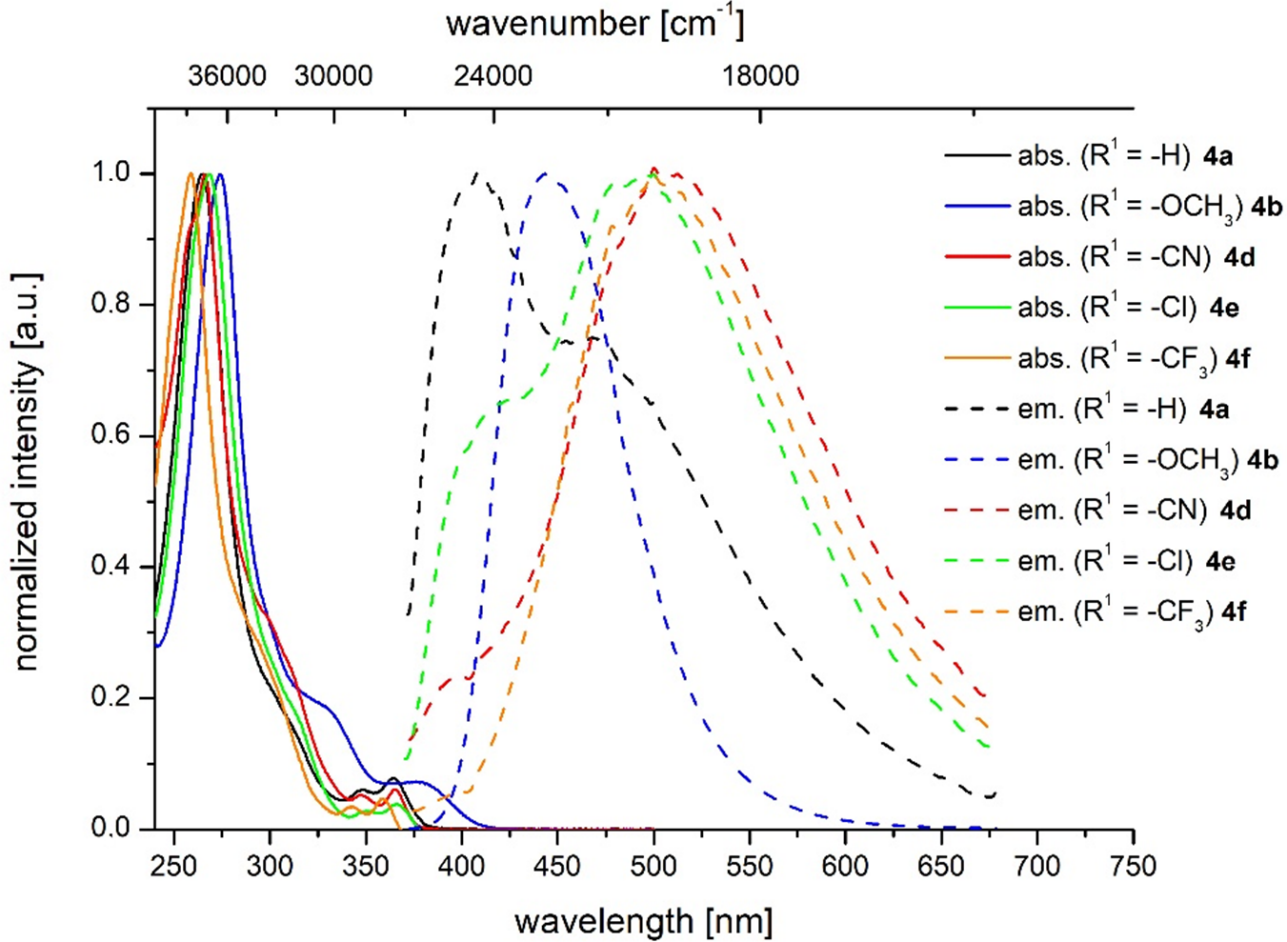
Absorption and emission properties of selected imides **4** measured in CH_2_Cl_2_ UVASOL^®^ at 293 K with λ_exc_ = 360 nm.

Although most of the 1*H*-benzo[*f*]isoindole-1,3(2*H*)-diones **4** fluorescence upon excitation with UV light, a closer inspection, by comparing the relative intensities of the emission maxima at identical concentrations, reveals that only the methoxy derivative **4b** (Table 6, entry 2) is substantially fluorescent (Figure 5). With this exception all qualitatively determined emission spectra reveal a broad unstructured maximum followed by a shoulder. This appearance might result from the free rotation of the *N*-phenyl substituents, furnishing emissive conformers that arise from a coplanar (λ_max,em_ at 468.0 nm as a shoulder for compound **4a**) and torsional orientation (λ_max,em_ at 408.5 nm as a maximum for compound **4a**) of the *N*-phenyl substituent in the corresponding excited Franck–Condon states [54]. Qualitatively, also a red shift of the emission maximum can be detected upon increasing the electron-withdrawing character of the substituent R^1^. In the consanguineous series of 1*H*-benzo[*f*]isoindole-1,3(2*H*)-diones **4a**,**b**,**d–f** the emission maxima λ_max,em_ are found in a range from 408.5 to 512.5 nm with Stokes shifts lying between 3900 and 2500 cm^−1^ (Table 6, entries 1, 2 and 4–6, Figure 6).

**Figure 7:**
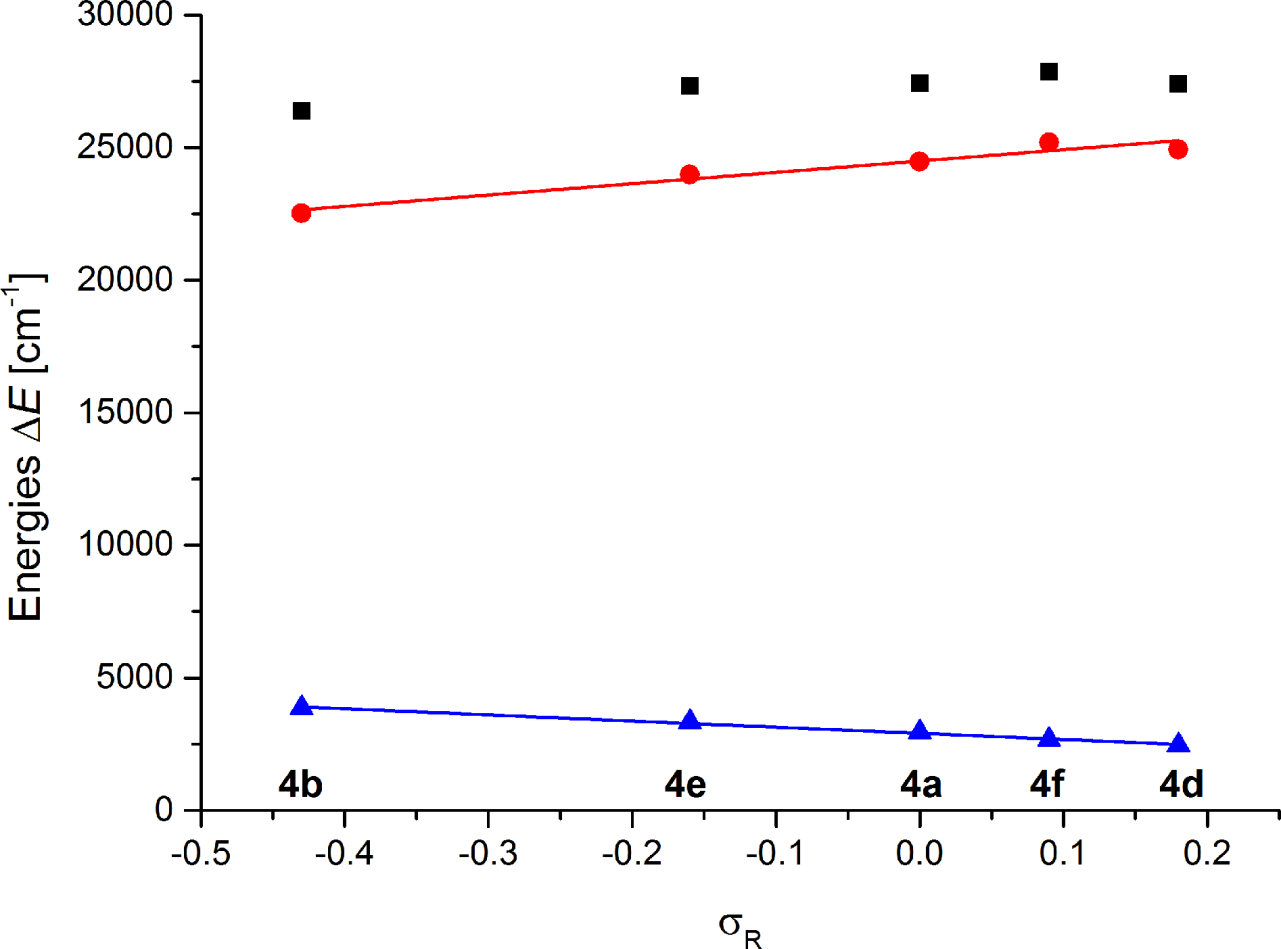
Hammett–Taft correlations of the emission maxima (red circles, l_max,em_ = 4274 · s_R_ + 24495 [cm^−1^], R^2^ = 0.925) and Stokes shifts (blue triangles, Δ = −2319 · s_R_ + 2909 [cm^−1^], R^2^ = 0.989) of compounds **4a**,**b**,**d–f** with s_R_ (black squares are the corresponding absorption maxima).

This electronic substituent effect of R^1^ on the Stokes shifts was further corroborated by linear structure–property relationships based upon Hammett–Taft correlations with the consanguineous series **4a,b,d–f**. Correlation studies of the longest wavelength absorption maxima λ_max,abs_, the shortest wavelength emission maxima λ_max,em_, and the Stokes shifts Δ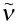 with the Hammett–Taft parameters σ_p_, σ_R,_ σ_p+_, and σ_p−_ [55] disclose an interesting insight on electronic substituent effects in the electronic ground and excited states (see Table S6 in Supporting Information File 1). Although the linear correlations of λ_max,abs_ with all σ parameters are relatively poor, the correlations of λ_max,em_ with σ_R_ and σ_p+_ indicate a strong influence of resonance stabilization in the vibrationally relaxed excited state. This is even more the case in the nearly perfect linear correlation of the Stokes shift with σ_R_ (r^2^ = 0.989) and can be interpreted as a significant structural change upon photonic excitation and excited state relaxation resulting from a considerable charge transfer character, as supported by the influence of the remote polar substitution (Figure 7).

Interestingly, compound **4a**, which has a fluorescence quantum yield Φ_f_ of less than 0.01 in dichloromethane solution, experiences an over eightfold increase to 0.08 in the solid state emission as determined from the powder by an integrating sphere. The emission maximum appears at 468.5 nm, i.e., at the same longest wavelength band as in solution. Therefore, 1*H*-benzo[*f*]isoindole-1,3(2*H*)-diones **4** can be considered as AIE (aggregation induced emission) chromophores [56–58].

**Figure 8:**
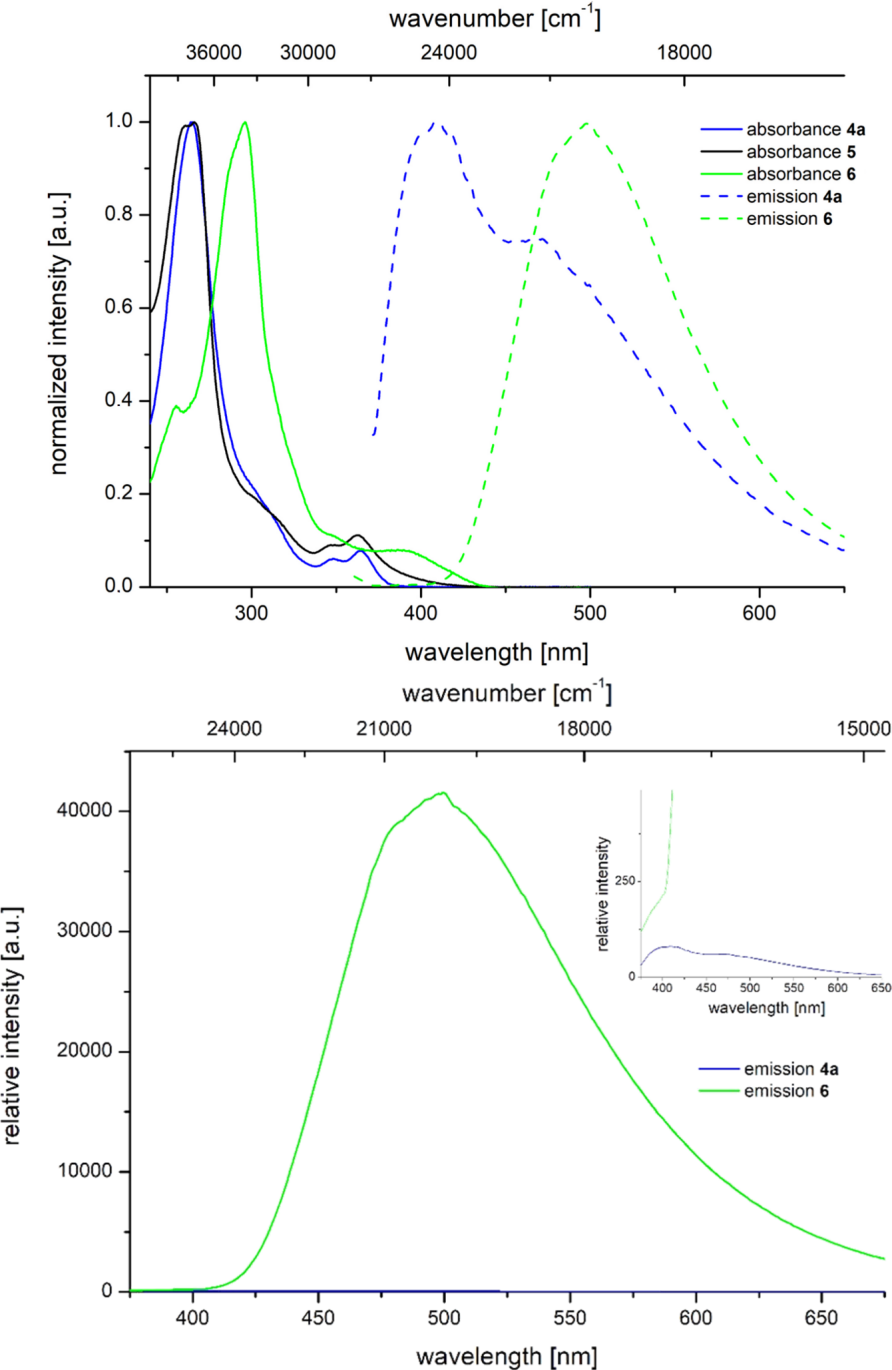
Relative emission intensities of the 1-phenyl-2,3-naphthaleneimide **4a** (blue) and the pentacyclus **6** (green, normalized on the absorptivity at the excitation wavelength λ_exc_ = 350 nm).

In comparison to the 1-phenyl-[2,3-*c*]-naphthaleneimide **5**, which is only weakly luminescent, the pentacycle **6** displays a relative enhancement of the greenish emission at 500 nm by a factor of 340 (relative to compound **4a**, Figure 8) and can be quantified with a fluorescence quantum yield of 0.10 (Table 6, entry 12).

## Conclusion

3-Arylpropiolic acids can be readily activated with T3P^®^ (*n*-propylphosphonic acid anhydride) to initiate a domino reaction furnishing 4-arylnaphtho[2,3-*c*]furan-1,3-diones in excellent yields. These anhydrides can be considered as reactive intermediates for a subsequent imidation with primary amines and, therefore, a one-pot reaction in the sense of a consecutive pseudo three-component process evolved. The resulting 4-aryl-1*H*-benzo[*f*]isoindole-1,3(2*H*)-diones are interestingly blue to greenish-blue emissive upon excitation of the longest wavelength absorption bands. The photophysical characterization by absorption and emission spectroscopy revealed that the Stokes shifts are excellently correlated with Hammett–Taft's σ_R_ parameters indicating an extended degree of resonance stabilization as a result of a charge transfer character in the vibrationally relaxed S_1_-state. The fluorescence can be redshifted by employing 1,2-phenylenediamine as a reaction partner in the terminal step of the sequence furnishing a rigidified planar pentacyclic condensation product. The interesting emission properties and the straightforward diversity-oriented synthetic approach are therefore well-suited for the design of covalently ligated, conjugated and non-conjugated bichromophores in a rapid fashion. Studies directed towards the one-pot synthesis of more complex polycyclic emitters are currently underway.

## Supporting Information

The Supporting Information contains all experimental procedures, spectroscopic and analytical data as well as copies of NMR spectra of compounds **2**, **4**, **5**, and **6**. X-ray structure analyses of compounds **4b**, **5**, and **6**, and Hammett–Taft correlations of compounds **4a**,**b**,**d–f** are also given.

File 1 Experimental part.
