# Supplementary material for: One-pot syntheses of blue-luminescent 4-aryl-1H-benzo[f]isoindole-1,3(2H)-diones by T3P® activation of 3-arylpropiolic acids
Source: Beilstein J Org Chem. 2017 Nov 3;13:2340–51. doi: 10.3762/bjoc.13.231 (PMC5687056; doi:10.3762/bjoc.13.231)
Supplement: File 1 — Experimental part. [file Beilstein_J_Org_Chem-13-2340-s001.pdf]

Supporting Information

for

One-pot syntheses of blue-luminescent 4-aryl-1*H*-benzo[*f*]isoindole-1,3(2*H*)-diones by T3P® activation of 3-arylpropiolic acids

Melanie Denißen[1], Alexander Kraus[1], Guido J. Reiss[2] and Thomas J. J. Müller*,[1]

Address: [1]Institut für Organische Chemie und Makromolekulare Chemie, Heinrich-Heine-Universität Düsseldorf, Universitätsstraße 1, D-40225 Düsseldorf, Germany and [2]Institut für Anorganische Chemie und Strukturchemie, Heinrich-Heine-Universität Düsseldorf, Universitätsstraße 1, D-40225 Düsseldorf, Germany

Email: Thomas J. J. Müller - ThomasJJ.Mueller@uni-duesseldorf.de

* Corresponding author

Experimental part

Contents

1. Experimental considerations S3
2. Domino synthesis and analytical data of 4-arylnaphtho[2,3-c]furan-1,3-diones 2 (GP1) S4
2.1. 4-Phenylnaphtho[2,3-*c*]furan-1,3-dione (**2a**) S5
2.2. 6-Methoxy-4-(4-methoxyphenyl)naphtho[2,3-*c*]furan-1,3-dione (**2b**) S5
2.3. 6-Methyl-4-(4-tolyl)naphtho[2,3-*c*]furan-1,3-dione (**2c**) S6
2.4. 6-Chloro-4-(4-chlorophenyl)naphtho[2,3-*c*]furan-1,3-dione (**2d**) S6
2.5. Hydrolysis of compound **2a** and analytical data of 1-phenylnaphthalene-2,3-dicarboxylic acid-d[2] S7
3. Pseudo three-component synthesis of 1*H*-benzo[*f*]isoindole-1,3(2*H*)-diones **4** (GP2) S9
3.1. 2,4-Diphenyl-1*H*-benzo[*f*]isoindole-1,3(2*H*)-dione (**4a**) S13
3.2. 6-Methoxy-4-(4-methoxyphenyl)-2-phenyl-1*H*-benzo[f]isoindole-1,3(2*H*)-dione (**4b**) S14
3.3. 2-((10-Methyl-10*H*-phenothiazin-3-yl)methyl)-4-phenyl-1*H*-benzo[*f*]isoindole-1,3(2*H*)-dione (**4c**) S14
3.4. 4-(4-Cyanophenyl)-1,3-dioxo-2-phenyl-2,3-dihydro-1H-benzo[f]isoindole-6-carbonitrile (**4d**) S15
3.5. 6-Chloro-4-(4-chlorophenyl)-2-phenyl-1*H*-benzo[*f*]isoindole-1,3(2*H*)-dione (**4e**) S15
3.6. 2-Phenyl-6-(trifluoromethyl)-4-(4-(trifluoromethyl)phenyl)-1*H*-benzo[*f*]isoindole-1,3(2*H*)-dione (**4f**) S16
3.7. 6-Methyl-2-phenyl-4-(4-tolyl)-1*H*-benzo[*f*]isoindole-1,3(2*H*)-dione (**4g**) S16
3.8. 2-(4-Fluorophenyl)-4-phenyl-1*H*-benzo[*f*]isoindole-1,3(2*H*)-dione (**4h**) S17
3.9. 2-(4-Chlorophenyl)-4-phenyl-1*H*-benzo[*f*]isoindole-1,3(2*H*)-dione (**4i**) S17
3.10. 2-(4-Iodophenyl)-4-phenyl-1*H*-benzo[*f*]isoindole-1,3(2*H*)-dione (**4j**) S18
3.11. Ethyl 4-(1,3-dioxo-4-phenyl-1,3-dihydro-2*H*-benzo[*f*]isoindol-2-yl)benzoate (**4k**) S18

3.12. 2-(3,5-Dimethylphenyl)-4-phenyl-1*H*-benzo[*f*]isoindole-1,3(2*H*)-dione (**4l**) S19
3.13. 2-(2,6-Dimethylphenyl)-4-phenyl-1*H*-benzo[*f*]isoindole-1,3(2*H*)-dione (**4m**) S19
3.14. 2-(3,5-Dimethoxyphenyl)-4-phenyl-1*H*-benzo[*f*]isoindole-1,3(2*H*)-dione (**4n**) S20
3.15. 2-Benzyl-4-phenyl-1*H*-benzo[*f*]isoindole-1,3(2*H*)-dione (**4o**) S20
3.16. 4-Phenyl-2-(prop-2-yn-1-yl)-1*H*-benzo[*f*]isoindole-1,3(2*H*)-dione (**4p**) S21
3.17. 2-*n*-Hexyl-4-phenyl-1*H*-benzo[*f*]isoindole-1,3(2*H*)-dione (**4q**) S21
3.18. 2-*n*-Butyl-4-phenyl-1*H*-benzo[*f*]isoindole-1,3(2*H*)-dione (**4r**) S22
3.19. 2-(2,6-Dimethylphenyl)-6-methoxy-4-(4-methoxyphenyl)-1H-benzo[*f*]isoindole-1,3(2*H*)-dione (**4s**) S22
3.20. Synthesis of (*E*)-2,9-diphenyl-3-(phenylimino)-2,3-dihydro-1*H*-benzo[*f*]isoindol-1-one (**5**) S23
3.21. Synthesis of 6-phenyl-12*H*-benzo[*f*]benzo[4,5]-imidazo[2,1-*a*]isoindol-12-one (**6**) S24
4. NMR spectra of compounds **2**, **4**, **5**, and **6** S26
4.1. 4-Phenylnaphtho[2,3-*c*]furan-1,3-dione (**2a**) S26
4.2. 6-Methoxy-4-(4-methoxyphenyl)naphtho[2,3-*c*]furan-1,3-dione (**2b**) S27
4.3. 6-Methyl-4-(4-tolyl)naphtho[2,3-*c*]furan-1,3-dione (**2c**) S28
4.4. 6-Chloro-4-(4-chlorophenyl)naphtho[2,3-*c*]furan-1,3-dione (**2d**) S29
4.5. NMR spectra of the diacid resulting from the hydrolysis of compound **2a** S30
4.6. 2,4-Diphenyl-1*H*-benzo[*f*]isoindole-1,3(2*H*)-dione (**4a**) S31
4.7. 6-Methoxy-4-(4-methoxyphenyl)-2-phenyl-1H-benzo[f]isoindole-1,3(2H)-dione (**4b**) S32
4.8. 2-((10-Methyl-10*H*-phenothiazin-3-yl)methyl)-4-phenyl-1*H*-benzo[*f*]isoindole-1,3(2*H*)-dione (**4c**)... S33
4.9. 4-(4-Cyanophenyl)-1,3-dioxo-2-phenyl-2,3-dihydro-1*H*-benzo[*f*]isoindole-6-carbonitrile (**4d**) S34
4.10. 6-Chloro-4-(4-chlorophenyl)-2-phenyl-1*H*-benzo[*f*]isoindole-1,3(2*H*)-dione (**4e**) S35
4.11. 2-Phenyl-6-(trifluoromethyl)-4-(4-(trifluoromethyl)phenyl)-1*H*-benzo[*f*]isoindole-1,3(2*H*)-dione (**4f**) S36
4.12. 6-Methyl-2-phenyl-4-(4-tolyl)-1*H*-benzo[*f*]isoindole-1,3(2*H*)-dione (**4g**) S37
4.13. 2-(4-Fluorophenyl)-4-phenyl-1*H*-benzo[*f*]isoindole-1,3(2*H*)-dione (**4h**) S38
4.14. 2-(4-Chlorophenyl)-4-phenyl-1*H*-benzo[*f*]isoindole-1,3(2*H*)-dione (**4i**) S39
4.15. 2-(4-Iodophenyl)-4-phenyl-1*H*-benzo[*f*]isoindole-1,3(2*H*)-dione (**4j**) S40
4.16. Ethyl-4-(1,3-dioxo-4-phenyl-1,3-dihydro-2*H*-benzo[*f*]isoindol-2-yl)benzoate (**4k**) S41
4.17. 2-(3,5-Dimethylphenyl)-4-phenyl-1*H*-benzo[*f*]isoindole-1,3(2*H*)-dione (**4l**) S42
4.18. 2-(2,6-Dimethylphenyl)-4-phenyl-1*H*-benzo[*f*]isoindole-1,3(2*H*)-dione (**4m**) S43
4.19. 2-(3,5-Dimethoxyphenyl)-4-phenyl-1*H*-benzo[*f*]isoindole-1,3(2*H*)-dione (**4n**) S44
4.20. 2-Benzyl-4-phenyl-1*H*-benzo[*f*]isoindole-1,3(2*H*)-dione (**4o**) S45
4.21. 4-Phenyl-2-(prop-2-yn-1-yl)-1*H*-benzo[*f*]isoindole-1,3(2*H*)-dione (**4p**) S46
4.22. 2-*n*-Hexyl-4-phenyl-1*H*-benzo[*f*]isoindole-1,3(2*H*)-dione (**4q**) S47
4.23. 2-*n*-Butyl-4-phenyl-1*H*-benzo[*f*]isoindole-1,3(2*H*)-dione (**4r**) S48
4.24. 2-(2,6-Dimethylphenyl)-6-methoxy-4-(4-methoxyphenyl)-1*H*-benzo[*f*]isoindole-1,3(2*H*)-dione (**4s**) S49
4.25. (*E*)-2,9-Diphenyl-3-(phenylimino)-2,3-dihydro-1*H*-benzo[*f*]isoindol-1-one (**5**) S50
4.26. 6-Phenyl-12*H*-benzo[*f*]benzo[4,5]-imidazo[2,1-*a*]isoindol-12-one (**6**) S51
5. X-ray structure analyses of compounds **4b**, **5**, and **6** S52
5.1. X-ray structure analysis of 6-methoxy-4-(4-methoxyphenyl)-2-phenyl-1*H*-benzo[f]isoindole-1,3(2H)-dione (**4b**) S52
5.2. X-ray structure analysis of (*E*)-2,9-diphenyl-3-(phenylimino)-2,3-dihydro-1*H*-benzo[*f*]isoindol-1-one (**5**) S54
5.3. X-ray structure snalysis of 6-phenyl-12*H*-benzo[*f*]benzo[4,5]-imidazo[2,1-*a*]isoindol-12-one (**6**) S55
6. Hammett-Taft correlations of compounds **4a**,**b**,**d–f** S57

1. Experimental considerations

All reactions were carried out in flame-dried glassware under nitrogen atmosphere. Reagents and catalyst were purchased reagent-grade and used without further purification. Besides triethylamine was dried with calcium hydride and stored over potassium hydroxide under nitrogen atmosphere. Solvents were dried by a solvent purification system (MB-SPS-800). T3P® (*w* = 50%) in DCE were provided by Archimica, Frankfurt, for research. Further purification of the compounds was performed with flash column chromatography (silica gel 60, mesh 230–400). TLC: silica coated aluminium plates (60, F254). ^{1}H, ^{13}C, DEPT and NOESY NMR spectra were recorded in $(CD_3)_2SO$, D_2O, DCl, $CDCl_3$ or CD_2Cl_2 on 300 MHz (Bruker AVIII) or 600 MHz (BrukerAvance III-600) NMR spectrometers. The assignments of C_{quat}, CH, CH_2 and CH_3 nuclei were based on DEPT spectra. The elemental analyses were carried out in the microanalytical laboratory on a Perkin Elmer Series ii Analyser 2400 of the Pharmazeutisches Institut of the Heinrich-Heine-Universität Düsseldorf. Mass spectra were recorded with a GC–MS-spectrometer Finnigan Trace DSQ with Finnigan Trace GC Ultra (Thermo Electron Corp.). High-resolution mass spectra were measured on a UHR-QTOF maxis 4G (Bruker Daltonics). Infrared spectra were recorded with a Shimadzu IR Affinity-1 with ATR technique. The intensities of signals are abbreviated as s (strong), m (medium) and w (weak). Uncorrected melting points and decomposition temperature were determine with Reichert Thermovar melting point microscope (heating unit: PeakTech 6000A DC Power Supply; thermometry: Norma D2400 (digital)) using the method of Kofler.[1]

Absorption spectra were recorded in various spectroscopy grade solvents at 293 K on a PerkinElmer UV/VIS/NIR Lambda 19 spectrometer. Emission spectra were recorded at 293 K on a Hitachi F7000 spectrometer. The molar extinction coefficients were determined from a multipoint setup.

2. Domino synthesis and analytical data of 4-arylnaphtho[2,3-c]furan-1,3-diones 2 (GP1)

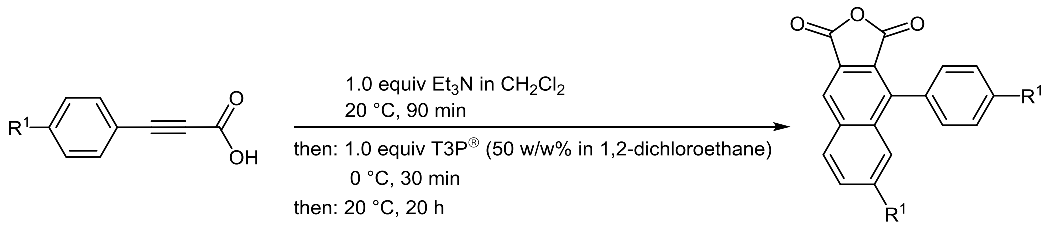

1 **2a-d**

In a Schlenk tube the 3-arylpropiolic acid **1** (2.00 mmol) was dissolved in dichloromethane (4.00 mL) under a nitrogen atmosphere (for experimental details, see Table S1). Then dry triethylamine (203 mg, 2.01 mmol) was added to the solution. The solution were stirred at room temp for 90 min and then cooled to 0 °C (ice bath). After 10 min the dropwise addition of T3P® (1.27 g, 2.00 mmol; 50 wt % in dichloroethane) was started. After complete addition of T3P® the reaction mixture was stirred at 0 °C for 15 min. Subsequently the ice bath was removed and the reaction mixture was stirred at room temp for 20 h. Then the crude product was diluted with dichloromethane and washed with a saturated aqueous solution of $NaHCO_3$ (2 × 20 mL). The organic layer was dried (anhydrous Na_2SO_4) and the solvents were removed in vacuo.

Table S1. Experimental details for the synthesis of 4-arylnaphtho[2,3-*c*]furan-1,3-diones **2**.

Entry	Propiolic acid **1** [mg] (mmol)	4-arylnaphtho[2,3-*c*]furan-1,3-diones **2** yield [mg] (%)
1	291 (1.99) of **1a** (R^1 = H)	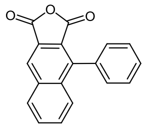 274 (100) of **2a**
2	352 (2.00) of **1b** (R^1 = OCH_3)	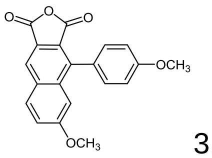 315 (94) of **2b**
3[a]	90.1 (0.56) of **1c** (R^1 = CH_3)	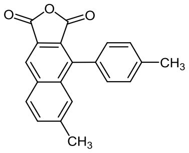 77.6 (92) of **2c**
4[b]	164 (0.91) of **1d** (R^1 = Cl)	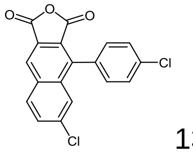 138 (88) of **2d**

[a]Reaction with triethylamine (57.6 mg, 0.57 mmol), dichloromethane (1.10 mL), T3P® (359 mg, 0.56 mmol; 50 wt % in 1,2-dichloroethane). [b]Reaction with triethylamine (93.3 mg, 0.92 mmol), dichloromethane (1.80 mL), T3P® (578 mg, 0.91 mmol; 50 wt % in 1,2-dichloroethane).

2.1. 4-Phenylnaphtho[2,3-*c*]furan-1,3-dione (2a)

According to GP1 compound **2a** (274 mg, 100%) was obtained as a colorless solid, Mp 255 °C (dec.) (lit.:[2] 260-262 °C). ^{1}H NMR (600 MHz, $(CD_3)_2SO$): δ 7.44-7.48 (m, 2 H), 7.55-7.60 (m, 3 H), 7.76 (d, *J* = 8.5 Hz, 1 H), 7.78-7.82 (m, 1 H), 7.85-7.89 (m, 1 H), 8.41 (d, *J* = 8.1 Hz, 1 H), 8.82 (s, 1 H). ^{13}C NMR (151 MHz, $(CD_3)_2SO$): δ 122.9 (C_{quat}), 126.1 (C_{quat}), 126.9, 127.8, 128.3, 128.8, 129.9, 130.0, 130.6, 130.9, 133.4 (C_{quat}), 134.8 (C_{quat}), 135.8 (C_{quat}), 141.6 (C_{quat}), 162.1 (C_{quat}), 163.3 (C_{quat}). IR (ATR): $\tilde{\nu}$ 3100 (w), 1829 (m), 1771 (s), 1636 (w), 1607 (m), 1539 (w), 1516 (m), 1491 (w), 1472 (w), 1408 (m), 1369 (m), 1331 (w), 1296 (w), 1258 (w), 1238 (s), 1225 (m), 1215 (m), 1188 (m), 1177 (m), 1148 (m), 1123 (m), 1072 (m), 1028 (w), 939 (s), 901 (s), 849 (m), 831 (m), 810 (m), 768 (s), 750 (m), 735 (s), 696 (s), 633 (m), 611 (m). EI MS (70 eV): *m/z* (%) 275 (11), 274 (M^+, 56), 230 (M^+-CO_2, 11), 211 (12), 209 (50), 207 (38), 203 ($C_{16}H_{11}^+$, 17), 202 ($C_{16}H_{10}^+$, 100), 201 (25), 200 (26), 174 (25), 172 (23), 148 (28), 147 (47), 129 (22), 128 (18), 103 (17), 101 (18), 100 (15), 77 ($C_6H_5^+$, 14). HR MS (ESI) calcd. for $C_{18}H_{10}O_3$+H^+: 275.0703; Found: 275.0708.

2.2. 6-Methoxy-4-(4-methoxyphenyl)naphtho[2,3-*c*]furan-1,3-dione (2b)

According to GP1 compound **2b** (315 mg, 94%) was obtained as a yellow solid, Mp 214 °C (dec.) (lit.:[3] 216-217 °C). ^{1}H NMR (600 MHz, $(CD_3)_2SO$): δ 3.75 (s, 3 H), 3.86 (s, 3 H), 7.12 (d, *J* = 8.7 Hz, 2 H), 7.14 (d, *J* = 2.5 Hz, 1 H), 7.42 (d, *J* = 8.6 Hz, 2 H), 7.55 (dd, *J* = 9.0, 2.5 Hz, 1 H), 8.32 (d, *J* = 9.0 Hz, 1 H), 8.70 (s, 1 H). ^{13}C NMR (151 MHz, $(CD_3)_2SO$): δ 55.2 (CH_3), 55.4 (CH_3), 106.9, 113.8, 121.6, 123.4 (C_{quat}), 123.7 (C_{quat}), 125.2 (C_{quat}), 126.5, 131.1 (C_{quat}), 131.4, 132.7, 137.2 (C_{quat}), 139.9 (C_{quat}), 159.6 (C_{quat}), 160.5 (C_{quat}), 162.4 (C_{quat}), 163.4 (C_{quat}). IR (ATR): $\tilde{\nu}$ 3108 (w), 3067 (w), 3021 (w), 1830 (m), 1800 (w), 1767 (s), 1714 (w), 1684 (w), 1607 (m), 1593 (m), 1574 (w), 1510 (s), 1483 (w), 1429 (m), 1420 (m), 1398 (m), 1377 (m), 1327 (m), 1306 (m), 1292 (m), 1258 (m), 1209 (s), 1170 (m), 1167 (m), 1142 (m), 1111 (m), 1074 (m), 1030 (s), 982 (m), 908 (s), 866 (m), 840 (m), 827 (m), 799 (m), 783 (m), 741 (s), 698 (m), 679 (m), 633 (m). EI MS (70 eV): *m/z* (%) 335 (22), 334 (M^+, 100), 262 ($C_{18}H_{14}O_2^+$, 11), 247 (43), 204 (10), 176 (37), 167 (12), 159 (31), 149 (25), 139 (11), 137 (11), 132 (10), 125 (16), 124 (14), 113 (12), 111 (26), 109 (16), 99 (15), 97 (34), 96 (13), 95 (23), 86 (17), 85 (28), 84 (11), 83 (28), 82 (11), 81 (20), 71 (36), 70 (12), 69 (28), 67 (11), 58 (10), 57 (63), 56 (12), 55 (39), 44 (11), 43

(48), 41 (29), 40 (42). Anal. calcd. for $C_{20}H_{14}O_5$ (334.1): C 71.85, H 4.22; Found: C 71.57, H 4.43.

2.3. 6-Methyl-4-(4-tolyl)naphtho[2,3-*c*]furan-1,3-dione (2c)

According to GP1 compound **2c** (77.6 mg, 91%) was obtained as a beige solid, 269 °C (dec.) (lit.:[3] 266-267 °C). ^{1}H NMR (600 MHz, $(CD_3)_2SO$): δ 2.45 (s, 3 H), 2.46 (s, 3 H), 7.34 (d, J = 7.9 Hz, 2 H), 7.39 (d, J = 7.8 Hz, 2 H), 7.57 (s, 1 H), 7.72 (d, J = 8.4, 1.7 Hz, 1 H), 8.31 (d, J = 8.4 Hz, 1 H), 8.75 (s, 1 H). ^{13}C NMR (151 MHz, $(CD_3)_2SO$): δ 21.0 (CH_3), 21.8 (CH_3), 122.9 (C_{quat}), 125.2 (C_{quat}), 126.55, 126.61, 128.9, 129.8, 130.4 (C_{quat}), 130.8, 132.1, 134.1 (C_{quat}), 135.2 (C_{quat}), 138.1 (C_{quat}), 140.8 (C_{quat}), 141.1 (C_{quat}), 162.2 (C_{quat}), 163.4 (C_{quat}). IR (ATR): $\tilde{\nu}$ 3026 (w), 2961 (m), 2916 (w), 2855 (w), 1869 (w), 1832 (m), 1796 (m), 1763 (s), 1753 (s), 1736 (m), 1609 (m), 1508 (m), 1429 (m), 1404 (m), 1373 (m), 1319 (m), 1292 (m), 1260 (m), 1229 (s), 1194 (m), 1184 (m), 1157 (m), 1148 (m), 1125 (m), 1107 (m), 1084 (m), 1043 (m), 966 (m), 937 (w), 899 (s), 887 (s), 868 (s), 841 (m), 814 (s), 787 (s), 737 (s), 718 (m), 700 (s), 669 (m), 629 (s). EI MS (70 eV): *m/z* (%) 303 (22), 302 (M^+, 99), 287 ($C_{19}H_{11}O_3^+$, 23), 243 (47), 231 (10), 230 ($C_{18}H_{14}^+$, 55), 229 (64), 228 (34), 227 (21), 226 (31), 216 (19), 215 ($C_{17}H_{11}^+$, 100), 214 (10), 213 (24), 202 (22), 189 (16), 150 (14), 113 (14), 108 (11), 101 (13), 95 (19), 71 (11), 57 (15), 55 (11), 43 (11). HR MS (ESI): calcd. for $C_{20}H_{14}O_3$+H^+: 303.1016; Found: 303.1018.

2.4. 6-Chloro-4-(4-chlorophenyl)naphtho[2,3-*c*]furan-1,3-dione (2d)

According to GP1 compound **2d** (138 mg, 88%) were obtained as a light yellow solid, Mp 263 °C (dec) (lit.:[4] 262-263 °C). ^{1}H NMR (600 MHz, $(CD_3)_2SO$): δ 7.53 (d, J = 8.5 Hz, 2 H), 7.64-7.74 (m, 3 H),7.96 (dd, J = 8.8, 2.1 Hz, 1 H), 8.84 (d, J = 8.9 Hz, 1 H), 8.91 (s, 1 H). IR (ATR): $\tilde{\nu}$ 3238 (w), 3086 (w), 2961 (w), 2924 (w), 1848 (m), 1829 (m), 1786 (s), 1761 (m), 1643 (m), 1599 (m), 1522 (w), 1493 (s), 1416 (m), 1391 (m), 1364 (m), 1314 (m), 1288 (s), 1261 (m), 1231 (s), 1217 (s), 1209 (s), 1180 (m), 1152 (m), 1132 (s), 1076 (s), 1016 (m), 959 (s), 939 (m), 907 (s), 889 (s), 856 (s), 814 (s), 800 (s), 766 (s), 739 (s), 716 (m), 652 (m), 615 (s). EI MS (70 eV): *m/z* (%) 344 (M^+($^{37}Cl^{37}Cl$), 39), 343 (M^+($^{37}Cl^{35}Cl$), 13), 342 (M^+($^{35}Cl^{35}Cl$), 58), 309 (7), 308 (4), 307 (20), 273 (10), 272 (M^+-Cl_2, 54), 271 (19), 270 (90), 265 (13), 263 (38), 237 (17), 236 (12), 235 (51), 234 (12), 201 (16), 200 ($C_{16}H_8^+$, 100), 199 (36), 198 (32), 174 (12), 150 (10), 123 (11), 122 (11), 117 (11), 111 (11), 100 (33), 99 (28), 98 (14), 87 (14), 86 (11), 75 (15), 74 (17). HR MS (ESI) calcd. for $C_{18}H_8Cl_2O_3$+H^+: 342.9923; Found: 342.9922.

2.5. Hydrolysis of compound 2a and analytical data of 1-phenylnaphthalene-2,3-dicarboxylic acid-d_2

The anhydride **2a** was dissolved in DMSO-d_6 and after the addition of an excess of DCl in D_2O ^{1}H NMR spectra were measured instaneous, 60 min and 48 h after addition of DCl.

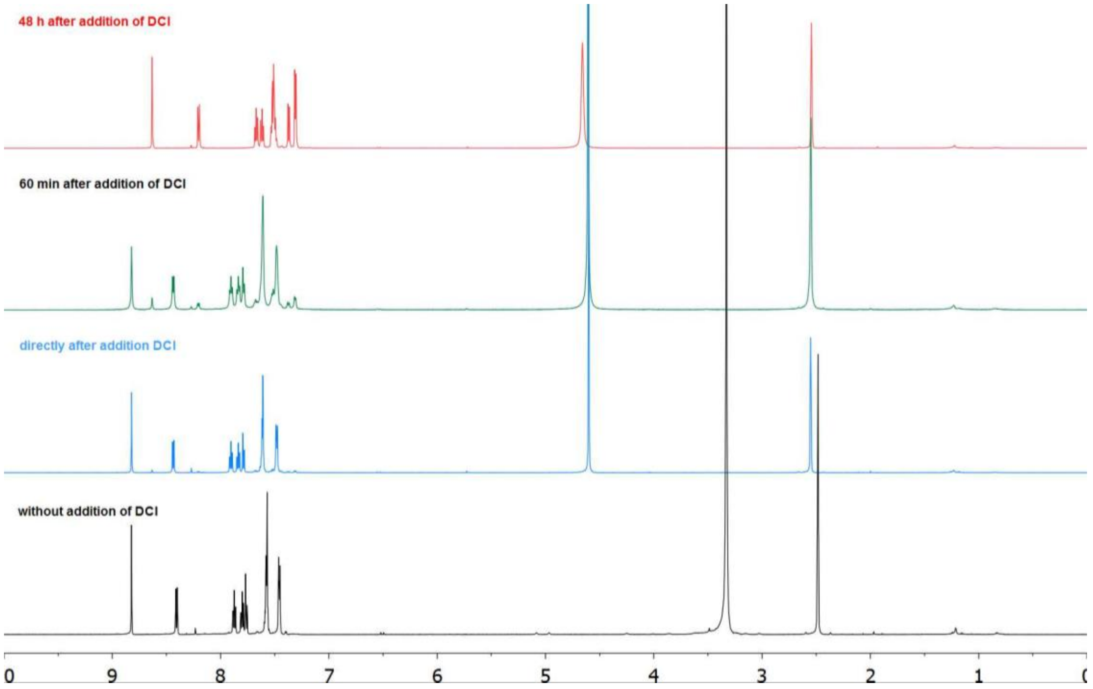

The conversion was quantified by the integration of characteristic signals of the anhydride **2a** and the dicarboxlic acid (Table S2).

Table S2. Percentage of compound **2a** and the diacid.

Addition DCl	**2a**	**diacid**
without	100%	-
directly	97%	3%
after 60 min	80%	20%
after 48 h	-	100%

After 48 h the ^{1}H NMR spectrum of the pure deuterated dicarboxylic acid was detected.

^{1}H NMR (600 MHz, $(CD_3)_2SO$ +1.0 equiv of DCl (in D_2O)): δ 7.30 (m, 2 H), 7.37 (d, J = 8.4 Hz, 1 H), 7.50-7.52 (m, 3 H), 7.61-7.63 (m, 1 H), 7.66-7.68 (m, 1 H), 8.20 (d, J = 8.2 Hz, 1 H), 8.63 (s, 1 H).

The anhydride **2a** (274 mg, 1.00 mmol) was dissolved in dry acetone (2.50 mL). After addition of an aqueous solution of ammonium chloride (2.50 mL, 0.40 mM) the reaction mixture was stirred at room temp for 16 h. Then the solvents of the crude product were evaporated and the solid was dried for 48 h under vacuo. Under these conditions, the hydrolysis of the anhydride **2a** was not complete but the characteristic set of signals of the dicarboxylic acid was unabiguously indentified in the ^{13}C NMR spectrum.

^{13}C NMR (151 MHz, $(CD_3)_2SO$): δ 125.6 (C_{quat}), 126.0, 127.3, 127.8, 128.0, 129.0, 129.4, 130.1, 130.7, 131.7 (C_{quat}), 132.3 (C_{quat}), 133.2 (C_{quat}), 136.7 (C_{quat}), 136.8 (C_{quat}), 167.0 (C_{quat}), 169.1 (C_{quat}).

3. Pseudo three-component synthesis of 1*H*-benzo[*f*]isoindole-1,3(2*H*)-diones 4 (GP2)

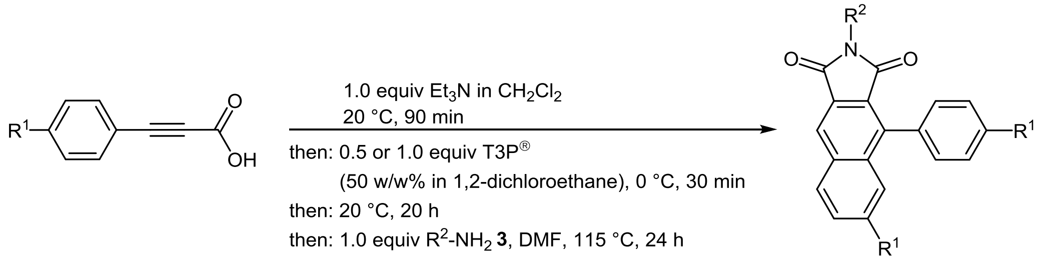

1 **4**

In a Schlenk tube the 3-arylpropiolic acid **1** (2.00 mmol) was dissolved in dichloromethane (4.00 mL) under a nitrogen atmosphere (for experimental details, see Table S3). Then dry triethylamine (203 mg, 2.01 mmol) was added to the solution. The solution were stirred at room temp for 90 min and then cooled to 0 °C (ice bath). After 10 min the dropwise addition of T3P® (1.27 g, 2.00 mmol or 0.63 g, 1.00 mmol; 50 wt % in dichloroethane) was started. After complete addition of T3P® the reaction mixture was stirred at 0 °C for 30 min and then allowed to come to room temp and stirred for 20 h. Then DMF (4.00 mL) and amine **3** (2.00 mmol) were added to the reaction mixture. The reaction mixture was heated to 115 °C for 24 h. After cooling to room temp the reaction mixture was diluted with dichloromethane and adsorbed on Celite®. The crude product **4** was purified by column chromatography on silica gel.

Table S3. Experimental details for the pseudo three-component synthesis of 1*H*-benzo[*f*]isoindole-1,3(2*H*)-diones **4**.

Entry	Propiolic acid **1** [mg] (mmol)	Amine **3** [mg] (mmol)	1*H*-Benzo[*f*]isoindole-1,3(2*H*)-dione **4** yield [mg] (%)
1[a]	293 (2.00) of **1a** (R^1 = H)	189 (2.03) of **3a** (R^2 = C_6H_5**)**	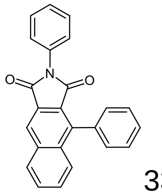 333 (95) of **4a**
2[a]	353 (2.00) of **1b** (R^1 = OCH_3)	186 (2.00) of **3a**	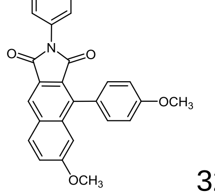 323 (79) of **4b**

Entry	Propiolic acid **1** [mg] (mmol)	Amine **3** [mg] (mmol)	1*H*-Benzo[*f*]isoindole-1,3(2*H*)-dione **4** yield [mg] (%)
3[a,b]	292 (2.00) of **1a**	557 (2.00) of **3b** **(**R^2 = CH_2(10-methyl-10*H*-phenothiazine-3-yl))	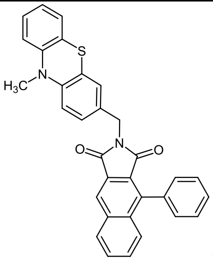 425 (85) of **4c**
4[a,c]	169 (0.99) of **1e** (R^1 = CN)	94.7 (1.02) of **3a**	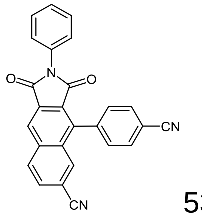 53.6 (27) of **4d**
5[a,c,e]	342 (2.00) of **1e**	189 (2.03) of **3a**	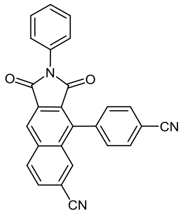 165 (41) of **4d**
6[a,d,f]	164 (0.91) of **1d** (R^1 = Cl)	93.1 (1.00) of **3a**	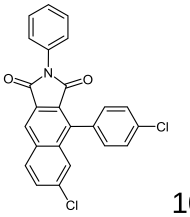 101 (53) of **4e**
7[a,d,g]	214 (1.00) of **1f** (R^1 = CF_3)	83.8 (0.90) of **3a**	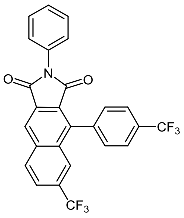 157 (65) of **4f**
8[h]	292 (2.00) of **1a**	186 (2.00) of **3a**	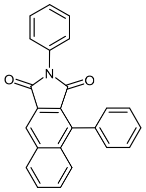 320 (92) of **4a**

Entry	Propiolic acid **1** [mg] (mmol)	Amine **3** [mg] (mmol)	1*H*-Benzo[*f*]isoindole-1,3(2*H*)-dione **4** yield [mg] (%)
9[h]	320 (2.00) of **1c**	186 (2.00) of **3a**	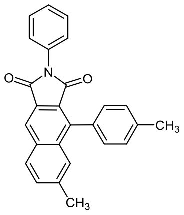 181 (48) of **4g**
10[h]	292 (2.00) of **1a**	224 (2.00) of **3c** (R^2 = *p*-FC_6H_4)	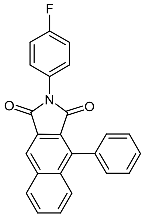 201 (55) of **4h**
11[h]	292 (2.00) of **1a**	255 (2.00) of **3d** (R^2 = *p*-ClC_6H_4)	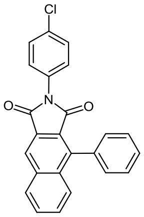 250 (65) of **4i**
12[h]	292 (2.00) of **1a**	438 (2.00) of **3e** (R^2 = *p*-IC_6H_4)	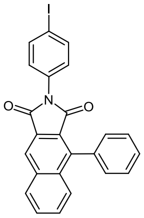 241 (51) of **4j**
13[h]	292 (2.00) of **1a**	324 (2.00) of **3f** (R^2 = *p*-$EtO_2CC_6H_4$)	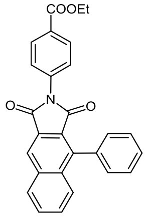 240 (57) of **4k**
14[h]	292 (2.00) of **1a**	243 (2.00) of **3g** (R^2 = 3,5-$Me_2C_6H_3$)	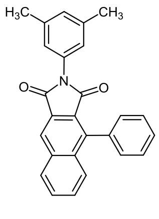 260 (69) of **4l**

Entry	Propiolic acid **1** [mg] (mmol)	Amine **3** [mg] (mmol)	1*H*-Benzo[*f*]isoindole-1,3(2*H*)-dione **4** yield [mg] (%)
15[h]	292 (2.00) of **1a**	241 (2.00) of **3h** (R^2 = 2,6-$Me_2C_6H_3$)	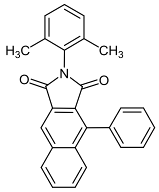 211 (56) of **4m**
16[h]	292 (2.00) of **1a**	306 (2.00) of **3i** (R^2 = 3,5-$(MeO)_2C_6H_3$)	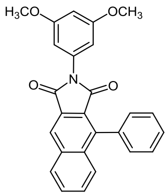 261 (64) of **4n**
17[h]	292 (2.00) of **1a**	215 (2.00) of **3j** (R^2 = $CH_2C_6H_5$)	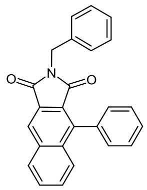 150 (41) of **4o**
18[h]	292 (2.00) of **1a**	355 (2.00) of **3k** (R^2 = $CH_2C\equiv CH$)	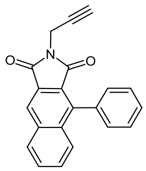 81 (26) of **4p**
19[h]	292 (2.00) of **1a**	203 (2.01) of **3l** (R^2 = *n*-hexyl)	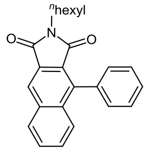 190 (53) of **4q**
20[h]	292 (2.00) of **1a**	146 (2.00) of **3m** (R^2 = *n*-butyl)	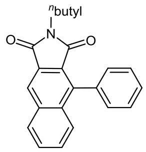 200 (61) of **4r**

Entry	Propiolic acid **1** [mg] (mmol)	Amine **3** [mg] (mmol)	1*H*-Benzo[*f*]isoindole-1,3(2*H*)-dione **4** yield [mg] (%)
21[h]	352 (2.00) of **1b**	242 (2.00) of **3h**	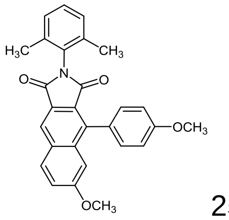 250 (57) of **4s**

[a]The reaction was performed with 1.00 equiv of T3P®. [b]Compound **3b** was employed as the corresponding hydrochloride with 202 mg (2.00 mmol) triethylamine in dichloromethane (1.0 mL) at rt for 1 h. [c]The reaction was performed with triethylamine (101 mg, 1.00 mmol), dichloromethane (2.00 mL), and T3P® (632 mg**,** 0.99 mmol**;** 50 wt % in 1,2-dichloroethane). [d]The formation of the anhydride **2** was achieved after stirring at room temp for 20 h. The reaction mixture was diluted with dichloromethane and the organic phase was washed with a saturated aqueous solution of $NaHCO_3$ (2 × 20 mL). The organic layer was dried (anhydrous Na_2SO_4) and the solvents were removed in vacuo. Then DMF and the amine **3** were added to the reaction mixture. [e]Reaction with triethylamine (92.0 mg, 0.91 mmol), dichloromethane (1.80 mL), T3P® (580 mg, 0.91 mmol; 50 wt % in 1,2-dichloroethane). [f]Reaction with triethylamine (101 mg, 1.00 mmol), dichloromethane (2.00 mL), T3P® (634 mg, 1.00 mmol; 50 wt % in 1,2-dichloroethane). [g]The formation of the anhydride **2** was achieved after stirring at room temp for 20 h. [h]The reaction was performed with 0.50 equiv of T3P®.

3.1. 2,4-Diphenyl-1*H*-benzo[*f*]isoindole-1,3(2*H*)-dione (4a)

According to GP2 and after purification of the crude product by column chromatography (*n*-hexane/ethyl acetate 10:1) compound **4a** (applying 1.00 equiv of T3P®: 333 mg, 95%; applying 0.50 equiv of T3P®: 320 mg, 92%) was obtained as a light yellow solid, R_f (*n*-hexane:EtOAc, 5:1): 0.46, Mp 218 °C (lit.:[5] 227-228 °C). ^{1}H NMR (300 MHz, $CDCl_3$): δ 7.32-7.41 (m, 1 H), 7.42-7.50 (m, 6 H), 7.51-7.59 (m, 3 H), 7.63 (ddd, *J* = 8.3, 6.9, 1.4 Hz, 1 H), 7.72 (ddd, *J* = 8.2, 6.9, 1.4 Hz, 1 H), 7.87 (dd, *J* = 8.2, 1.4 Hz, 1 H), 8.14 (dd, *J* = 8.1, 1.4 Hz, 1 H), 8.50 (s, 1 H). ^{13}C NMR (75 MHz, $CDCl_3$): δ 123.4 (C_{quat}), 124.8, 126.7, 127.5 (C_{quat}), 128.0, 128.2, 128.6, 128.7, 128.9, 129.1, 129.3, 129.8, 130.4, 131.8 (C_{quat}), 134.4 (C_{quat}), 135.66 (C_{quat}), 135.71 (C_{quat}), 140.9 (C_{quat}), 166.4 (C_{quat}), 166.9 (C_{quat}). IR (ATR): $\tilde{\nu}$ 3061 (w), 2924 (w), 1767 (m), 1748 (m), 1713 (s), 1684 (m), 1593 (m), 1516 (w), 1491 (m), 1435 (m), 1402 (m), 1368 (s), 1356 (s), 1329 (m), 1296 (w), 1256 (m), 1206 (m), 1173 (w), 1111 (m), 1099 (m), 1072 (m), 1011 (m), 995 (m), 943 (m), 899 (m), 872 (w), 851 (w), 839 (w), 793 (m), 766 (s), 752 (s), 737 (s), 702 (s), 689 (s), 669 (m), 640 (m). EI MS (70 eV): *m/z* (%) 349 (M^+, 20), 348 (15), 304 (14), 288 (21), 287 (100), 286 (47), 273 (25), 272 ($C_{18}H_{10}NO_2^+$, 85), 259 (10), 258 (13), 244 (10), 242 (18), 230 (14), 229 (41), 228 (12), 227 (22), 203 (11), 202 ($C_{16}H_{10}^+$, 50), 201 (41), 200 (32), 167 (19), 150 (10),

149 ($C_8H_7O_2^+$, 45), 127 (11), 115 (12), 111 (15), 105 (25), 102 (13), 101 (19), 100 (19), 99 (11), 97 (24), 95 (14), 93 (29), 88 (12), 85 (23), 83 (22), 81 (13), 77 ($C_6H_5^+$, 32), 71 (36), 70 (18), 69 (22), 57 (37), 55 (23), 43 (32), 41 (12). Anal. calcd. for $C_{24}H_{15}NO_2$ (349.1): C 82.50, H 4.33, N 4.01; Found: C 82.46, H 4.61, N 4.31.

3.2. 6-Methoxy-4-(4-methoxyphenyl)-2-phenyl-1*H*-benzo[*f*]isoindole-1,3(2*H*)-dione (4b)

According to GP2 and after purification of the crude product by column chromatography (*n*-hexane/ethyl acetate 2:1) compound **4b** (323 mg, 79%) was obtained as a deep yellow solid, R_f (*n*-hexane:EtOAc, 5:1): 0.14, Mp 238 °C (lit.:[5] 226-228 °C). ^{1}H NMR (600 MHz, $CDCl_3$): δ 3.78 (s, 3 H), 3.90 (s, 3 H), 7.08 (d, J = 8.7 Hz, 2 H), 7.20 (d, J = 2.6 Hz, 1 H), 7.35 (dd, J = 8.9, 2.6 Hz, 2 H), 7.35-7.40 (m, 2 H), 7.41-7.48 (m, 4 H), 8.01 (d, J = 9.0 Hz, 1 H), 8.39 (s, 1 H). ^{13}C NMR (151 MHz, $CDCl_3$): δ 55.4 (CH_3), 55.6 (CH_3), 107.7, 113.9, 121.3, 124.2 (C_{quat}), 124.5, 125.5 (C_{quat}), 126.6 (C_{quat}), 126.8, 128.0, 129.0, 131.0 (C_{quat}), 131.3, 131.9, 132.1 (C_{quat}), 138.1 (C_{quat}), 139.4 (C_{quat}), 159.9 (C_{quat}), 160.4 (C_{quat}), 166.9 (C_{quat}), 167.2 (C_{quat}). IR (ATR): $\tilde{\nu}$ 3055 (w), 2959 (w), 2922 (w), 2843 (w), 1767 (m), 1713 (s), 1705 (s), 1682 (m), 1578 (m), 1512 (s), 1502 (s), 1491 (s), 1427 (m), 1417 (m), 1400 (m), 1366 (s), 1323 (m), 1312 (m), 1288 (m), 1242 (s), 1231 (s), 1194 (s), 1184 (s), 1175 (m), 1161 (m), 1121 (s), 1103 (s), 1084 (m), 1067 (m), 1026 (s), 1001 (m), 980 (m), 959 (w), 943 (w), 901 (s), 847 (s), 816 (s), 804 (s), 787 (m), 768 (m), 756 (s), 729 (s), 710 (s), 691 (s). EI MS (70 eV): *m/z* (%) 410 (31), 409 (M^+, 100), 408 (14), 364 (22), 334 (10), 176 (11). Anal. calcd. for $C_{26}H_{19}NO_4$ (409.1): C 76.27, H 4.68, N 3.42; Found: C 76.01, H 4.74, N 3.50.

3.3. 2-((10-Methyl-10*H*-phenothiazin-3-yl)methyl)-4-phenyl-1*H*-benzo[*f*]isoindole-1,3(2*H*)-dione (4c)

According to GP2 and after purification of the crude product by column chromatography (*n*-hexane/ethyl acetate 3:1) compound **4c** (425 mg, 85%) was obtained as a light yellow solid, R_f (*n*-hexane:EtOAc, 2:1): 0.61, Mp > 250 °C. ^{1}H NMR (300 MHz, CD_2Cl_2): δ 3.31 (s, 3 H), 4.70 (s, 2 H), 6.75 (d, J = 8.3 Hz, 1 H), 6.79 (dd, J = 8.1, 0.9 Hz, 1 H), 6.87-6.93 (m, 1 H), 7.09 (dd, J = 7.6, 1.5 Hz, 1 H), 7.11-7.16 (m, 1 H), 7.17 (d, J = 1.9 Hz, 1 H), 7.24 (dd, J = 8.3, 2.1 Hz, 1 H), 7.35-7.42 (m, 2 H), 7.49-7.64 (m, 4 H), 7.68 (ddd, J = 8.2, 6.9, 1.3 Hz, 1 H). 7.76 (dd, J = 8.4, 1.2 Hz, 1 H), 8.10 (dd, J = 8.1, 1.7 Hz, 1 H), 8.36 (s, 1 H). ^{1}H NMR (600 MHz, CD_2Cl_2): δ 3.31 (s, 3 H), 4.70 (s, 2 H), 6.75 (d, J = 8.3 Hz, 1 H), 6.80 (dd, J = 8.1, 1.2 Hz, 1 H), 6.89-6.91 (m, 1 H), 7.09 (dd, J = 7.7, 1.5 Hz, 1 H), 7.12-7.16 (m, 1 H), 7.17 (d, J = 2.0 Hz, 1 H), 7.24 (dd, J = 8.3, 2.0 Hz, 1 H), 7.37-7.41 (m, 2 H), 7.52-7.56 (m, 3 H), 7.59 (ddd, J = 8.3, 6.9, 1.3 Hz, 1 H),

7.68 (ddd, *J* = 8.1, 6.8, 1.2 Hz, 1 H), 7.76 (d, *J* = 8.5 Hz, 1 H), 8.10 (dd, *J* = 8.3, 1.3 Hz, 1 H), 8.36 (s, 1 H). ^{13}C NMR (75 MHz, CD_2Cl_2): δ 35.8 (CH_3), 41.4 (CH_2), 114.5, 114.6, 123.0, 123.5 (C_{quat}), 124.0 (C_{quat}),124.5 (C_{quat}), 124.6, 127.5, 127.8, 128.0, 128.4 (C_{quat}), 128.6, 128.7, 128.9, 129.0, 129.4, 129.6, 130.5, 130.8, 131.5 (C_{quat}), 135.3 (C_{quat}), 135.97 (C_{quat}), 135.99 (C_{quat}), 140.6 (C_{quat}), 146.0 (C_{quat}), 146.2 (C_{quat}), 167.5 (C_{quat}), 167.9 (C_{quat}). IR (ATR): $\tilde{\nu}$ 3063 (w), 2978 (w), 2957 (w), 2936 (w), 1760 (m), 1705 (s), 1674 (m), 1647 (w), 1578 (m), 1494 (m), 1464 (s), 1445 (m), 1427 (m), 1406 (m), 1396 (m), 1381 (m), 1325 (s), 1277 (m), 1248 (m), 1231 (w), 1209 (w), 1192 (w), 1134 (m), 1113 (m), 1074 (m), 1036 (w), 1007 (w), 951 (m), 895 (m), 885 (m), 831 (m), 797 (s), 770 (s), 748 (s), 731 (m), 700 (s), 689 (m), 677 (m), 638 (s). EI MS (70 eV): *m/z* (%) 500 (11), 499 (35), 498 (M^+, 100), 484 (11), 483 (M^+-CH_3, 28), 286 ($C_{19}H_{12}NO_2^+$, 24), 249 ($C_{14}H_{12}NS^+$, 11), 202 ($C_{16}H_{10}^+$, 14). Anal. calcd. for $C_{32}H_{22}N_2O_2S$ (498.1): C 77.09, H 4.45, N 5.62, S 6.43; Found: C 76.82, H 4.45, N 5.47, S 6.41.

3.4. 4-(4-Cyanophenyl)-1,3-dioxo-2-phenyl-2,3-dihydro-1*H*-benzo[*f*]isoindole-6-carbonitrile (4d)

According to GP2 and after purification of the crude product by column chromatography (*n*-hexane/ethyl acetate 3:1) compound **4d** (applying 1.00 equiv of T3P®: 53.6 mg, 27%; applying 0.50 equiv of T3P®: 165 mg, 41%) was obtained as a light beige solid, R_f (*n*-hexane:EtOAc, 2:1): 0.20, Mp > 250 °C. ^{1}H NMR (300 MHz, $CDCl_3$): δ 7.37-7.43 (m, 3 H), 7.44-7.52 (m, 2 H), 7.53-7.59 (m, 2 H), 7.85-7.92 (m, 3 H), 8.06 (dd, *J* = 1.6, 0.8 Hz, 1 H), 8.28 (d, *J* = 8.5 Hz, 1 H), 8.59 (s, 1 H)[1]. ^{13}C NMR (75 MHz, $CDCl_3$): δ 113.5 (C_{quat}), 113.6 (C_{quat}), 117.9 (C_{quat}), 118.3 (C_{quat}), 125.3 (C_{quat}), 125.4, 126.5, 128.7, 129.2, 130.0, 130.1 (C_{quat}), 130.8, 131.3 (C_{quat}), 131.9, 132.5, 133.4, 134.3 (C_{quat}), 137.2 (C_{quat}), 137.9 (C_{quat}), 138.6 (C_{quat}), 165.5 (C_{quat}), 165.7 (C_{quat})[1]. IR (ATR): $\tilde{\nu}$ 3143 (w), 3086 (w), 2226 (m), 1775 (m), 1724 (m), 1709 (s), 1616 (w), 1601 (w), 1593 (w), 1495 (m), 1423 (w), 1408 (w), 1342 (s), 1300 (w), 1209 (w), 1099 (m), 1059 (m), 1020 (w), 905 (m), 889 (w), 831 (s), 764 (s), 691 (m), 625 (m), 619 (s); EI MS (70 eV): *m/z* (%) 400 (30), 399 (M^+, 100), 398 (33), 355 (23), 354 (63), 253 (19), 252 ($C_{18}H_8N_2^+$, 15), 225 (11), 199 (15), 149 (11), 77 ($C_6H_5^+$, 22); HR MS (ESI) calcd for $C_{26}H_{13}N_3O_2^+$: 399.1002; Found: 399.1000; calcd for $C_{26}H_{13}N_3O_2$+H^+: 400.1081; Found: 400.1076.

3.5. 6-Chloro-4-(4-chlorophenyl)-2-phenyl-1*H*-benzo[*f*]isoindole-1,3(2*H*)-dione (4e)

According to GP2 and after purification of the crude product by column chromatography (*n*-hexane/ethyl acetate 10:1) compound **4e** (101 mg, 53%) was obtained as a light brown solid,

---

[1] Residues of DMF found in NMR spectra: ^{1}H NMR: δ 2.87 (s, 3 H), 2.94 (s, 3 H), 8.00 (s, 1 H); ^{13}C NMR: δ 31.5 (CH_3), 36.6 (CH_3), 162.6 (C_{quat}).

R_f (*n*-hexane:EtOAc, 5:1): 0.42, Mp > 250 °C (lit.:[5] 164-165 °C). ^{1}H NMR (300 MHz, CDCl$_3$): δ 7.34-7.51 (m, 7 H), 7.52-7.58 (m, 2 H), 7.68 (dd, J = 8.7, 2.1 Hz, 1 H), 7.78 (d, J = 2.1 Hz, 1 H), 8.08 (d, J = 8.7 Hz, 1 H), 8.48 (s, 1 H). ^{13}C NMR (75 MHz, CDCl$_3$): δ 124.7 (C_{quat}), 124.9, 126.7, 127.3, 127.8 (C_{quat}), 128.4, 128.9, 129.1, 130.3, 131.4, 131.7 (C_{quat}), 131.9, 132.1 (C_{quat}), 134.0 (C_{quat}), 135.3 (C_{quat}), 136.2 (C_{quat}), 136.4 (C_{quat}), 138.6 (C_{quat}), 166.1 (C_{quat}), 166.5 (C_{quat}). IR (ATR): $\tilde{\nu}$ 3042 (w), 2963 (w), 2922 (w), 1767 (m), 1712 (s), 1697 (m), 1686 (m), 1647 (w), 1593 (m), 1489 (m), 1445 (w), 1408 (m), 1395 (m), 1360 (m), 1285 (m), 1260 (m), 1194 (m), 1157 (m), 1117 (s), 1088 (s), 1020 (s), 1003 (m), 961 (m), 943 (m), 907 (s), 822 (s), 800 (s), 766 (s), 752 (s), 739 (m), 719 (m), 708 (m), 689 (s), 654 (m), 621 (s); EI MS (70 eV): *m/z* (%) 421 (13), 420 (19), 419 (M^+(^{37}Cl^{37}Cl), 63), 418 (M^+(^{37}Cl^{35}Cl), 40), 417 (M^+(^{35}Cl^{35}Cl), 100), 416 (24), 382 (11), 374 (17), 372 (27), 338 (26), 303 (10), 235 (14), 200 (36), 199 (11), 191 (13), 77 ($C_6H_5^+$, 20). HR MS (ESI) calcd. for $C_{24}H_{13}Cl_2NO_2$+H$^+$: 418.0396; Found: 418.0396.

3.6. 2-Phenyl-6-(trifluoromethyl)-4-(4-(trifluoromethyl)phenyl)-1*H*-benzo[*f*]isoindole-1,3(2*H*)-dione (4f)

According to GP2 and after purification of the crude product by column chromatography (*n*-hexane/ethyl acetate 10:1) compound **4f** (157 mg, 65%) was obtained as a light brown solid, R_f (*n*-hexane:EtOAc, 5:1): 0.33, Mp 216 °C. ^{1}H NMR (300 MHz, CDCl$_3$): δ 7.33-7.53 (m, 5 H), 7.58 (d, J = 8.0 Hz, 2 H), 7.85 (d, J = 8.0 Hz, 2 H), 7.93 (dd, J = 8.6, 1.7 Hz, 1 H), 8.04 (s, 1 H), 8.31 (d, J = 8.5 Hz, 1 H), 8.61 (s, 1 H). ^{13}C NMR (75 MHz, CDCl$_3$): δ 123.6 (q, $^1J_{C-F}$ = 272.4 Hz, C_{quat}), 124.2 (q, $^1J_{C-F}$ = 272.4 Hz, C_{quat}), 125.0 (C_{quat}), 125.15, 125.21 (q, $^3J_{C-F}$ = 3.2 Hz), 125.5 (q, $^3J_{C-F}$ = 4.4 Hz), 125.68 (q, $^3J_{C-F}$ = 3.7 Hz), 126.7, 128.6, 129.2, 129.6 (C_{quat}), 130.5, 131.45 (q, $^2J_{C-F}$ = 32.8 Hz, C_{quat}), 131.47 (q, $^2J_{C-F}$ = 32.9 Hz, C_{quat}), 131.5 (C_{quat}), 131.8, 134.6 (C_{quat}), 137.1 (C_{quat}), 137.2 (C_{quat}), 139.9 (C_{quat}), 165.9 (C_{quat}), 166.1 (C_{quat}). IR (ATR): $\tilde{\nu}$ 3042 (w), 2961 (w), 2924 (w), 1775 (m), 1717 (s), 1595 (m), 1452 (m), 1406 (m), 1368 (s), 1323 (s), 1314 (s), 1287 (s), 1261 (m), 1171 (s), 1107 (s), 1088 (m), 1067 (s), 1020 (m), 966 (m), 953 (m), 910 (m), 899 (m), 853 (m), 837 (m), 822 (s), 797 (m), 768 (s), 731 (m), 694 (m), 683 (m), 638 (m), 619 (s); EI MS (70 eV): *m/z* (%) 486 (30), 485 (M^+, 100), 484 (29), 441 (11), 440 (31), 416 (10), 373 (12), 372 (42), 269 (16), 77 ($C_6H_5^+$, 44). HR MS (ESI) calcd. for $C_{26}H_{13}F_6NO_2$+H$^+$: 486.0923; Found: 486.0923.

3.7. 6-Methyl-2-phenyl-4-(4-tolyl)-1*H*-benzo[*f*]isoindole-1,3(2*H*)-dione (4g)

According to GP2 and after purification of the crude product by column chromatography (*n*-hexane/ethyl acetate 10:1) compound **4g** (181 mg, 48%) was obtained as a yellow solid, R_f (*n*-hexane:EtOAc, 10:1): 0.22, Mp 245 °C. ^{1}H NMR (300 MHz, CDCl$_3$): δ 2.49 (s, 6 H), 7.29-7.40

(m, 5 H), 7.42-7.48 (m, 4 H), 7.54 (dd, J = 8.3, 1.6 Hz, 1 H), 7.61-7.68 (m, 1 H), 8.02 (d, J = 8.3 Hz, 1 H), 8.43 (s, 1 H). ^{13}C NMR (151 MHz, $CDCl_3$): δ 21.6 (CH_3), 22.3 (CH_3), 123.6 (C_{quat}), 124.6, 126.78 (C_{quat}), 126.82, 127.9, 128.0, 129.0, 129.1, 129.9, 130.3, 131.4, 131.6 (C_{quat}), 132.1 (C_{quat}), 134.0 (C_{quat}), 136.2 (C_{quat}), 138.4 (C_{quat}), 139.8 (C_{quat}), 140.6 (C_{quat}), 166.7 (C_{quat}), 167.2 (C_{quat}). IR (ATR): $\tilde{\nu}$ 3084 (w), 3034 (w), 2918 (w), 1759 (m), 1712 (s), 1697 (m), 1589 (m), 1516 (m), 1490 (m), 1452 (m), 1415 (m), 1398 (m), 1354 (s), 1311 (m), 1292 (w), 1280 (w), 1255 (w), 1234 (w), 1213 (w), 1178 (m), 1149 (m), 1124 (m), 1099 (s), 1062 (m), 1039 (w), 1014 (w), 956 (m), 941 (w), 902 (s), 889 (w), 854 (m), 825 (s), 804 (m), 785 (w), 765 (s), 752 (s), 742 (m), 725(m), 711 (m), 690 (m), 663 (m). EI MS (70 eV): *m/z* (%) 378 (28), 377 (M^+, 100), 360 (29), 359 (54), 358 (12), 334 (24), 333 (95), 332 (35), 317 (14), 202 ($C_{16}H_{10}^+$, 19), 201 (12), 200 (13). Anal. calcd. for $C_{26}H_{19}NO_2$ (377.1): C 82.74, H 5.07, N 3.71; Found: C 82.85, H 5.17, N 3.65.

3.8. 2-(4-Fluorophenyl)-4-phenyl-1*H*-benzo[*f*]isoindole-1,3(2*H*)-dione (4h)

According to GP2 and after purification of the crude product by column chromatography (*n*-hexane/ethyl acetate 10:1) compound **4h** (201 mg, 55%) was obtained as a light yellow solid, R_f (*n*-hexane:EtOAc, 10:1): 0.21, Mp 220 °C. ^{1}H NMR (600 MHz, $CDCl_3$): δ 7.13-7.16 (m, 2 H), 7.42-7.45 (m, 4 H), 7.52-7.57 (m, 3 H), 7.64 (ddd, J = 8.4, 6.9, 1.4 Hz, 1 H), 7.73 (ddd, J = 8.2, 6.9, 1.3 Hz, 1 H), 7.85 (d, J = 8.4 Hz, 1 H), 8.14 (d, J = 8.1 Hz, 1 H), 8.50 (s, 1 H). ^{13}C NMR (151 MHz, $CDCl_3$); δ 116.0 (d, $^2J_{C\text{-}F}$ = 22.9 Hz), 123.3 (C_{quat}), 125.1, 127.4, 127.9 (d, $^4J_{C\text{-}F}$ = 3.2 Hz, C_{quat}), 128.4, 128.57, 128.63, 128.8 (d, $^3J_{C\text{-}F}$ = 9.2 Hz), 129.3, 129.5, 129.9, 130.6 (C_{quat}), 134.4 (C_{quat}), 135.8 (C_{quat}), 135.9 (C_{quat}), 141.2 (C_{quat}), 162.0 (d, $^1J_{C\text{-}F}$ = 247.8 Hz, C_{quat}), 166.5 (C_{quat}), 166.9 (C_{quat}). IR (ATR): $\tilde{\nu}$ 3047 (w), 2924 (w), 1767 (m), 1748 (m), 1713 (s), 1684 (m), 1593 (m), 1516 (w), 1491 (m), 1435 (m), 1402 (m), 1368 (s), 1356 (s), 1329 (m), 1296 (w), 1256 (m), 1203 (m), 1173 (w), 1112 (m), 1076 (m), 1066 (w), 999 (w), 945 (m), 921 (m), 908 (m), 896 (w), 871 (w), 769 (s), 750 (s), 732 (s), 700 (s), 686 (s), 657 (m), 638 (m). EI MS (70 eV): *m/z* (%) 368 (25), 367 (M^+, 100), 366 (42), 323 (16), 322 (50), 202 ($C_{16}H_{10}^+$, 22), 201 (33), 200 (19). Anal. calcd. for $C_{24}H_{14}FNO_2$ (367.1): C 78.46, H 3.84, N 3.84; Found: C 78.19, H 4.02, N 3.60.

3.9. 2-(4-Chlorophenyl)-4-phenyl-1*H*-benzo[*f*]isoindole-1,3(2*H*)-dione (4i)

According to GP2 and after purification of the crude product by column chromatography (*n*-hexane/ethyl acetate 10:1) compound **4i** (250 mg, 65%) was obtained as a yellow solid, R_f (*n*-hexane:EtOAc, 10:1): 0.15, Mp 180 °C (lit.:[6] 250 °C). ^{1}H NMR (300 MHz, $CDCl_3$): δ 7.39-7.47 (m, 6 H), 7.52-7.59 (m, 3 H), 7.59-7.68 (m, 1 H), 7.73 (ddd, J = 8.2, 6.9, 1.4 Hz, 1 H), 7.85 (d, J = 8.4 Hz, 1 H), 8.04 (dt, J = 7.3, 1.5 Hz, 1 H), 8.50 (s, 1 H). ^{13}C NMR (75 MHz, $CDCl_3$): δ 123.2

(C_{quat}), 124.9, 127.7 (C_{quat}), 128.2, 128.5, 128.6, 128.7, 129.4, 129.7, 130.4, 130.8 (C_{quat}), 134.2 (C_{quat}), 135.7 (C_{quat}), 141.1 (C_{quat}), 166.1 (C_{quat}), 166.5 (C_{quat}). IR (ATR): $\tilde{\nu}$ 3047 (w), 2924 (w), 1767 (m), 1748 (m), 1713 (s), 1684 (m), 1593 (m), 1516 (w), 1491 (m), 1435 (m), 1402 (m), 1368 (s), 1356 (s), 1329 (m), 1296 (w), 1256 (m), 1203 (m), 1173 (w), 1112 (m), 1076 (m), 1066 (w), 999 (w), 945 (m), 921 (m), 908 (m), 896 (w), 871 (w), 769 (s), 750 (s), 732 (s), 700 (s), 686 (s), 657 (m), 638 (m). EI MS (70 eV): *m/z* (%) 385 ($M(^{37}Cl)^+$, 32), 384 (25), 383 ($M(^{35}Cl)^+$, 100), 323 (16), 322 (50), 202 ($C_{16}H_{10}^+$, 22), 201 (33), 200 (19). Anal. calcd. for $C_{24}H_{14}ClNO_2$ (383.1): C 75.10, H 3.68, N 3.60; Found: C 74.89, H 3.62, N 3.60.

3.10. 2-(4-Iodophenyl)-4-phenyl-1*H*-benzo[*f*]isoindole-1,3(2*H*)-dione (4j)

According to GP2 and after purification of the crude product by column chromatography (*n*-hexane/ethyl acetate 10:1) compound **4j** (241 mg, 51%) was obtained as a yellow solid, R_f (*n*-hexane:EtOAc, 10:1): 0.22, Mp 250 °C. ^{1}H NMR (300 MHz, $CDCl_3$): δ 7.22-7.23 (m, 1 H), 7.25-7.26 (m, 1 H), 7.41-7.46 (m, 2 H), 7.53-7.58 (m, 2 H), 7.63 (ddd, *J* = 8.3, 6.9, 1.4 Hz, 1 H), 7.73 (ddd, *J* = 8.2, 7.0, 1.4 Hz, 1 H), 7.75-7.77 (m, 1 H), 7.79-7.80 (m, 1 H), 7.84-7.87 (m, 1 H)8.12-8.15 (m, 1 H), 8.50 (s, 1 H). ^{13}C NMR (75 MHz, $CDCl_3$): δ 93.1 (C_{quat}), 123.3 (C_{quat}), 125.1, 127.3 (C_{quat}), 128.3, 128.4, 128.79, 128.83, 129.4, 129.5, 129.9, 130.6, 131.8 (C_{quat}), 134.4 (C_{quat}), 135.8 (C_{quat}), 135.9 (C_{quat}), 138.1 (C_{quat}), 141.3 (C_{quat}), 166.1 (C_{quat}), 166.6 (C_{quat}). IR (ATR): $\tilde{\nu}$ 3047 (w), 2924 (w), 1767 (m), 1748 (m), 1713 (s), 1684 (m), 1593 (m), 1516 (w), 1491 (m), 1435 (m), 1402 (m), 1368 (s), 1356 (s), 1329 (m), 1296 (w), 1256 (m), 1203 (m), 1173 (w), 1112 (m), 1076 (m), 1066 (w), 999 (w), 945 (m), 921 (m), 908 (m), 896 (w), 871 (w), 769 (s), 750 (s), 732 (s), 700 (s), 686 (s), 657 (m), 638 (m). EI MS (70 eV): *m/z* (%) 476 (22), 475 (M^+, 100), 474 (30), 430 (29), 304 (27), 303 (18), 227 (12), 202 ($C_{16}H_{10}^+$, 31), 201 (34), 200 (25), 173 (15), 159 (21), 145 (12). Anal. calcd. for $C_{24}H_{14}INO_2$ (475.3): C 60.65, H 2.97, N 2.95; Found: C 60.38, H 3.15, N 2.87.

3.11. Ethyl 4-(1,3-dioxo-4-phenyl-1,3-dihydro-2*H*-benzo[*f*]isoindol-2-yl)benzoate (4k)

According to GP2 and after purification of the crude product by column chromatography (*n*-hexane/ethyl acetate 10:1) compound **4k** (240 mg, 57%) was obtained as a yellow solid, R_f (*n*-hexane:EtOAc, 10:1): 0.15, Mp 185 °C. ^{1}H NMR (300 MHz, $CDCl_3$): δ 1.40 (t, *J* = 7.1 Hz, 3 H), 4.39 (q, *J* = 7.1 Hz, 2 H), 7.42-7.47 (m, 2 H), 7.55-7.64 (m, 6 H), 7.73 (ddd, *J* = 8.2, 6.9, 1.3 Hz, 1 H), 7.84-7.87 (m, 1 H), 8.11-8.15 (m, 3 H), 8.51 (s, 1 H). ^{13}C NMR (151 MHz, $CDCl_3$): δ 14.3 (CH_3), 61.2 (CH_2), 123.1 (C_{quat}), 125.0, 126.1, 127.1 (C_{quat}), 128.2, 128.6, 128.7, 129.3, 129.8, 130.2, 130.4, 131.8 (C_{quat}), 134.2 (C_{quat}), 135.7 (C_{quat}), 135.8 (C_{quat}), 135.9 (C_{quat}), 141.2 (C_{quat}), 165.92 (C_{quat}), 165.95 (C_{quat}), 166.4 (C_{quat}). IR (ATR): $\tilde{\nu}$ 2980 (w), 2960 (w), 2929 (w), 1768 (m),

1718 (s), 1701 (s), 1697 (s), 1680 (m), 1600 (m), 1593 (m), 1508 (m), 1465 (w), 1448 (m), 1409 (m), 1400 (m), 1352 (s), 1330 (m), 1307 (m), 1271 (s), 1203 (m), 1172 (m), 1105 (s), 1091 (s), 1072 (m), 1018 (m), 1002 (m), 943 (m), 900 (m), 875 (w), 858 (m), 833 (w), 810 (m), 792 (m), 763 (s), 738 (s), 694 (s), 682 (m), 640 (s), 630 (m), 600 (m). EI MS (70 eV): *m/z* 422 (26), 421 (M^+, 100), 420 (25), 393 (23), 392 (21), 377 (28), 376 (89), 349 (14), 348 (39), 304 (22), 291 811), 227 (11), 202 ($C_{16}H_{10}^+$, 36), 201 (34), 200 (24), 187 (17), 151 (10), 145 (10), 134 (15). Anal. calcd. for $C_{27}H_{19}NO_4$ (421.1): C 76.95, H 4.54, N 3.32; Found: C 76.88, H 4.66, N 3.24.

3.12. 2-(3,5-Dimethylphenyl)-4-phenyl-1*H*-benzo[*f*]isoindole-1,3(2*H*)-dione (4l)

According to GP2 and after purification of the crude product by column chromatography (*n*-hexane/ethyl acetate 10:1) compound **4l** (260 mg, 69%) was obtained as a yellow solid, R_f (*n*-hexane:EtOAc, 10:1): 0.22, Mp 210 °C. ^{1}H NMR (300 MHz, $CDCl_3$): δ 2.33 (s, 6 H), 7.00-7.03 (m, 3 H), 7.42-7.46 (m, 2 H), 7.50-7.57 (m, 3 H), 7.59-7.65 (m, 1 H), 7.69-7.74 (m, 1 H), 7.86 (d, *J* = 8.4 Hz, 1 H), 8.03 (dd, *J* = 8.0 Hz, 1 H), 8.49 (s, 1 H). ^{13}C NMR (75 MHz, $CDCl_3$): δ 21.4 (CH_3), 123.6 (C_{quat}), 124.7, 124.8, 127.7 (C_{quat}), 128.3, 128.7, 128.8, 129.2, 129.3, 129.8, 130.0, 130.1,130.5, 131.6 (C_{quat}), 134.5 (C_{quat}), 135.79 (C_{quat}), 135.83 (C_{quat}), 138.8 (C_{quat}), 140.9 (C_{quat}), 166.7 (C_{quat}), 167.2 (C_{quat}). IR (ATR): $\tilde{\nu}$ 3057 (w), 2958 (w), 2914 (w), 2848 (w), 1764 (m), 1705 (s), 1693 (m), 1597 (m), 1550 (w), 1516 (m), 1492 (w), 1465 (m), 1440 (m), 1406 (m), 1342 (s), 1327 (m), 1265 (m), 1228 (w), 1217 (w), 1188 (w), 1139 (w), 1089 (m), 1064 (m), 954 (w), 929 (w), 896 (m), 852 (m), 817 (w), 779 (m), 763 (s), 742 (w), 698 (m), 636 (m); EI MS (70 eV): *m/z* (%) 378 (25), 377 (M^+, 100), 376 (53), 333 (19), 332 (58), 318 (19), 202 ($C_{16}H_{10}^+$, 19), 201 (21), 200 (18). Anal. calcd. for $C_{26}H_{19}NO_2$ (377.1): C 82.74, H 5.07, N 3.71; Found: C 82.60, H 5.07, N 3.59.

3.13. 2-(2,6-Dimethylphenyl)-4-phenyl-1*H*-benzo[*f*]isoindole-1,3(2*H*)-dione (4m)

According to GP2 and after purification of the crude product by column chromatography (*n*-hexane/ethyl acetate 10:1) compound **4m** (211 mg, 56%) was obtained as a yellow solid, R_f (*n*-hexane:EtOAc, 10:1): 0.22, Mp 205 °C. ^{1}H NMR (300 MHz, $CDCl_3$): δ 2.23 (s, 6 H), 7.19-7.21 (m, 2 H), 7.28-7.32 (m, 1 H), 7.51-7.54 (m, 2 H), 7.57-7.64 (m, 3 H), 7.67-7.72 (m, 1 H), 7.78 (ddd, *J* = 8.2, 7.0, 1.3 Hz, 1 H), 7.94-7.97 (m, 1 H), 8.19-8.22 (m, 1 H), 8.51 (s, 1 H). ^{13}C NMR (75 MHz, $CDCl_3$): δ 18.3 (CH_3), 123.7 (C_{quat}), 124.9, 127.9 (C_{quat}), 128.3, 128.4, 128.7, 128.8, 129.2, 129.4, 130.2, 130.3 (C_{quat}), 130.5, 134.3 (C_{quat}), 135.7 (C_{quat}), 135.8 (C_{quat}), 136.7 (C_{quat}), 141.0 (C_{quat}), 166.3 (C_{quat}), 166.9 (C_{quat}). IR (ATR): $\tilde{\nu}$ 3061 (w), 3012 (w), 2958 (w), 2910 (w), 1759 (m), 1708 (s), 1678 (m), 1654 (w), 1625 (w), 1604 (m), 1516 (m), 1494 (w), 1473 (m), 1442 (m), 1400 (m), 1359 (s), 1332 (m), 1298 (w), 1274 (w), 1259 (m), 1242 (w), 1226 (w), 1199 (m),

1180 (w), 1118 (s), 1074 (m), 1029 (m), 945 (m), 898 (w), 867 (w), 839 (w), 798 (m), 763 (s), 744 (m), 715 (m), 696 (m), 661 (m), 638 (m), 611 (m). EI MS (70 eV): *m/z* (%) 378 (28), 377 (M^+, 100), 360 (29), 359 (54), 358 (12), 334 (24), 333 (95), 332 (35), 317 (14), 202 (19), 201 (12), 200 (13). Anal. calcd. for $C_{26}H_{19}NO_2$ (377.1): C 82.74, H 5.07, N 3.71; Found: C 82.49, H 5.06, N 3.57.

3.14. 2-(3,5-Dimethoxyphenyl)-4-phenyl-1*H*-benzo[*f*]isoindole-1,3(2*H*)-dione (4n)

According to GP2 and after purification of the crude product by column chromatography (*n*-hexane/ethyl acetate 2:5) compound **4n** (261 mg, 64%) was obtained as a yellow solid, R_f (*n*-hexane:EtOAc, 2:5): 0.14, Mp 195 °C. ^{1}H NMR (300 MHz, $CDCl_3$): δ 3.78 (s, 6 H), 6.47 (t, *J* = 2.3 Hz, 1 H), 6.59 (d, *J* = 2.3 Hz, 2 H), 7.42-7.47 (m, 2 H), 7.51-7.57 (m, 3 H), 7.60-7.65 (m, 1 H), 7.69-7.75 (m, 1 H), 7.84-7.87 (m, 1 H), 8.12-8.15 (m, 1 H), 8.49 (s, 1 H). ^{13}C NMR (75 MHz, $CDCl_3$): δ 55.7 (CH_3), 101.0, 105.3, 123.4 (C_{quat}), 124.9, 127.6 (C_{quat}), 128.3, 128.7, 128.8, 129.2, 129.4, 130.0, 130.5, 133.4 (C_{quat}), 134.5 (C_{quat}), 135.77 (C_{quat}), 135.82 (C_{quat}), 141.0 (C_{quat}), 160.9 (C_{quat}), 166.3 (C_{quat}), 166.9 (C_{quat}). IR (ATR): $\tilde{\nu}$ 3057 (w), 2910 (w), 2852 (w), 2721 (w), 1770 (m), 1761 (w), 1716 (s), 1699 (m), 1606 (w), 1591 (w), 1516 (w), 1494 (s), 1440 (m), 1408 (m), 1398 (m), 1359 (s), 1330 (m), 1298 (w), 1280 (w), 1203 (m), 1178 (w), 1111 (m), 1083 (s), 1072 (m), 1016 (m), 1001 (w), 991 (w), 943 (m), 916 (m), 896 (m), 869 (w), 835 (s), 810 (m), 785 (s), 775 (m), 763 (s), 740 (s), 696 (s), 642 (s), 628 (m). EI MS (70 eV): *m/z* (%) 410 (29), 409 (M^+, 100), 408 (35), 380 (31), 364 (16), 202 ($C_{16}H_{10}^+$, 23), 201 (20), 200 (17), 177 (12), 129 (18). Anal. calcd. for $C_{26}H_{19}NO_4$ (409.1): C 76.27, H 4.68, N 3.42; Found: C 76.04, H 4.82, N 3.35.

3.15. 2-Benzyl-4-phenyl-1*H*-benzo[*f*]isoindole-1,3(2*H*)-dione (4o)

According to GP2 and after purification of the crude product by column chromatography (*n*-hexane/ethyl acetate 10:1) compound **4o** (150 mg, 41%) was obtained as a yellow solid, R_f (*n*-hexane:EtOAc, 10:1): 0.21, Mp 210 °C. ^{1}H NMR (300 MHz, $CDCl_3$): δ 4.83 (s, 2 H), 7.24-7.32 (m, 3 H)[2], 7.38-7.46 (m, 4 H), 7.53-7.61 (m, 4 H), 7.67 (ddd, *J* = 8.2, 6.9, 1.3 Hz, 1 H), 7.77-7.81 (m, 1 H), 8.06-8.08 (m, 1 H), 8.37 (s, 1 H). ^{13}C NMR (75 MHz, $CDCl_3$) δ 41.9 (CH_2), 123.9 (C_{quat}), 124.4, 127.9, 128.0 (C_{quat}), 128.3, 128.69, 128.74, 129.01, 129.04, 129.2, 130.0, 130.5, 134.6 (C_{quat}), 135.55 (C_{quat}), 135.62 (C_{quat}), 136.6 (C_{quat}), 140.4 (C_{quat}), 167.1 (C_{quat}), 167.8 (C_{quat}). IR (ATR): $\tilde{\nu}$ 3446 (w), 3032 (w), 2962 (w), 2854 (w), 1762 (m), 1712 (s), 1697 (m), 1693 (m), 1678 (m), 1629 (m), 1600 (w), 1556 (w), 1514 (m), 1492 (m), 1433 (m), 1406 (m), 1379 (m), 1340 (m), 1325 (m), 1292 (w), 1261 (m), 1207 (w), 1143 (m), 1118 (m), 1074 (m), 1058 (m), 1028 (m),

---

[2] Signal is overlapped by the signal of $CDCl_3$ (δ 7.26).

1000 (m), 950 (m), 933 (w), 921 (w), 900 (w), 860 (m), 804 (m), 758 (s), 752 (s), 742 (m), 700 (s), 669 (m), 653 (m), 626 (s). EI MS (70 eV): *m/z* (%) 364 (27), 363 (M^+, 100), 362 (14), 319 (18), 318 (24), 317 (12), 260 (13), 259 (65), 258 (11), 256 (11), 231 (20), 230 (20), 203 (24), 202 ($C_{16}H_{10}^+$, 79), 201 (27), 200 (26), 149 (22), 105 (76), 101 (11), 91 (15), 77 (25). HR MS (ESI) calcd. for $C_{25}H_{17}NO_2+H^+$: 364.1332; Found: 364.1332.

3.16. 4-Phenyl-2-(prop-2-yn-1-yl)-1*H*-benzo[*f*]isoindole-1,3(2*H*)-dione (4p)

According to GP2 and after purification of the crude product by column chromatography (*n*-hexane/ethyl acetate 10:1) compound **4p** (81 mg (0.41 mmol, 26%) was obtained as a yellow solid, R_f (*n*-hexane:EtOAc, 10:1): 0.20, Mp: 242 °C. ^{1}H NMR (300 MHz, $CDCl_3$): δ 2.19 (t, *J* = 2.5 Hz, 1 H), 4.44 (d, *J* = 2.5 Hz, 2 H), 7.38-7.44 (m, 2 H), 7.53-7.64 (m, 4 H), 7.70 (ddd, *J* = 8.2, 6.9, 1.3 Hz, 1 H), 7.80-7.85 (m, 1 H), 8.08-8.13 (m, 1 H), 8.42 (s, 1 H). ^{13}C NMR (151 MHz, $CDCl_3$) δ 27.2 (CH_2), 71.4 (CH), 77.4 (C_{quat}), 123.7 (C_{quat}), 124.8, 127.6 (C_{quat}), 128.3 128.7, 128.8, 129.2, 129.4, 130.0, 130.5, 134.8 (C_{quat}), 135.60 (C_{quat}), 135.63 (C_{quat}), 140.9 (Cq_{uat}), 166.2 (C_{quat}), 166.7 (C_{quat}). IR (ATR): $\tilde{\nu}$ 3458 (w), 3288 (m), 3084 (w), 3049 (w), 2966 (m), 2926 (w), 2852 (w), 2752 (w), 2698 (w), 2507 (w), 2436 (w), 2378 (w), 2214 (w), 1830 (w), 1759 (m), 1712 (s), 1681 (m), 1666 (m), 1624 (m), 1604 (m), 1593 (m), 1573 (m), 1554 (m), 1516 (m), 1490 (m), 1483 (m), 1398 (m), 1381 (m), 1367 (m), 1346 (m), 1315 (m), 1276 (w), 1259 (w), 1238 (w), 1226 (w), 1215 (w), 1188 (m), 1170 (m), 1126 (s), 1070 (m), 1031 (m), 1016 (w), 1001 (w), 989 (w), 958 (m), 935 (m), 902 (w), 866 (m), 802 (m), 761 (s), 748 (s), 734 (m), 698 (s), 661 (s), 630 (s), 609 (m). EI MS (70 eV): *m/z* (%) 312 (25), 311 (M^+, 100), 283 (13), 282 (41), 274 (14), 255 (10), 254 (29), 229 (12), 228 (13), 227 (16), 226 (12), 200 (11), 203 (11), 202 ($C_{16}H_{10}^+$, 64), 201 (34), 200 (33), 101 (11), 100 (19). HR MS (ESI) calcd. for $C_{21}H_{13}NO_2+H^+$: 312.1018; Found: 312.1019.

3.17. 2-*n*-Hexyl-4-phenyl-1*H*-benzo[*f*]isoindole-1,3(2*H*)-dione (4q)

According to GP2 and after purification of the crude product by column chromatography (*n*-hexane/ethyl acetate 10:1) compound **4q** (190 mg, 53%) was obtained as a yellow solid, R_f (*n*-hexane:EtOAc, 10:1): 0.23, Mp 127-128 °C. ^{1}H NMR (600 MHz, $CDCl_3$): δ 0.80-0.90 (m, 3 H), 1.23-1.36 (m, 6 H), 1.63-1.67 (m, 2 H), 3.60-3.67 (m, 2 H), 7.37-7.46 (m, 2 H), 7.48-7.63 (m, 4 H), 7.68 (ddd, *J* = 8.1, 7.0, 1.1 Hz, 1 H), 7.81 (d, *J* = 8.5 Hz, 1 H), 8.08 (d, *J* = 8.1 Hz, 1 H), 8.36 (s, 1 H). ^{13}C NMR (151 MHz, $CDCl_3$): δ 14.1 (CH_3), 22.6 (CH_2), 26.8 (CH_2), 28.6 (CH_2), 31.5 (CH_2), 38.4 (CH_2), 124.0 (C_{quat}), 124.2, 128.1 (C_{quat}), 128.3, 128.6, 128.7, 129.0, 129.1, 130.0, 130.4, 134.6 (C_{quat}), 135.55 (C_{quat}), 135.57 (C_{quat}), 140.1 (C_{quat}), 167.6 (C_{quat}), 168.1 (C_{quat}). IR (ATR): $\tilde{\nu}$ 3059 (w), 3022 (w), 2951 (w), 2922 (m), 2856 (w), 1759 (s), 1718 (m), 1697 (s), 1681

(m), 1608 (w), 1517 (m), 1494 (w), 1436 (m), 1398 (m), 1365 (s), 1342 (m), 1328 (m),1315 (w), 1301 (w), 1261 (w), 1226 (m),1188 (w), 1170 (w), 1147 (w), 1099 (s), 1076 (w), 1051 (m), 1031 (m), 1016 (w), 979 (m), 902 (m), 796 (m), 763 (s), 738 (m), 721 (m), 702 (s), 638 (m), 623 (m). EI MS (70 eV): *m/z* (%) 358 (12), 357 (M^+, 47), 328 (12), 300 (15), 288 (20), 287 ($C_{19}H_{13}NO_2^+$, 100), 286 (61), 272 (11), 259 (20), 256 (15), 231 (12), 230 (14), 229 (13), 228 (11), 227 (11), 203 (13), 202 (54), 201 (23), 200 (17). Anal. calcd. for $C_{24}H_{23}NO_2$ (357.2): C 80.64, H 6.49, N 3.92; Found: C 80.83, H 6.47, N 3.87.

3.18. 2-*n*-Butyl-4-phenyl-1*H*-benzo[*f*]isoindole-1,3(2*H*)-dione (4r)

According to GP2 and after purification of the crude product by column chromatography (*n*-hexane/ethyl acetate 10:1) compound **4r** (200 mg, 61%) was obtained as a yellow solid, R_f (*n*-hexane:EtOAc, 10:1): 0.22, Mp 143 °C (lit.:[7] 138 °C). ^{1}H NMR (300 MHz, $CDCl_3$): δ 0.92 (t, *J* = 7.3 Hz, 3 H), 1.26-1.42 (m, 2 H), 1.58-1.78 (m, 2 H), 3.59-3.72 (m, 2 H), 7.36-7.45 (m, 2 H), 7.51-7.62 (m, 4 H), 7.67 (ddd, *J* = 8.2, 6.9, 1.3 Hz, 1 H), 7.77-7.85 (m, 1 H), 8.04-8.12 (m, 1 H), 8.36 (s, 1 H); ^{13}C NMR (75 MHz, $CDCl_3$): δ 13.8 (CH_3), 20.3 (CH_2), 30.6 (CH_2), 38.1 (CH_2), 124.0 (C_{quat}), 124.1, 128.0 (C_{quat}), 128.3, 128.59, 128.63, 128.9, 129.1, 130.0, 130.4, 134.6 (C_{quat}), 135.52 (C_{quat}), 135.53 (C_{quat}), 140.1 (C_{quat}), 167.6 (C_{quat}), 168.0 (C_{quat}). IR (ATR): $\tilde{\nu}$ 3042 (w), 3055 (w), 2956 (m), 2927 (m), 2870 (w), 2860 (w), 2721 (w), 1759 (s), 1685 (s), 1647 (m), 1627 (w), 1610 (m), 1593 (w), 1575 (w), 1517 (m), 1496 (m), 1438 (m), 1398 (m),1384 (m), 1354 (s), 1334 (m), 1315 (m), 1301 (w), 1284 (w), 1255 (w), 1226 (w), 1168 (m), 1147 (w), 1093 (s), 1074 (m), 1029 (m), 972 (m), 950 (m), 923 (w), 902 (m), 875 (w), 831 (m), 788 (m), 761 (s), 731 (m), 700 (s), 667 (m), 655 (m), 638 (s), 611 (m). EI MS (70 eV): *m/z* (%) 330 (11), 329 (M^+, 48), 300 (12), 288 (18), 287 (100), 286 (59), 272 (13), 259 (19), 256 (10), 231 (11), 230 (14), 229 (13), 228 (10), 227 (10), 203 (14), 202 ($C_{16}H_{10}^+$, 58), 201 (25), 200 (22), 181 (11), 167 (12), 165 (26), 149 (14), 129 (30), 102 (25), 101 (16). Anal. calcd. for $C_{22}H_{19}NO_2$ (329.1): C 80.22, H 5.81, N 4.25; Found: C 80.00, H 5.79, N 4.19.

3.19. 2-(2,6-Dimethylphenyl)-6-methoxy-4-(4-methoxyphenyl)-1*H*-benzo[*f*]isoindole-1,3(2*H*)-dione (4s)

According to GP2 and after purification of the crude product by column chromatography (*n*-hexane/ethyl acetate 10:1) compound **4s** (250 mg, 57%) was obtained as a yellow solid, R_f (*n*-hexane:EtOAc, 10:1): 0.13, Mp 252 °C. ^{1}H NMR (600 MHz, $CDCl_3$): δ 2.17 (s, 6 H), 3.79 (s, 3 H), 3.89 (s, 3 H), 7.07 (d, *J* = 8.3 Hz, 2 H), 7.13 (d, *J* = 7.6 Hz, 2 H), 7.21 (t, *J* = 7.6 Hz, 2 H), 7.34-7.35 (m, 1 H), 7.41 (d, *J* = 8.3 Hz, 2 H), 8.02 (d, *J* = 8.9 Hz, 1 H), 8.40 (s, 1 H). ^{13}C NMR (151 MHz, $CDCl_3$): δ 18.3 (CH_3), 55.4 (CH_3), 55.6 (CH_3), 107.7, 113.9, 121.2, 124.38 (C_{quat}),

124.43, 125.8 (C_{quat}), 126.5 (C_{quat}), 128.4, 129.3, 130.4 (C_{quat}), 131.0 (C_{quat}), 131.4, 132.0, 136.8 (C_{quat}), 137.9 (C_{quat}), 139.3 (C_{quat}), 159.9 (C_{quat}), 160.4 (C_{quat}), 166.7 (C_{quat}), 167.2 (C_{quat}). IR (ATR): $\tilde{\nu}$ 3066 (w), 3018 (w), 2968 (w), 2941 (w), 2922 (w), 2848 (w), 1766 (m), 1708 (s), 1680 (m), 1606 (m), 1575 (w), 1508 (m), 1467 (m), 1452 (m), 1433 (m), 1404 (m), 1359 (s), 1321 (m), 1300 (m), 1286 (m), 1242 (s), 1230 (m), 1184 (m), 1165 (m), 1126 (s), 1114 (m), 1082 (w), 1022 (m), 977 (m), 958 (w), 939 (w), 906 (m), 856 (m), 833 (s), 817 (w), 773 (s), 742 (m), 725 (w), 694 (m), 626 (m);. EI MS (70 eV): *m/z* (%) 438 (31), 437 (M^+, 100), 420 (32), 419 (65), 394 (14), 393 (52), 392 (22), 378 (25), 363 (13), 362 (14), 176 (20), 159 (15) 152 (15). Anal. calcd. for $C_{26}H_{23}NO_4$ (437.2): C 76.87, H 5.30, N 3.20; Found: C 77.15, H 5.30, N 3.08.

3.20. Synthesis of (*E*)-2,9-diphenyl-3-(phenylimino)-2,3-dihydro-1*H*-benzo[*f*]isoindol-1-one (5)

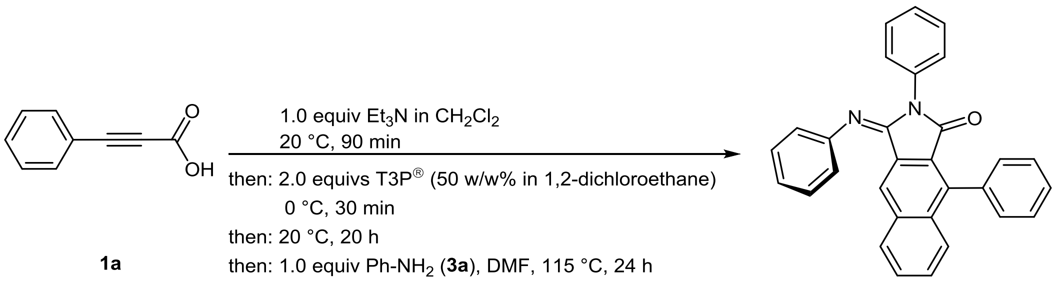

1a

5

In a Schlenk tube phenylpropiolic acid (**1a**) (292 mg, 2.00 mmol) were dissolved in dichloromethane (3.50 mL) under a nitrogen atmosphere. Then dry triethylamine (202 mg, 2.00 mmol) were added to the solution. The solution was stirred at room temperature for 90 min and then cooled to 0 °C with an ice bath. After 10 min the dropwise addition of T3P® (2.55 g, 4.01 mmol in 1,2-dichloroethane) was started. After complete addition of T3P® the reaction mixture was stirred at 0 °C for 30 min and then allowed to come to room temp and stirred for 20 h. Then DMF (4.00 mL) and aniline (**3a**) (186 mg, 2.00 mmol) were added to the reaction mixture. The reaction mixture was heated to 115 °C for 20 h. After cooling to room temp the reaction mixture was diluted with dichloromethane (20 mL) and washed with saturated solutions of ammonium chloride and sodium bicarbonate and with deionized water. The combined organic layers were dried (anhydrous magnesium sulfate), the solvents removed in vacuo and the residue was adsorbed on Celite®. After purification of the crude product by column chromatography on silica gel (*n*-hexane/ethylacetate, 20:1) compound **5** (63.2 mg, 15%) were obtained as a yellow solid, R_f (*n*-hexane:EtOAc, 5:1): 0.44, Mp 222 °C (lit.:[6] no data).

^{1}H NMR (600 MHz, $CDCl_3$): δ 7.09 (dd, J = 7.9, 1.3 Hz, 2 H), 7.18 (s, 1 H), 7.26-7.31 m, 1 H), 7.33-7.38 (m, 1 H), 7.40-7.42 (m, 2 H), 7.46 (d, J = 8.0 Hz, 2 H), 7.48 (d, J = 8.0 Hz, 2 H), 7.50-7.58 (m, 8 H), 7.62 (dd, J = 8.0, 1.6 Hz, 1 H), 7.76 (d, J = 8.2 Hz, 1 H). ^{13}C NMR (151 MHz, $CDCl_3$): δ 120.0, 124.1, 124.4 (C_{quat}), 125.2 (C_{quat}), 127.0, 128.0, 128.1, 128.16, 128.23, 128.3, 128.4, 128.7, 128.8, 129.6, 130.0, 130.3, 133.6 (C_{quat}), 134.4 (C_{quat}), 134.9 (C_{quat}), 135.0 (C_{quat}), 140.4 (C_{quat}), 149.5 (C_{quat}), 150.9 (C_{quat}), 166.3 (C_{quat}). IR (ATR): $\tilde{\nu}$ 3040 (w), 3028 (w), 2961 (w), 1724 (m), 1659 (s), 1618 (w), 1589 (m), 1490 (s), 1483 (m), 1420 (w), 1400 (w), 1375 (s), 1369 (s), 1335 (m), 1288 (w), 1261 (m), 1206 (m), 1144 (s), 1103 (m), 1072 (m), 1024 (m), 1001 (w), 947 (m), 899 (m), 839 (m), 806 (m), 777 (m), 756 (s), 745 (s), 735 (s), 698 (s), 690 (s), 646 (m), 637 (m), 611 (m); EI MS (70 eV): *m/z* (%) 425 (21), 424 (M^+, 78), 423 (100), 355 (12), 304 (12), 221 (13), 212 (15), 201 ($C_6H_9^+$, 12), 147 (10), 73 (10). HR MS (ESI) calcd. for $C_{30}H_{20}NO_2+H^+$ 425.1648; Found: 425.1649.

3.21. Synthesis of 6-Phenyl-12*H*-benzo[*f*]benzo[4,5]imidazo[2,1-*a*]isoindol-12-one (6)

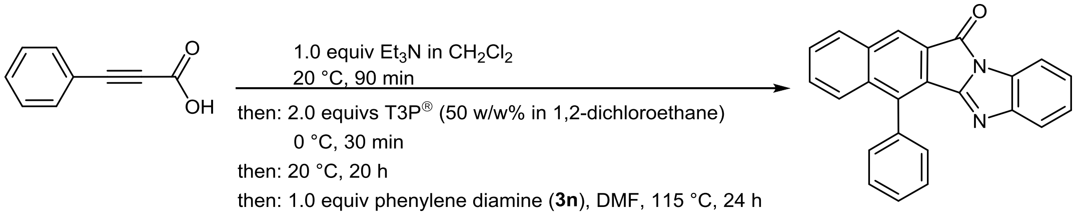

1a **6**

In a Schlenk tube phenylpropiolic acid (**1a**) (292 mg, 2.00 mmol) was dissolved in dichloromethane (4.00 mL) under a nitrogen atmosphere. Then dry triethylamine (202 mg, 2.00 mmol) was added to the solution. The solution was stirred at room temperature for 90 min and then cooled to 0 °C with an ice bath. After 10 min the dropwise addition of T3P® (2.56 g, 4.01 mmol in 1,2-dichloroethane) was started. After complete addition of T3P® the reaction mixture was stirred at 0 °C for 30 min and then allowed to come to room temp and stirred for 20 h. Then DMF (4.00 mL) and *ortho*-phenylenediamine (**3n**) (217 mg, 2.01 mmol) were added to the reaction mixture. The reaction mixture was heated to 115 °C for 20 h. After cooling to room temp the reaction mixture was diluted with dichloromethane (20 mL) and washed with saturated solutions of ammonium chloride and sodium bicarbonate and with deionized water. The combined organic layers were dried (anhydrous magnesium sulfate), the solvents removed in vacuo and the residue was adsorbed on Celite®. After purification of the crude product by column chromatography on silica gel (*n*-hexane/ethylacetate, 4:1) compound **6** (23.2 mg, 7%) was obtained as a yellow solid, R_f (*n*-hexane:EtOAc, 2:1): 0.69, Mp > 250 °C.

^{1}H NMR (300 MHz, CD_2Cl_2): δ 7.24-7.29 (m, 1 H), 7.32-7.37 (m, 1 H), 7.53-7.69 (m, 8 H), 7.73-7.76 (m, 1 H), 7.85 (dd, *J* = 7.4, 1.2 Hz, 1 H), 8.06 (dd, *J* = 7.6, 1.7 Hz, 1 H), 8.40 (s, 1 H). ^{13}C NMR (75 MHz, CD_2Cl_2): δ 113.2, 121.7, 124.9 (C_{quat}), 125.7, 126.5, 127.7, 128.5, 128.9, 129.0, 129.1, 130.0 (C_{quat}), 130.2, 130.9, 131.1, 132.0 (C_{quat}), 134.9 (C_{quat}), 135.8 (C_{quat}), 136.3 (C_{quat}), 137.9 (C_{quat}), 150.1 (C_{quat}), 156.9 (C_{quat}), 161.4 (C_{quat}). ^{1}H NMR (600 MHz, $CDCl_3$): δ 7.20-7.26 (m, 1 H), 7.28-7.34 (m, 1 H), 7.57-7.65 (m, 8 H), 7.78 (ddd, *J* = 7.8, 1.9, 0.8 Hz, 1 H), 7.85-7.88 (m, 1 H), 8.01-8.04 (m, 1 H), 8.40 (s, 1 H). ^{13}C NMR (151 MHz, $CDCl_3$): δ 112.9, 121.9, 124.4 (C_{quat}), 125.3, 126.1, 127.4, 128.2, 128.5, 128.8, 129.0, 129.5 (C_{quat}), 129.8, 130.4, 130.8, 131.5 (C_{quat}), 134.4 (C_{quat}), 135.0 (C_{quat}), 135.9 (C_{quat}), 137.7 (C_{quat}), 149.6 (C_{quat}), 149.6 (C_{quat}), 156.3 (C_{quat}), 161.1 (C_{quat}). IR (ATR): $\tilde{\nu}$ 3061 (w), 2963 (w), 2926 (w), 2855 (w), 1746 (s), 1638 (w), 1618 (m), 1609 (w), 1555 (m), 1514 (w), 1479 (m), 1443 (m), 1422 (m), 1375 (m), 1358 (m), 1335 (m), 1304 (m), 1288 (m), 1260 (s), 1215 (w), 1163 (m), 1142 (m), 1132 (m), 1088 (s), 1072 (s), 1016 (s), 957 (m), 910 (m), 862 (w), 797 (s), 781 (s), 750 (s), 741 (s), 704 (s), 696 (s), 652 (m), 638 (m). EI MS (70 eV): *m/z* (%) 347 (16), 346 (M^+, 74), 345 (100), 317 (14), 316 (17), 159 (25), 158 (27), 145 (11).

4. NMR spectra of compounds 2, 4, 5, and 6

4.1. 4-Phenylnaphtho[2,3-*c*]furan-1,3-dione (2a)

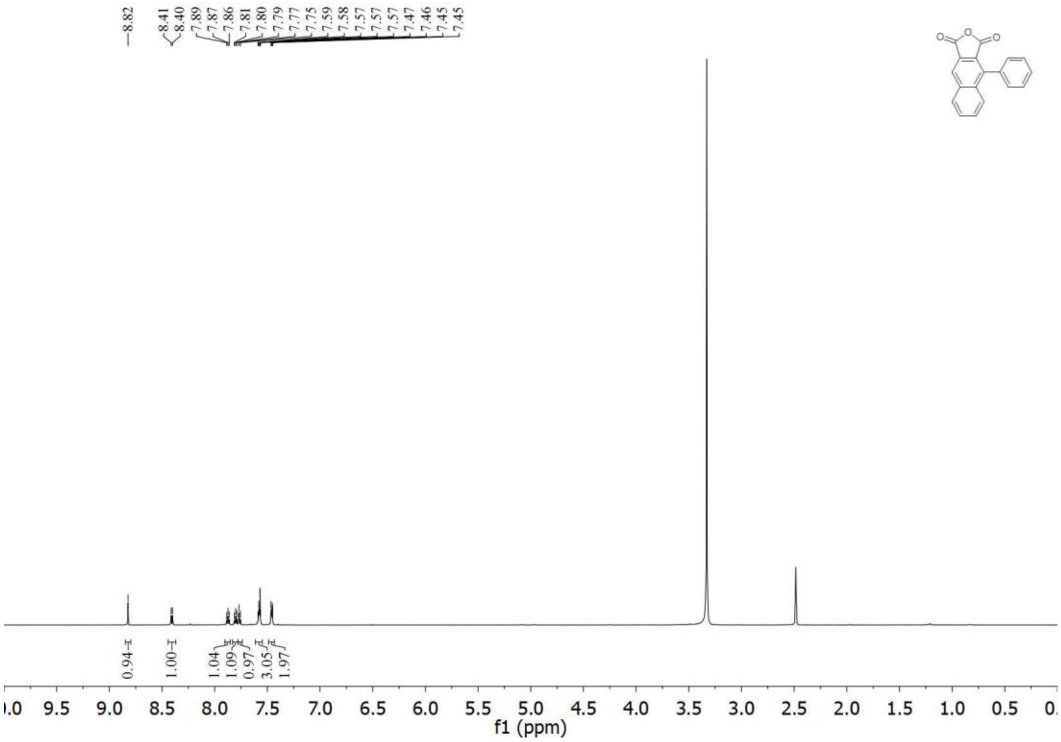

600 MHz ^{1}H NMR spectrum of compound **2a** recorded in $(CD_3)_2SO$ at T = 298 K.

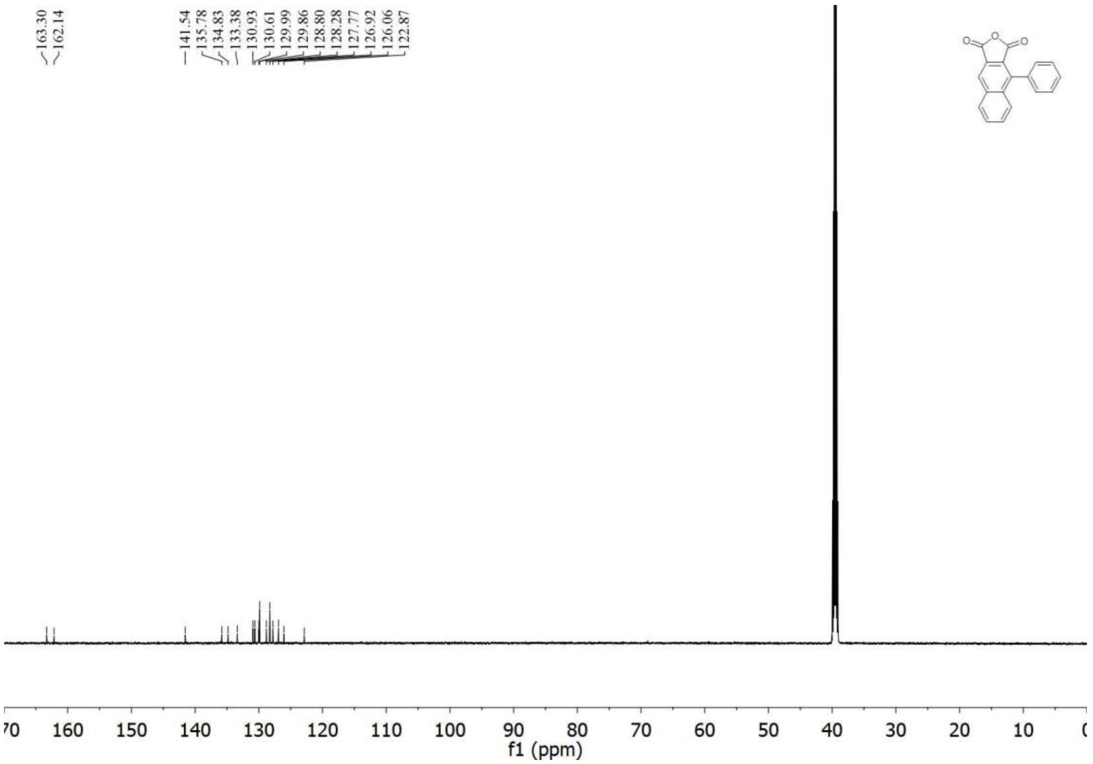

151 MHz ^{13}C NMR spectrum of compound **2a** recorded in $(CD_3)_2SO$ at T = 298 K.

4.2. 6-Methoxy-4-(4-methoxyphenyl)naphtho[2,3-*c*]furan-1,3-dione (2b)

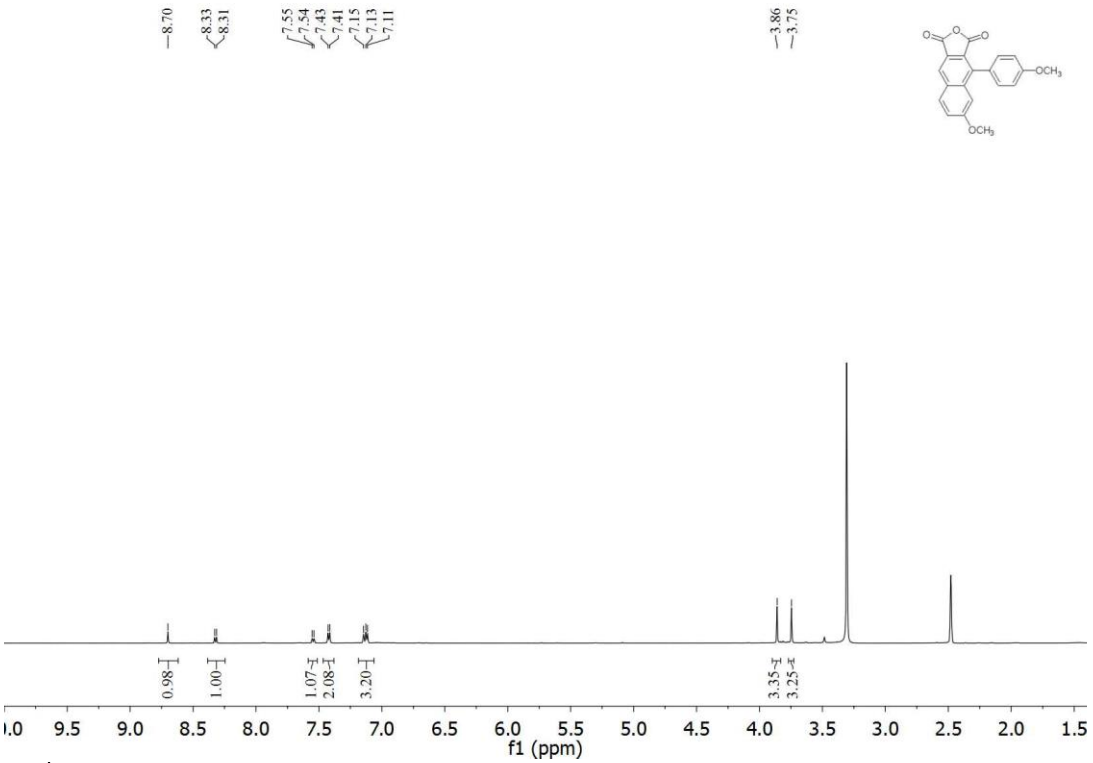

600 MHz ^{1}H NMR spectrum of compound **2b** recorded in $(CD_3)_2SO$ at T = 298 K.

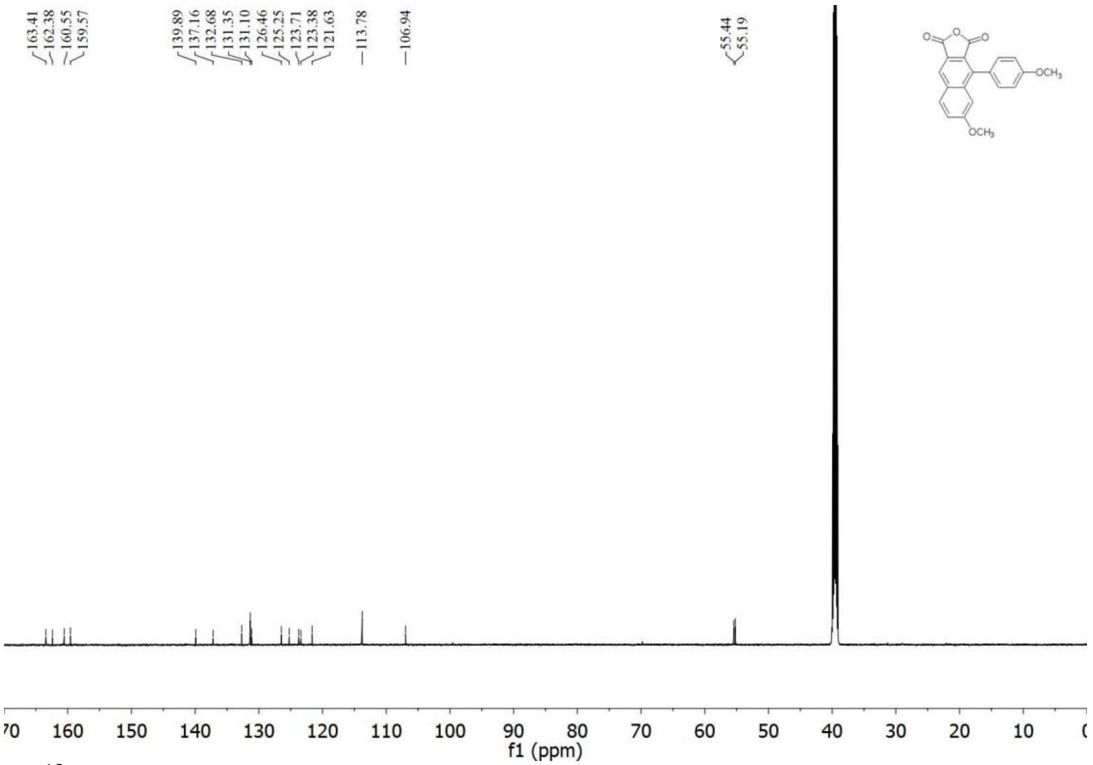

151 MHz ^{13}C NMR spectrum of compound **2b** recorded in $(CD_3)_2SO$ at T = 298 K.

4.3. 6-Methyl-4-(4-tolyl)naphtho[2,3-*c*]furan-1,3-dione (2c)

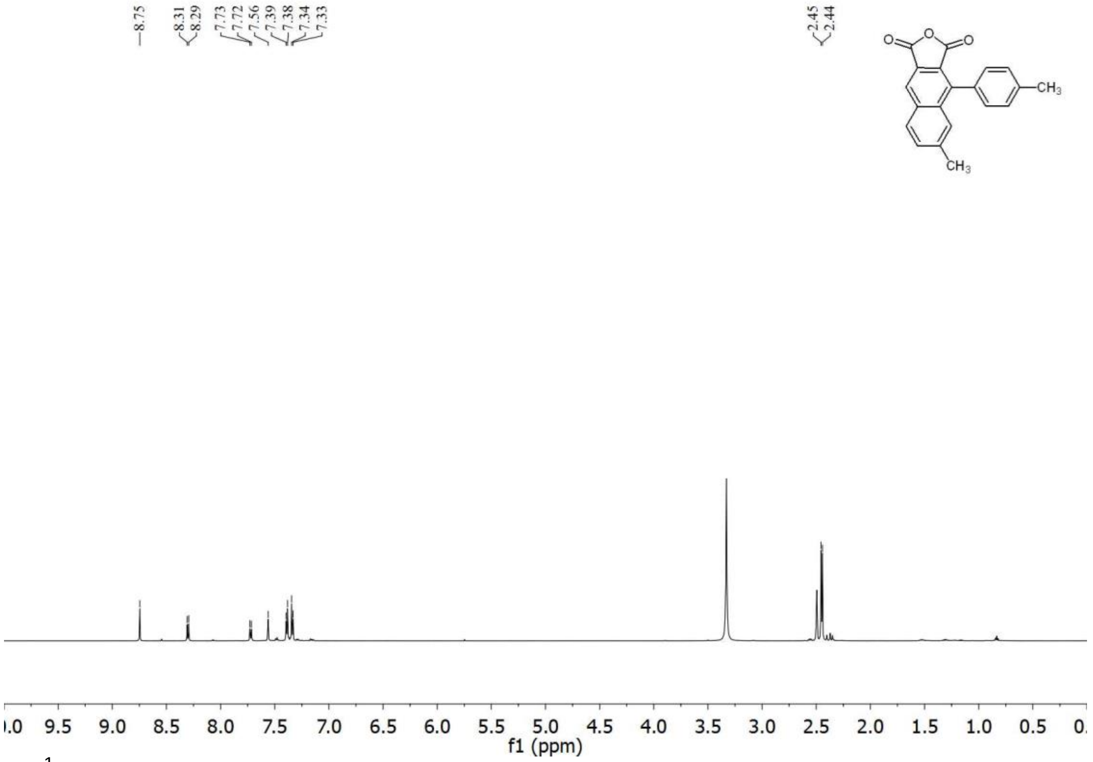

600 MHz ^{1}H NMR spectrum of compound **2c** recorded in $(CD_3)_2SO$ at T = 298 K.

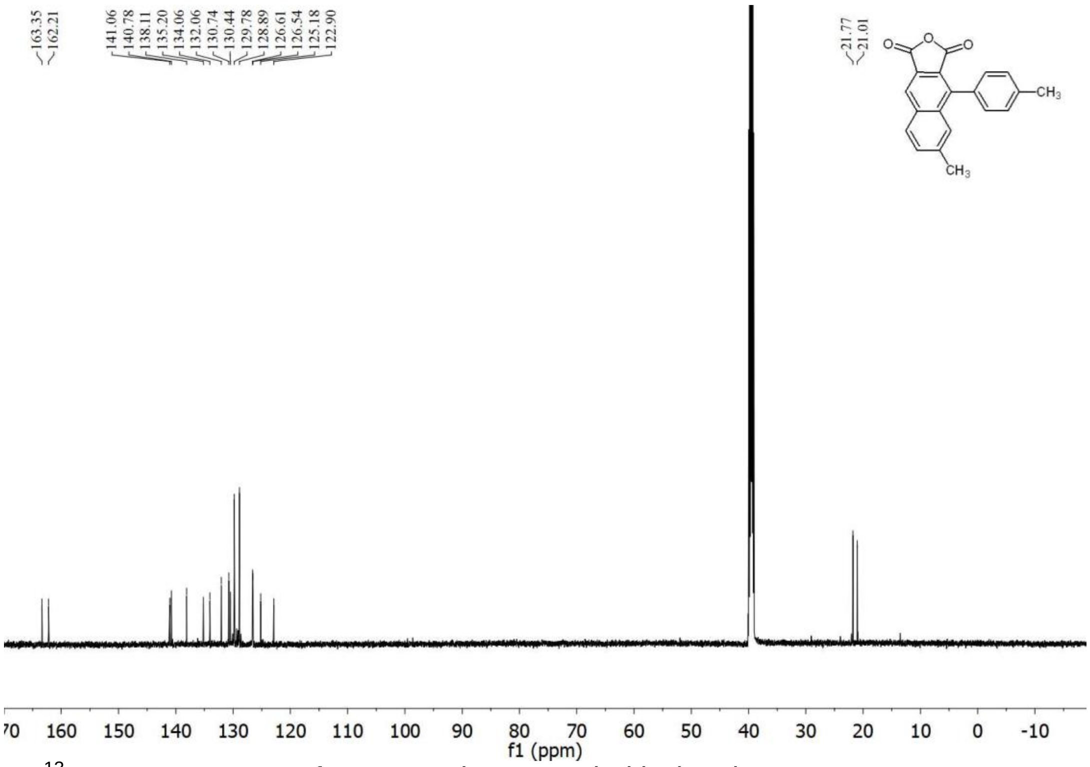

151 MHz ^{13}C NMR spectrum of compound **2c** recorded in $(CD_3)_2SO$ at T = 298 K.

4.4. 6-Chloro-4-(4-chlorophenyl)naphtho[2,3-*c*]furan-1,3-dione (2d)

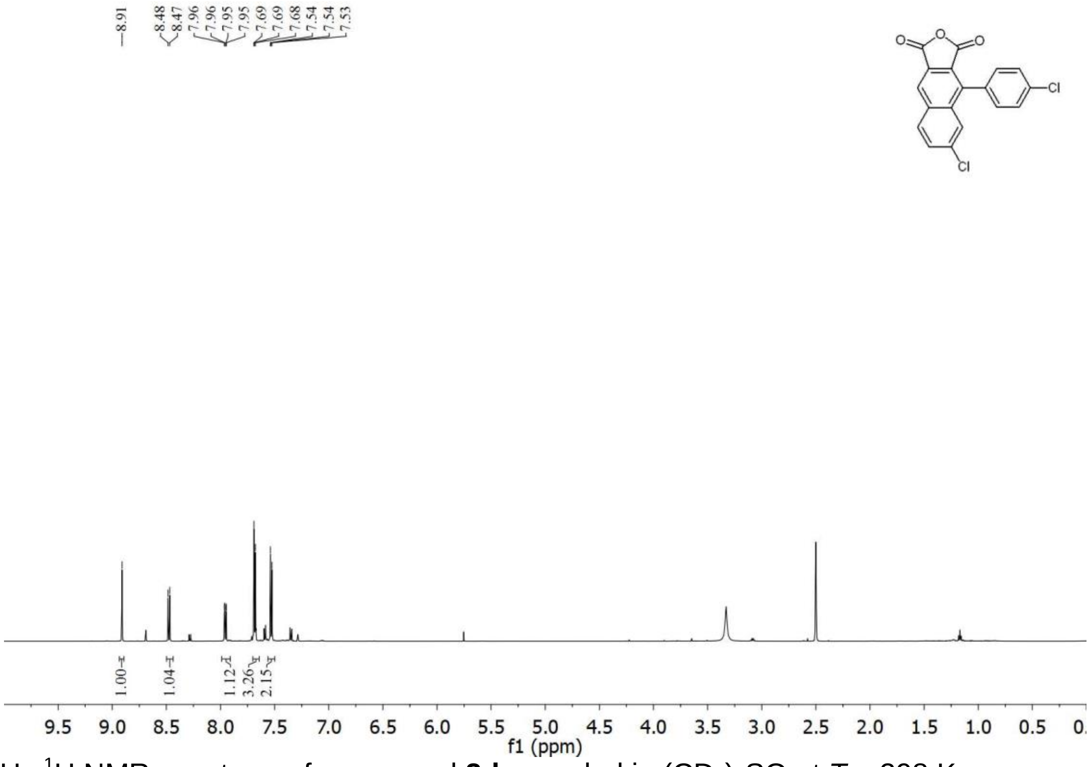

600 MHz ^{1}H NMR spectrum of compound **2d** recorded in $(CD_3)_2SO$ at T = 298 K.

4.5. NMR spectra of the diacid resulting from the hydrolysis of compound 2a

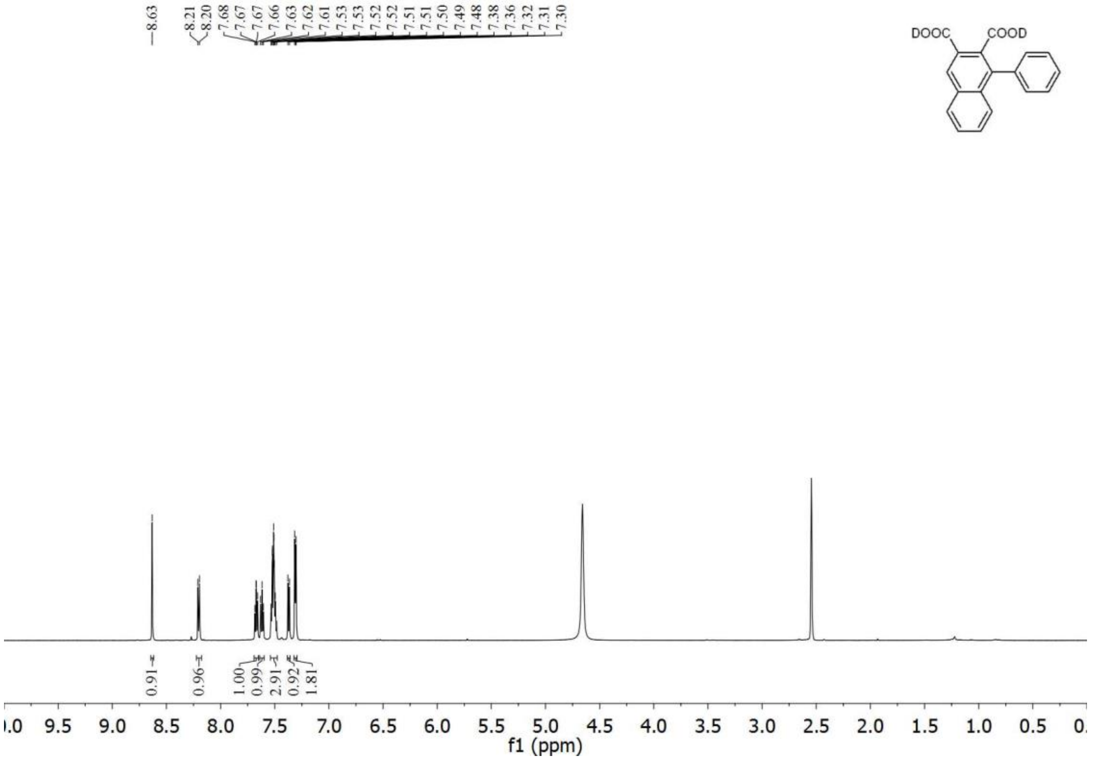

600 MHz ^{1}H NMR spectrum of the diacid recorded in $(CD_3)_2SO$ + 1.0 equiv of DCl at T = 298 K.

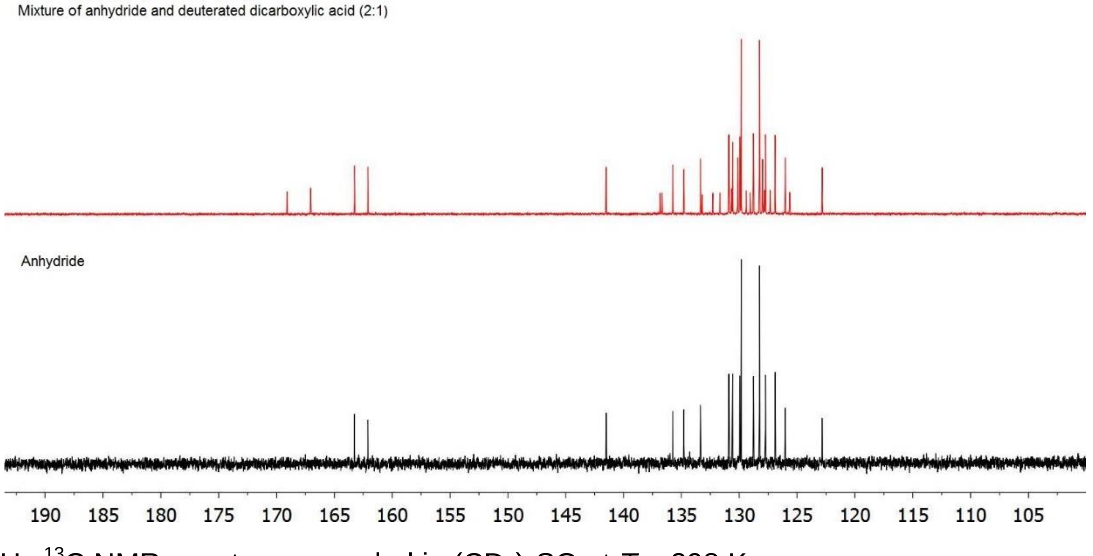

151 MHz ^{13}C NMR spectrum recorded in $(CD_3)_2SO$ at T = 298 K.

4.6. 2,4-Diphenyl-1*H*-benzo[*f*]isoindole-1,3(2*H*)-dione (4a)

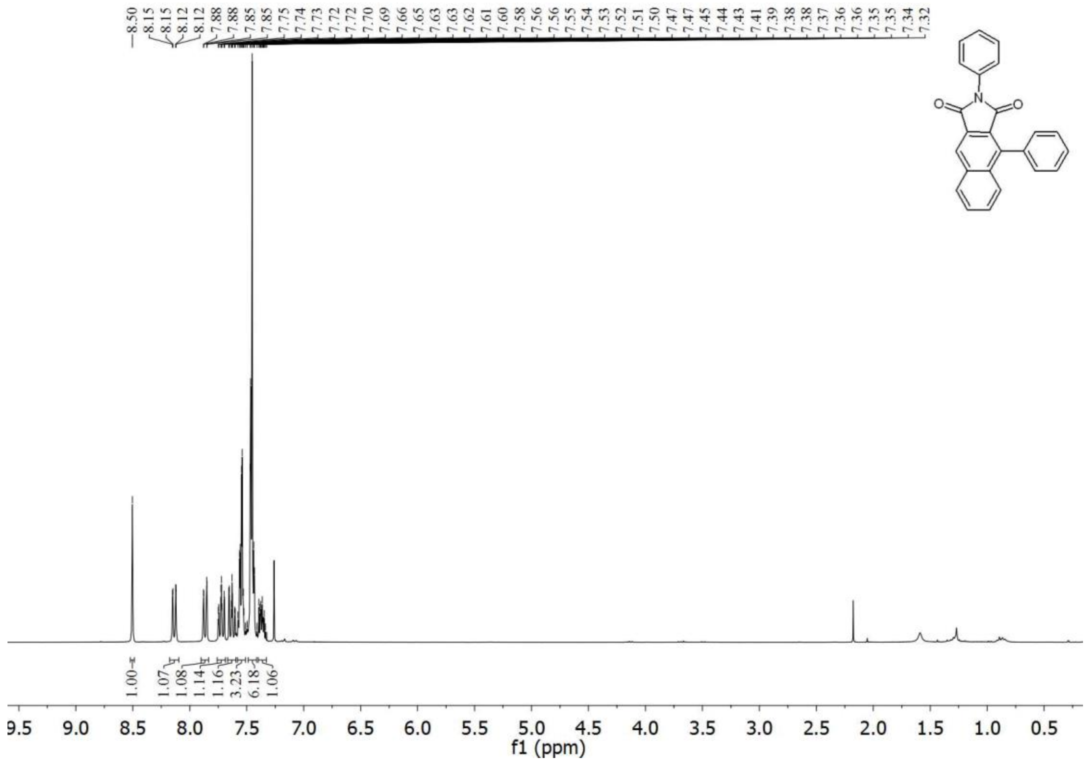

300 MHz ^{1}H NMR spectrum of compound **4a** recorded in $CDCl_3$ at T = 298 K.

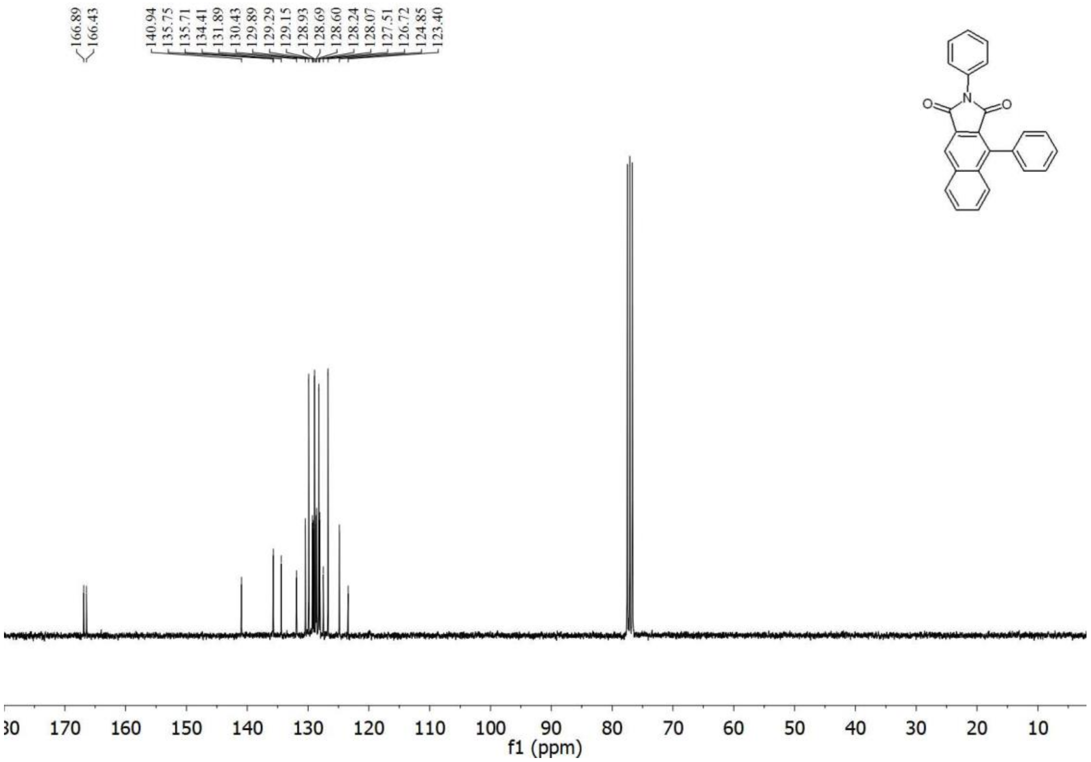

75 MHz ^{13}C NMR spectrum of compound **4a** recorded in $CDCl_3$ at T = 298 K.

4.7. 6-Methoxy-4-(4-methoxyphenyl)-2-phenyl-1*H*-benzo[*f*]isoindole-1,3(2*H*)-dione (4b)

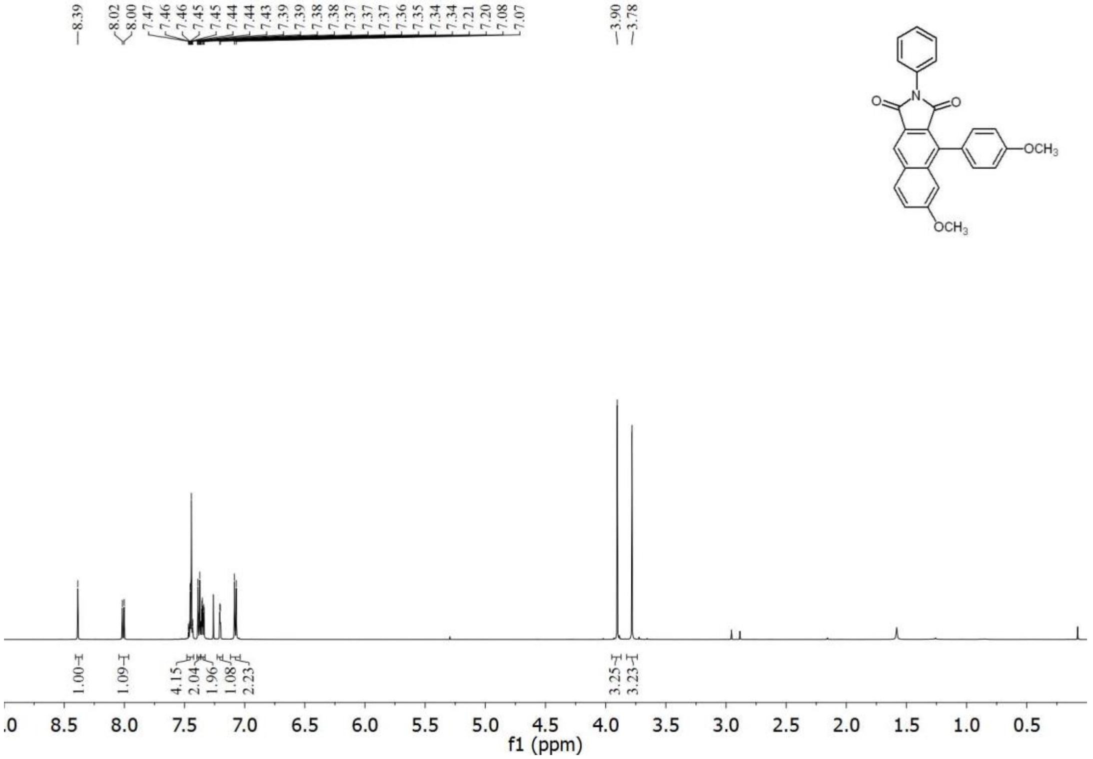

600 MHz ^{1}H NMR spectrum of compound **4b** recorded in $CDCl_3$ at T = 298 K.

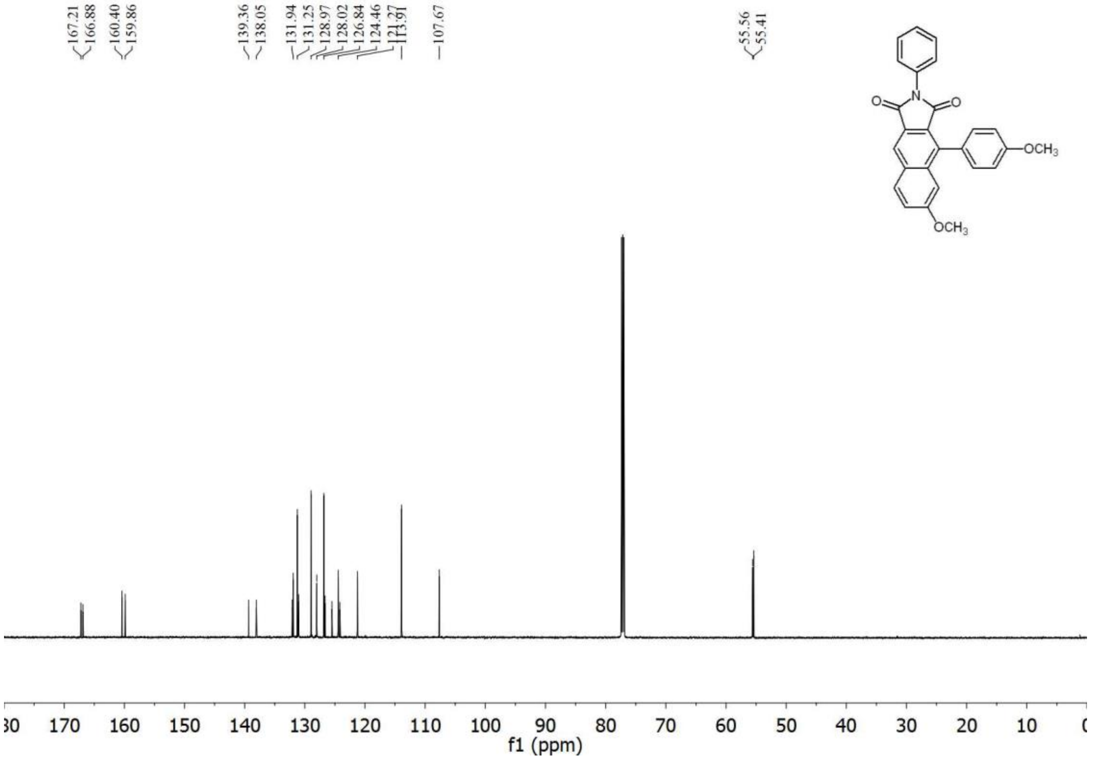

151 MHz ^{13}C NMR spectrum of compound **4b** recorded in $CDCl_3$ at T = 298 K.

4.8. 2-((10-Methyl-10*H*-phenothiazin-3-yl)methyl)-4-phenyl-1*H*-benzo[*f*]isoindole-1,3(2*H*)-dione (4c)

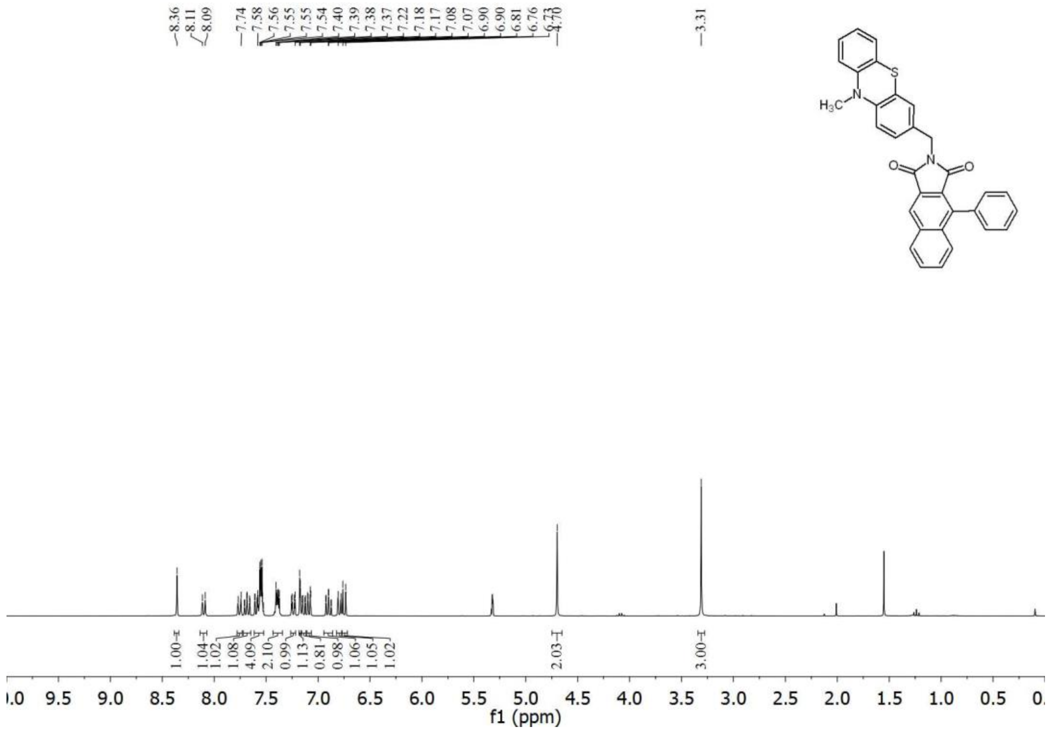

300 MHz ^{1}H NMR spectrum of compound **4c** recorded in CD_2Cl_2 at T = 298 K.

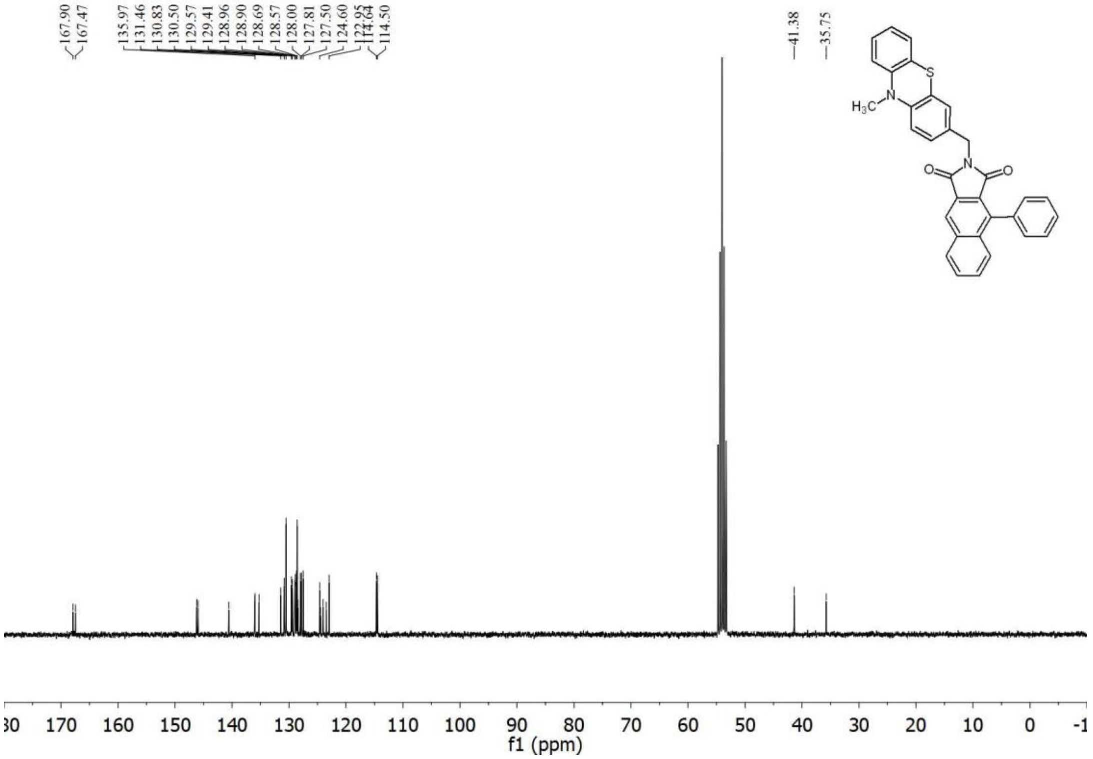

75 MHz ^{13}C NMR spectrum of compound **4c** recorded in CD_2Cl_2 at T = 298 K.

4.9. 4-(4-Cyanophenyl)-1,3-dioxo-2-phenyl-2,3-dihydro-1*H*-benzo[*f*]isoindole-6-carbonitrile (4d)

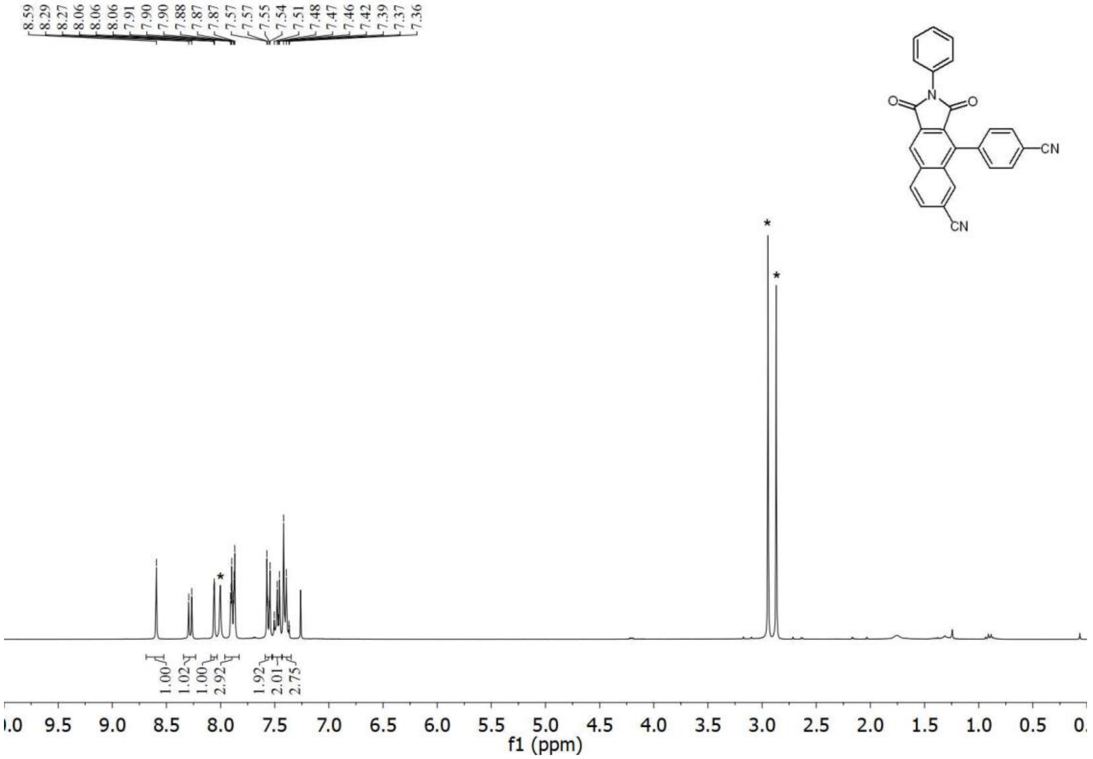

300 MHz ^{1}H NMR spectrum of compound **4d** recorded in $CDCl_3$ at T = 298 K; *residual DMF.

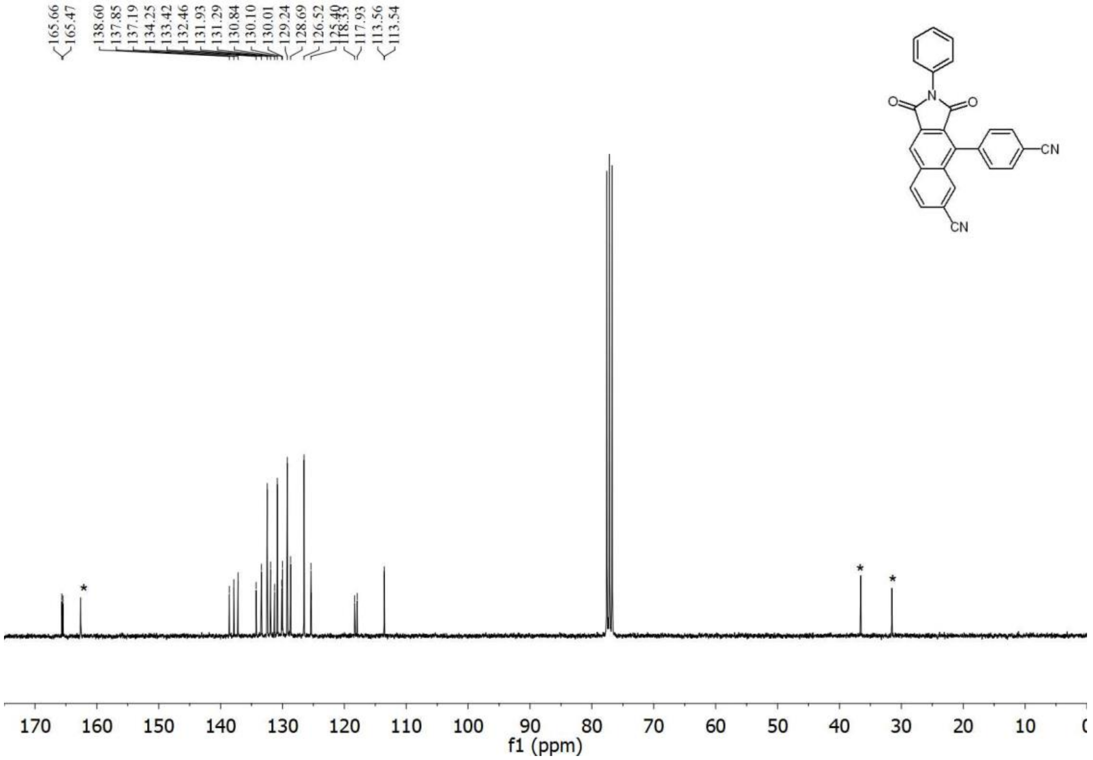

75 MHz ^{13}C NMR spectrum of compound **4d** recorded in $CDCl_3$ at T = 298 K; *residual DMF.

4.10. 6-Chloro-4-(4-chlorophenyl)-2-phenyl-1*H*-benzo[*f*]isoindole-1,3(2*H*)-dione (4e)

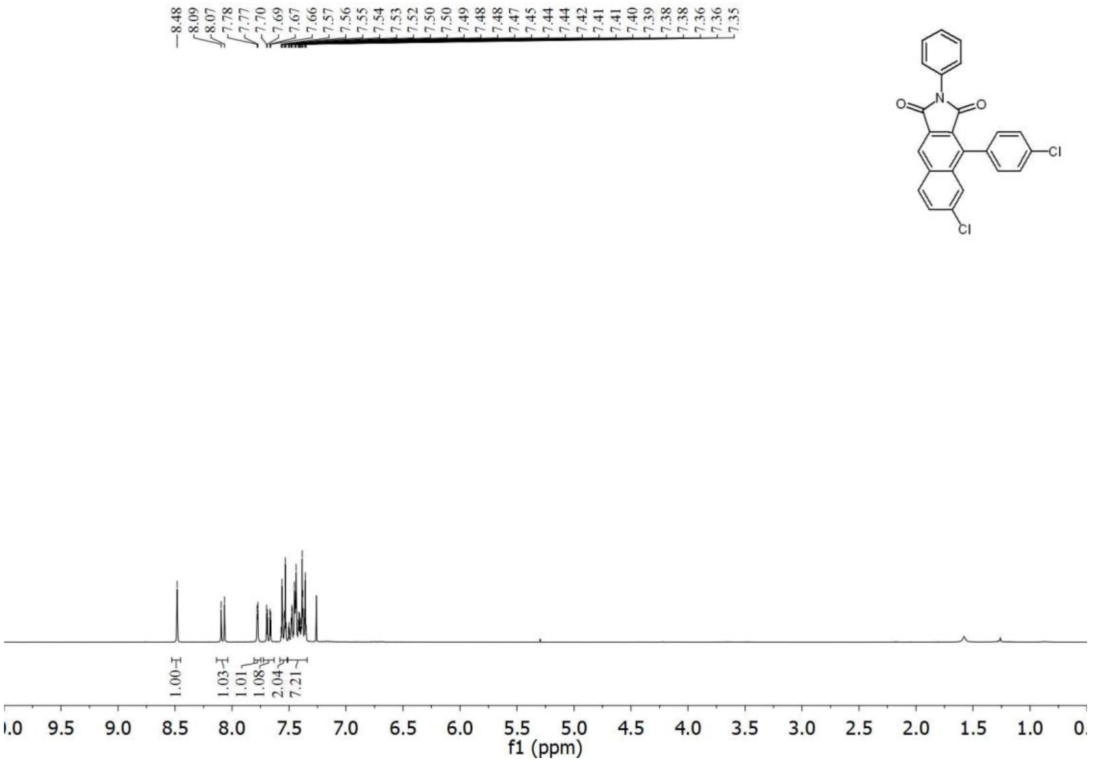

300 MHz ^{1}H NMR spectrum of compound **4e** recorded in $CDCl_3$ at T = 298 K.

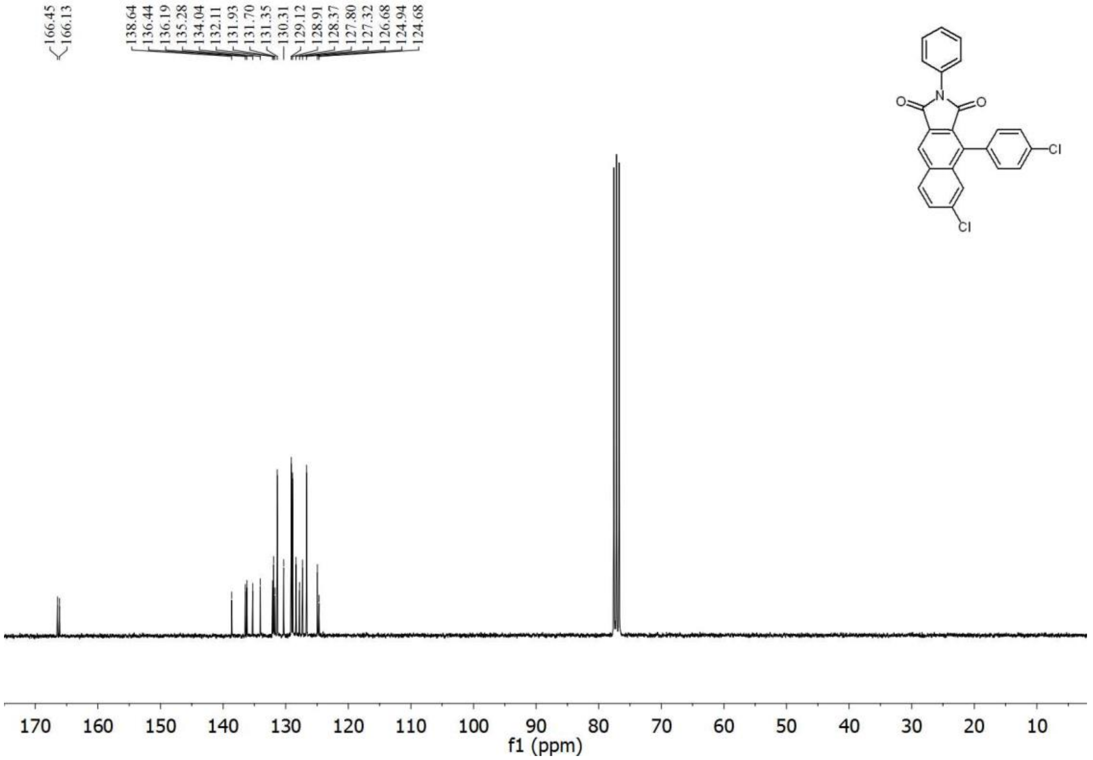

75 MHz ^{13}C NMR spectrum of compound **4e** recorded in $CDCl_3$ at T = 298 K.

4.11. 2-Phenyl-6-(trifluoromethyl)-4-(4-(trifluoromethyl)phenyl)-1*H*-benzo[*f*]isoindole-1,3(2*H*)-dione (4f)

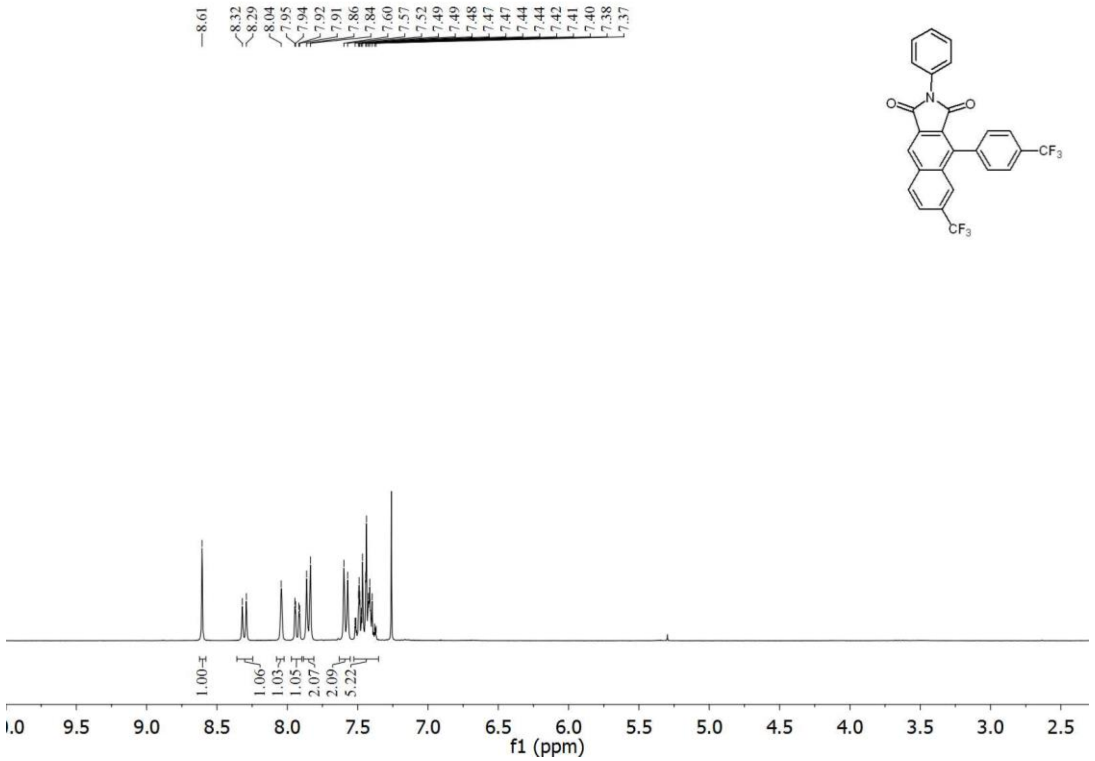

300 MHz ^{1}H NMR spectrum of compound **4f** recorded in $CDCl_3$ at T = 298 K.

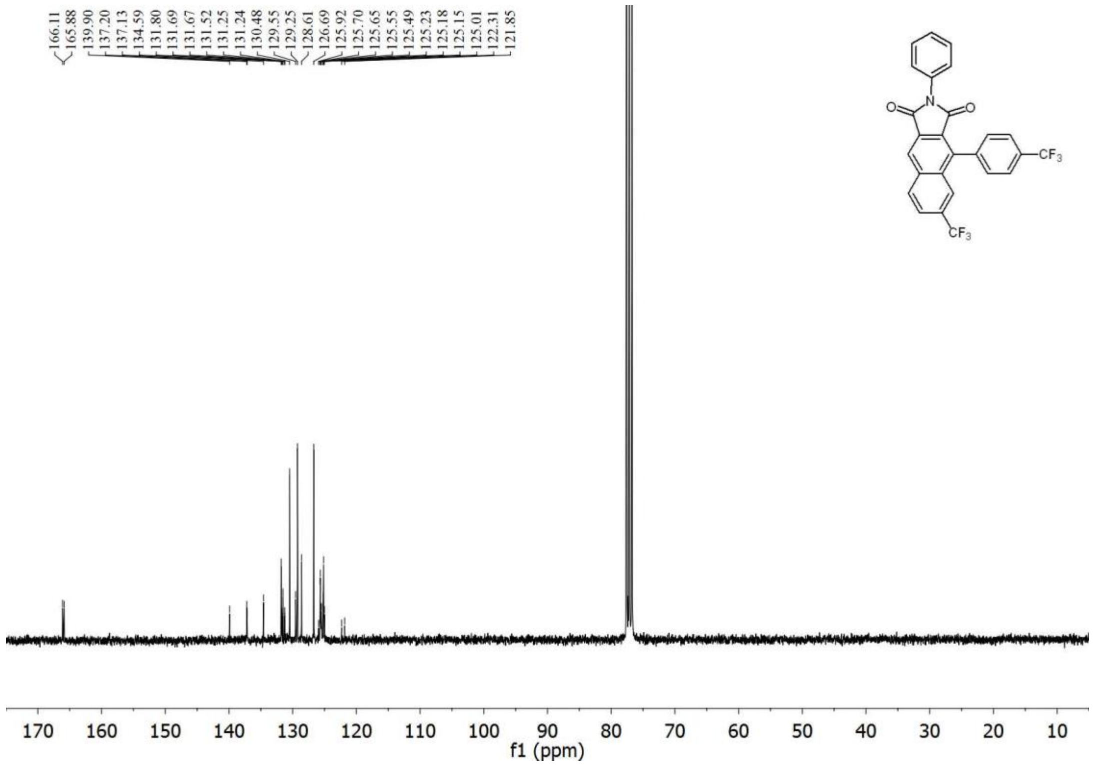

75 MHz ^{13}C NMR spectrum of compound **4f** recorded in $CDCl_3$ at T = 298 K.

4.12. 6-Methyl-2-phenyl-4-(4-tolyl)-1*H*-benzo[*f*]isoindole-1,3(2*H*)-dione (4g)

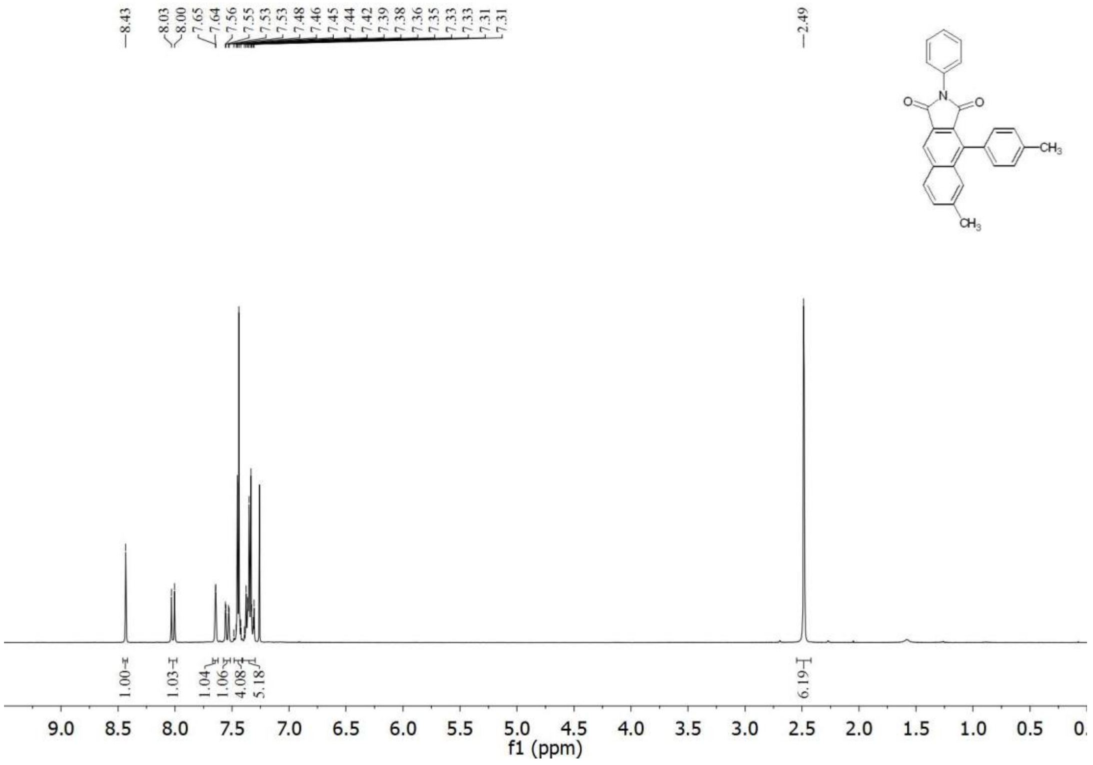

300 MHz ^{1}H NMR spectrum of compound **4g** recorded in $CDCl_3$ at T = 298 K.

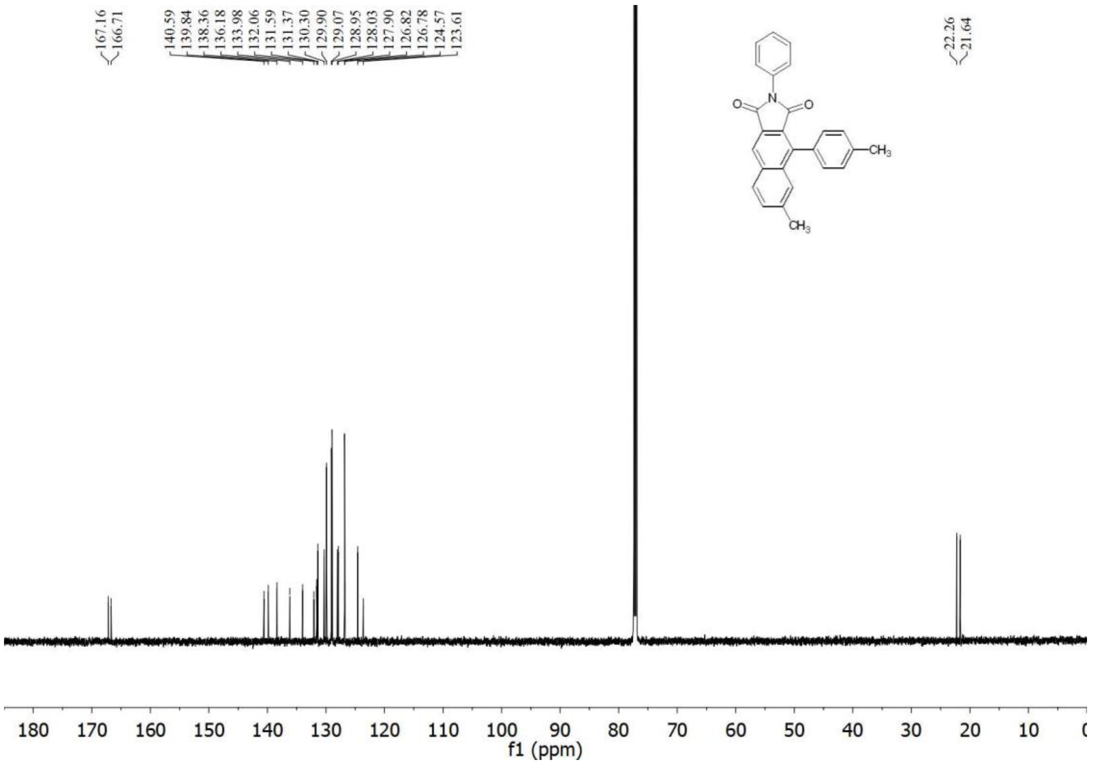

151 MHz ^{13}C NMR spectrum of compound **4g** recorded in $CDCl_3$ at T = 298 K.

4.13. 2-(4-Fluorophenyl)-4-phenyl-1*H*-benzo[*f*]isoindole-1,3(2*H*)-dione (4h)

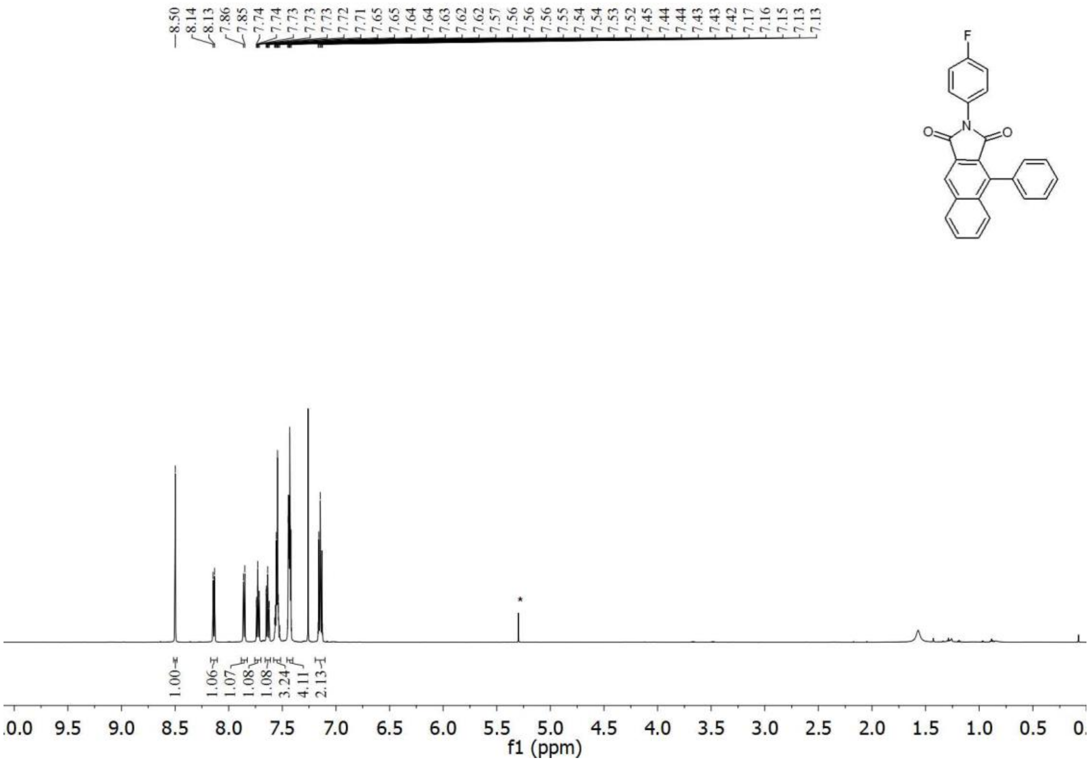

600 MHz ^{1}H NMR spectrum of compound **4h** recorded in $CDCl_3$ at T = 298 K;*residual dichloromethane.

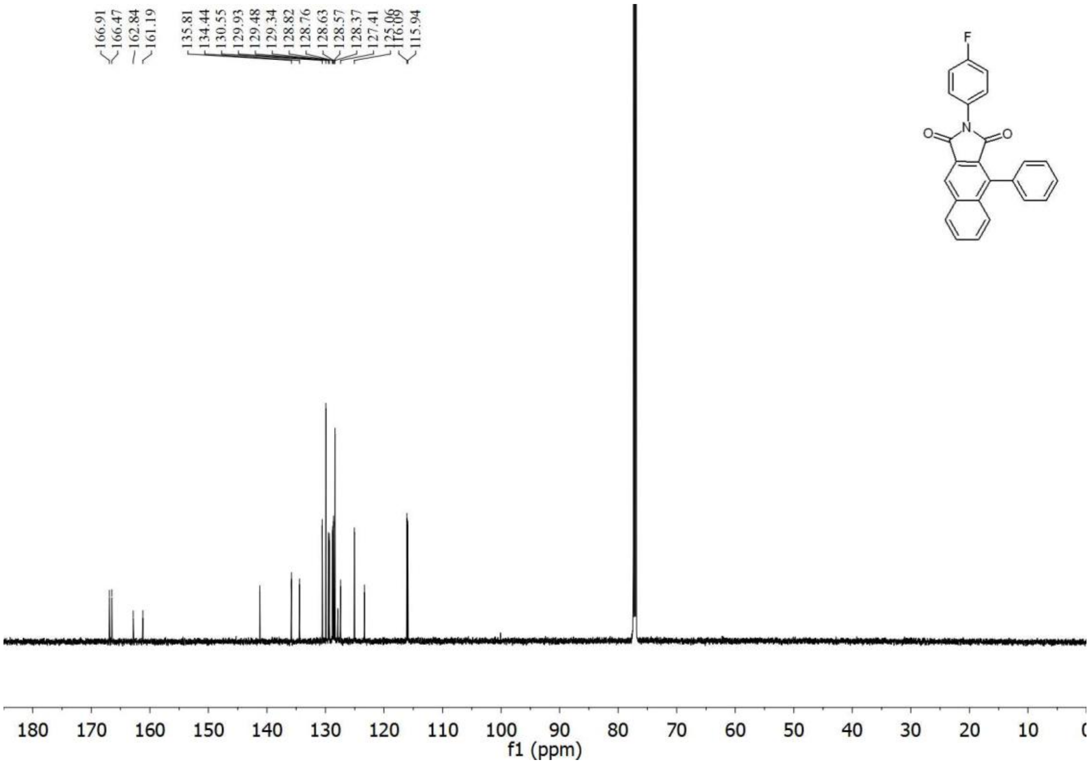

151 MHz ^{13}C NMR spectrum of compound **6h** recorded in $CDCl_3$ at T = 298 K.

4.14. 2-(4-Chlorophenyl)-4-phenyl-1*H*-benzo[*f*]isoindole-1,3(2*H*)-dione (4i)

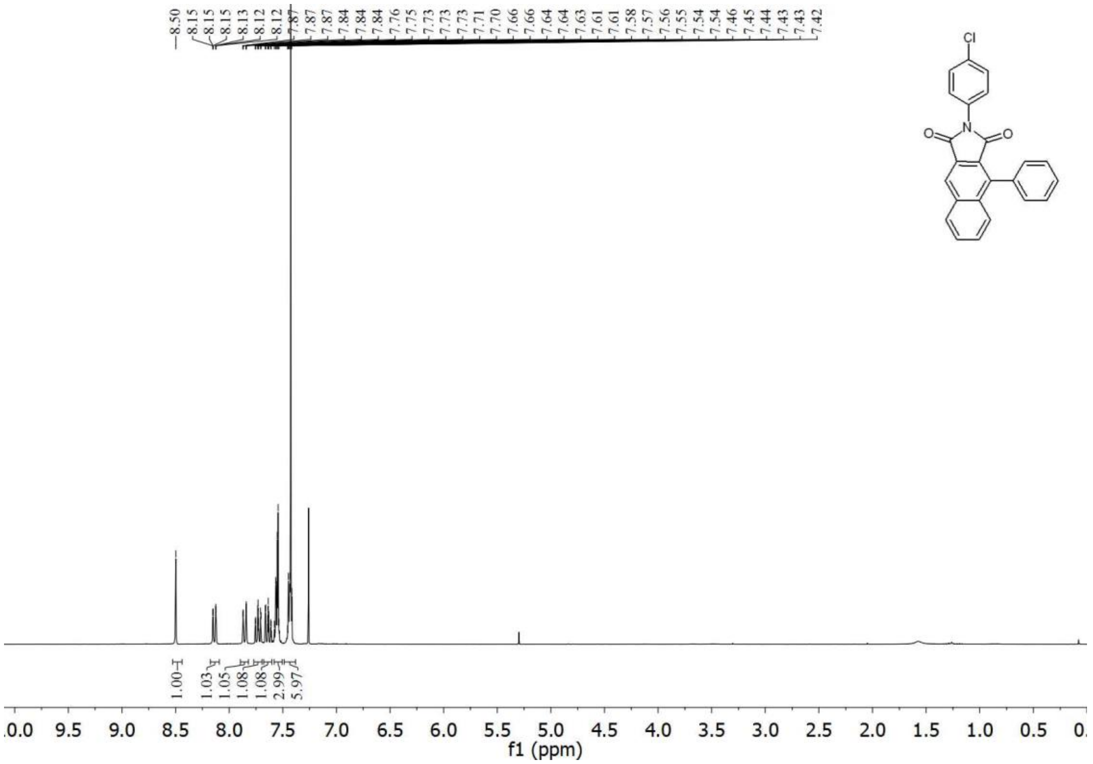

300 MHz ^{1}H NMR spectrum of compound **4i** recorded in $CDCl_3$ at T = 298 K.

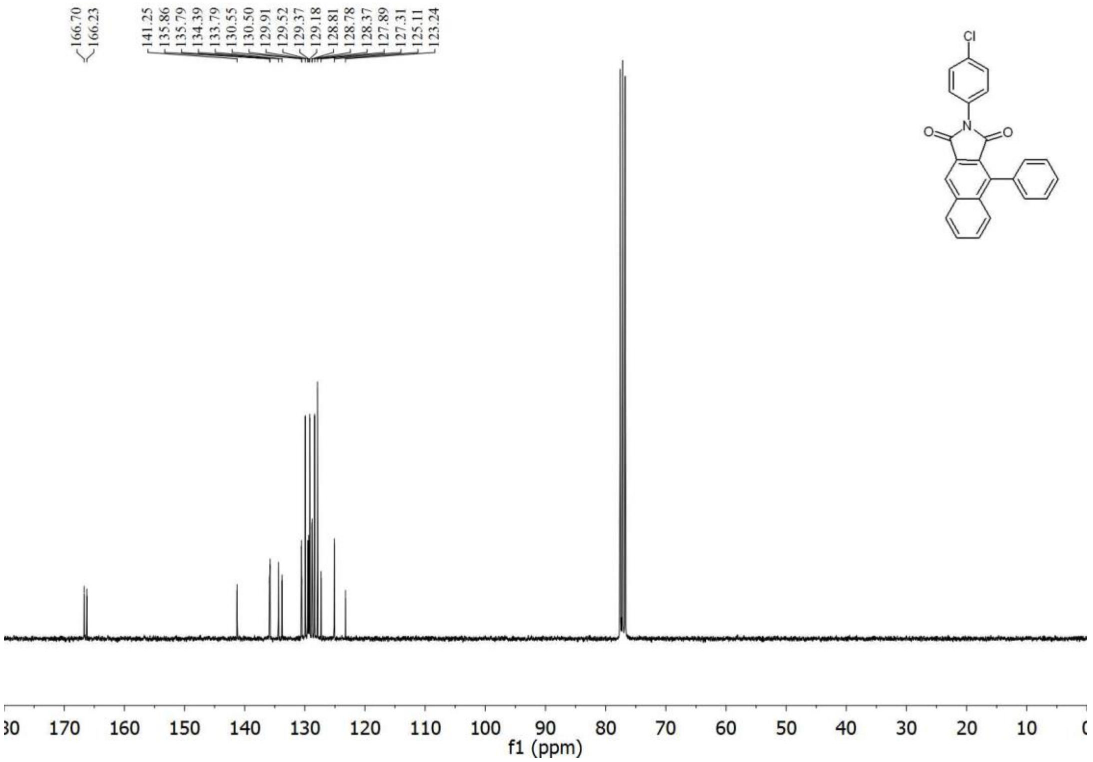

75 MHz ^{13}C NMR spectrum of compound **4i** recorded in $CDCl_3$ at T = 298 K.

4.15. 2-(4-Iodophenyl)-4-phenyl-1*H*-benzo[*f*]isoindole-1,3(2*H*)-dione (4j)

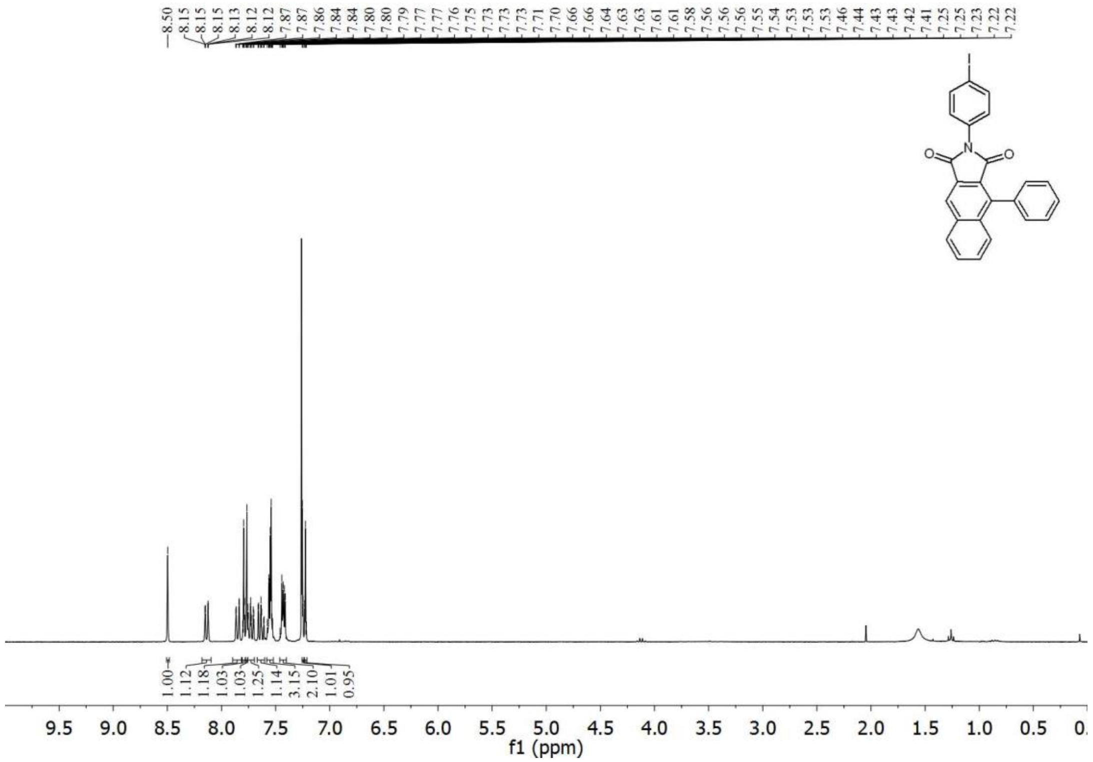

300 MHz ^{1}H NMR spectrum of compound **4j** recorded in $CDCl_3$ at T = 298 K.

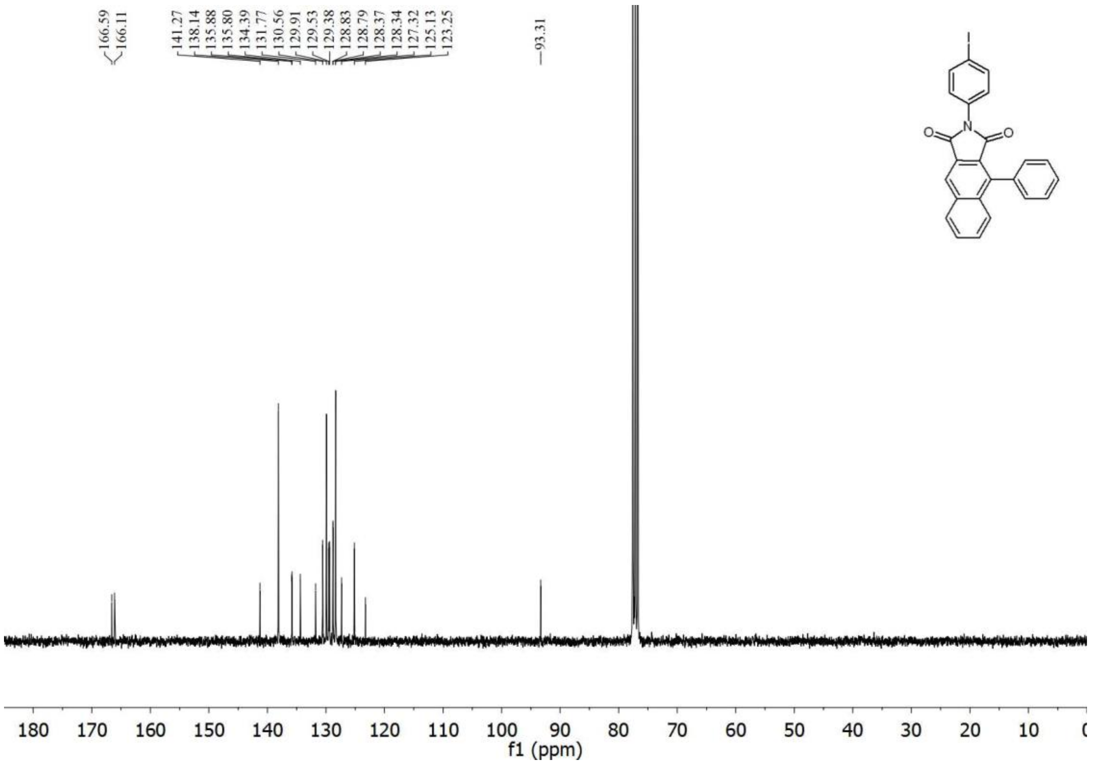

75 MHz ^{13}C NMR spectrum of compound **4j** recorded in $CDCl_3$ at T = 298 K.

4.16. Ethyl-4-(1,3-dioxo-4-phenyl-1,3-dihydro-2*H*-benzo[*f*]isoindol-2-yl)benzoate (4k)

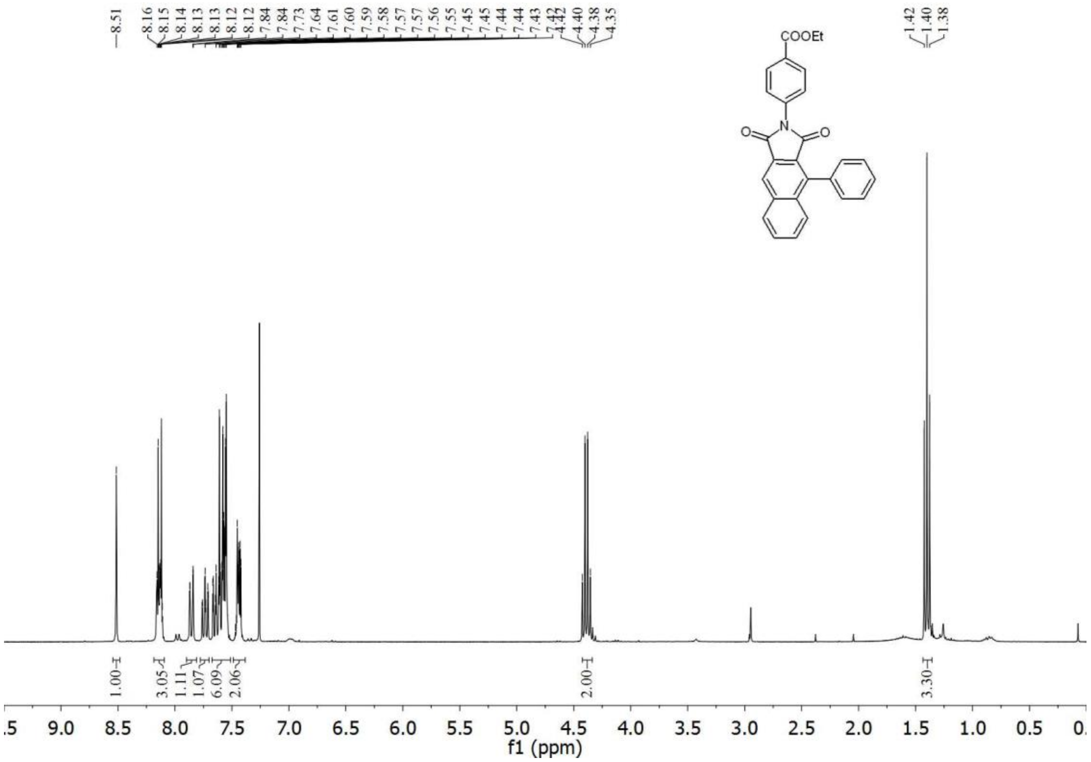

300 MHz ^{1}H NMR spectrum of compound **4k** recorded in $CDCl_3$ at T = 298 K.

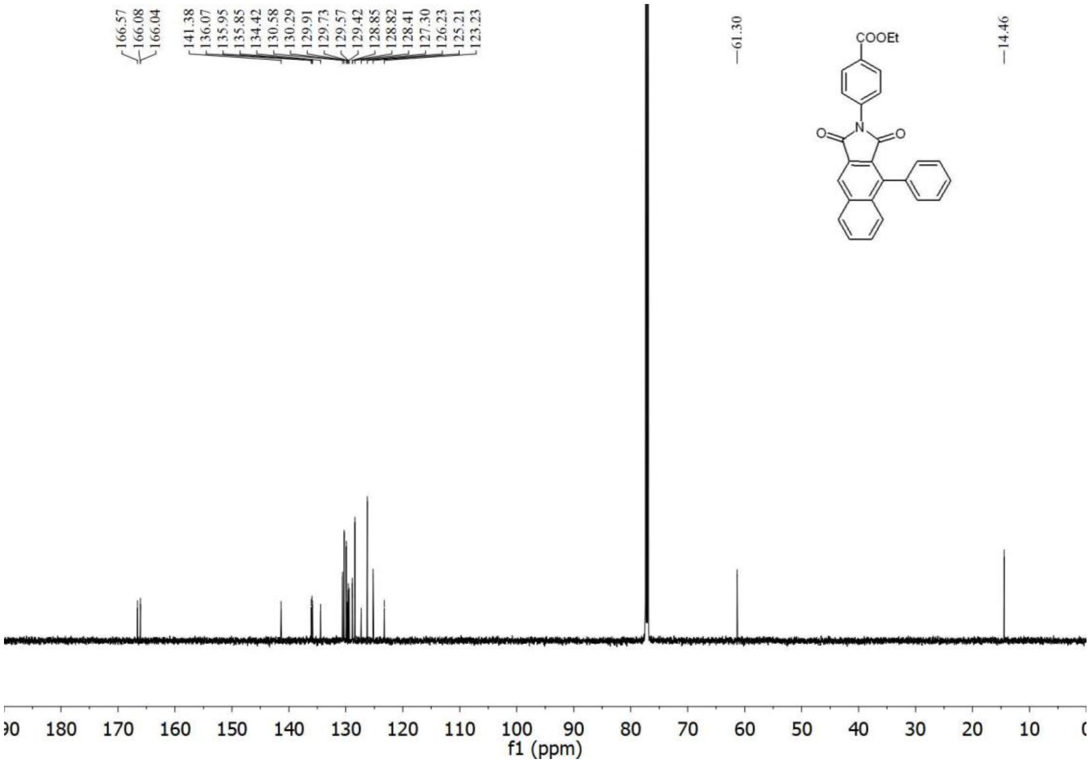

75 MHz ^{13}C NMR spectrum of compound **4k** recorded in $CDCl_3$ at T = 298 K.

4.17. 2-(3,5-Dimethylphenyl)-4-phenyl-1*H*-benzo[*f*]isoindole-1,3(2*H*)-dione (4l)

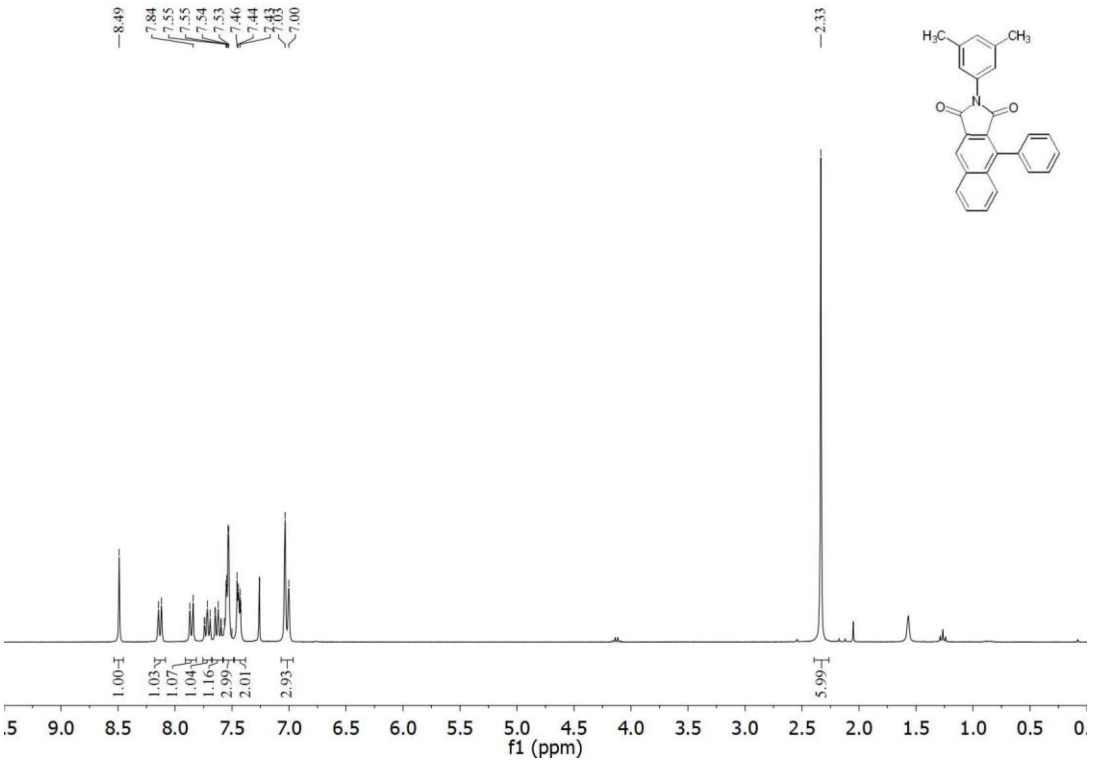

300 MHz ^{1}H NMR spectrum of compound **4l** recorded in $CDCl_3$ at T = 298 K.

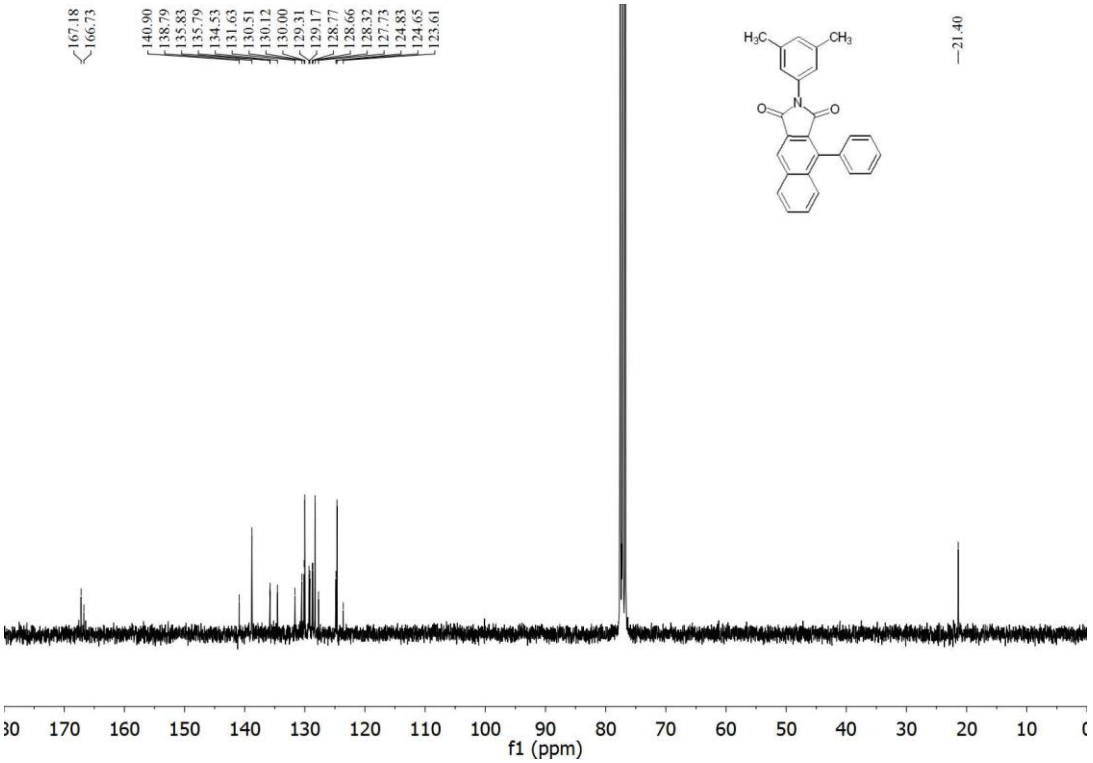

75 MHz ^{13}C NMR spectrum of compound **4l** recorded in $CDCl_3$ at T = 298 K.

4.18. 2-(2,6-Dimethylphenyl)-4-phenyl-1*H*-benzo[*f*]isoindole-1,3(2*H*)-dione (4m)

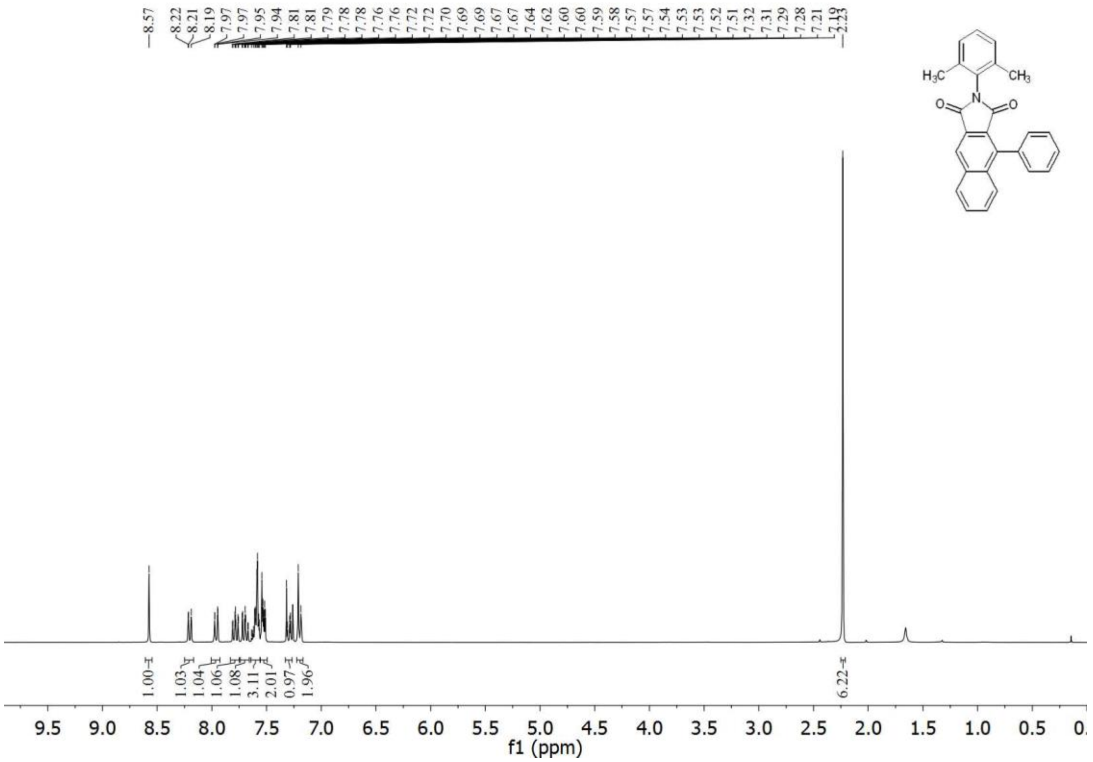

300 MHz ^{1}H NMR spectrum of compound **4m** recorded in $CDCl_3$ at T = 298 K.

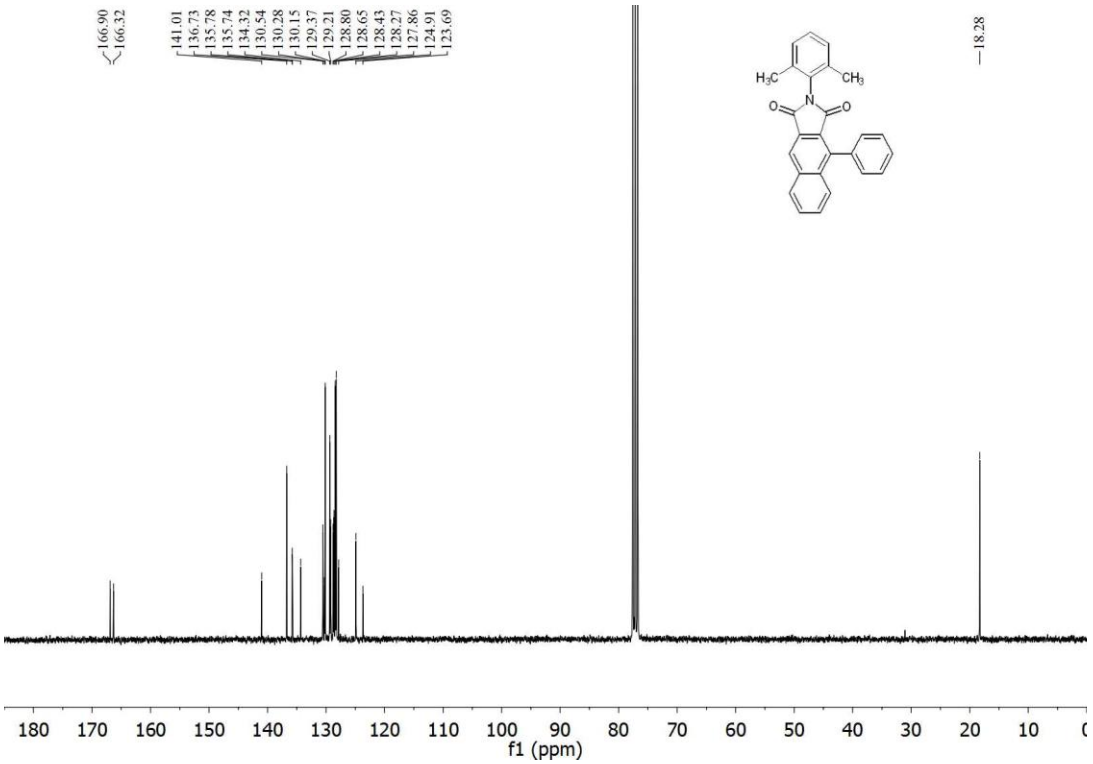

75 MHz ^{13}C NMR spectrum of compound **4m** recorded in $CDCl_3$ at T = 298 K.

4.19. 2-(3,5-Dimethoxyphenyl)-4-phenyl-1*H*-benzo[*f*]isoindole-1,3(2*H*)-dione (4n)

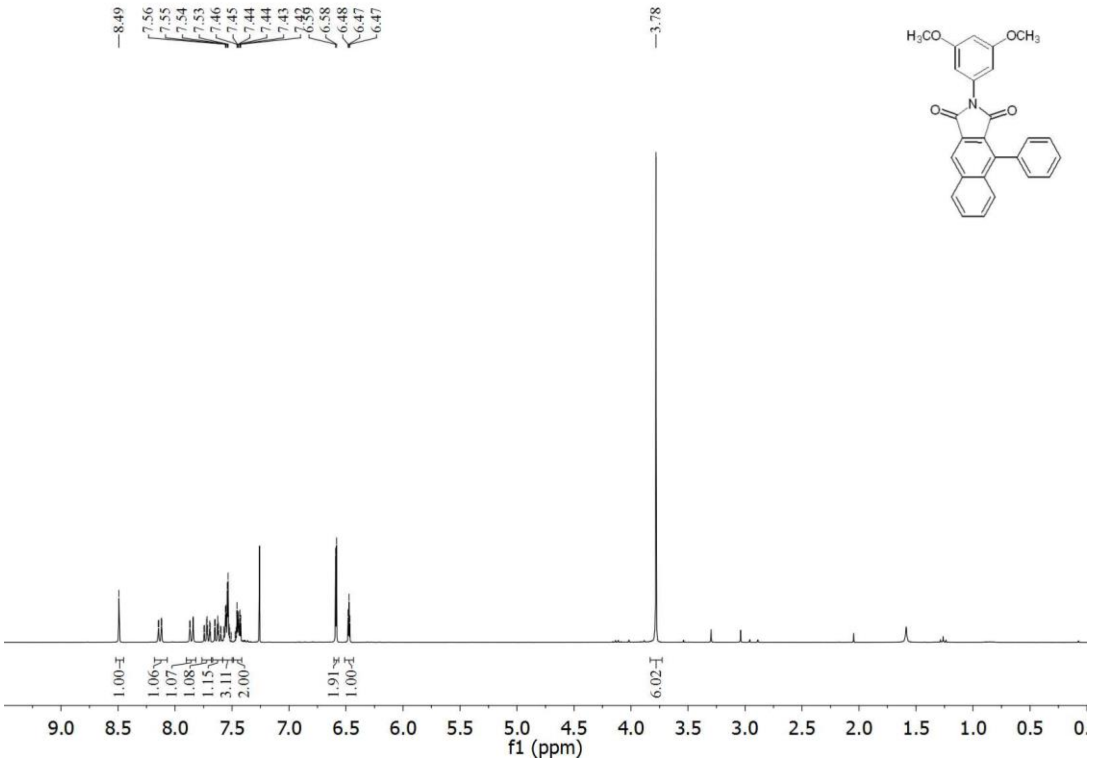

300 MHz ^{1}H NMR spectrum of compound **4n** recorded in $CDCl_3$ at T = 298 K.

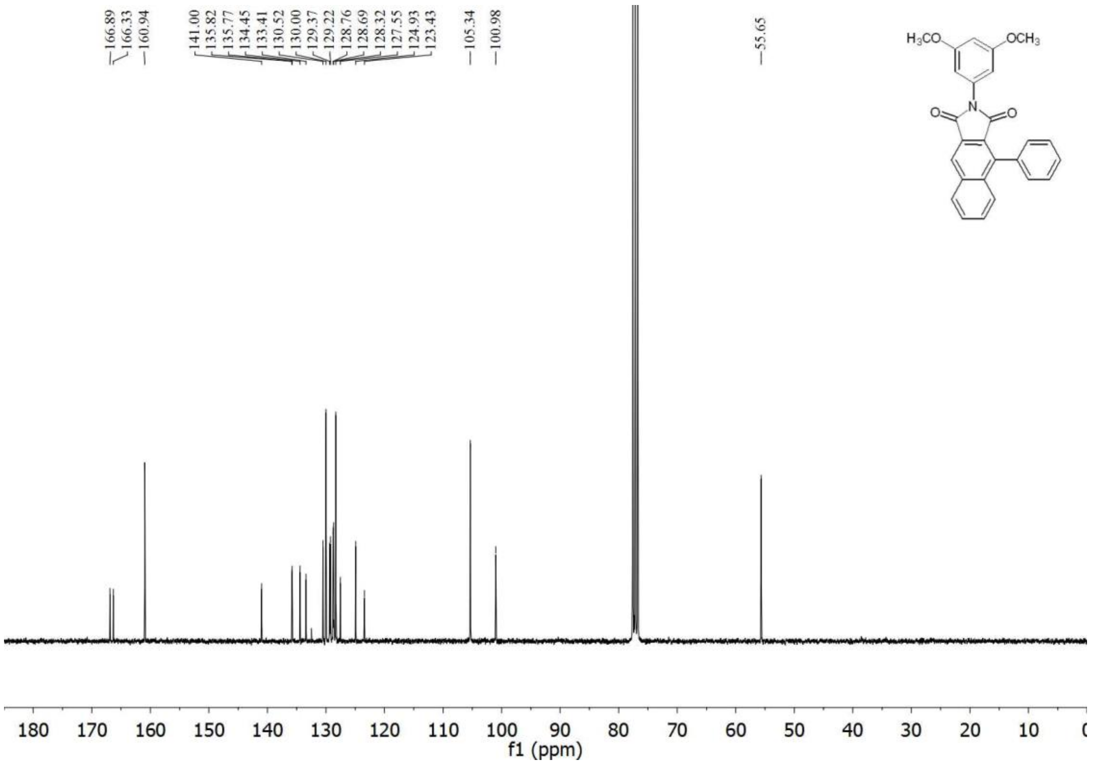

75 MHz ^{13}C NMR spectrum of compound **4n** recorded in $CDCl_3$ at T = 298 K.

4.20. 2-Benzyl-4-phenyl-1*H*-benzo[*f*]isoindole-1,3(2*H*)-dione (4o)

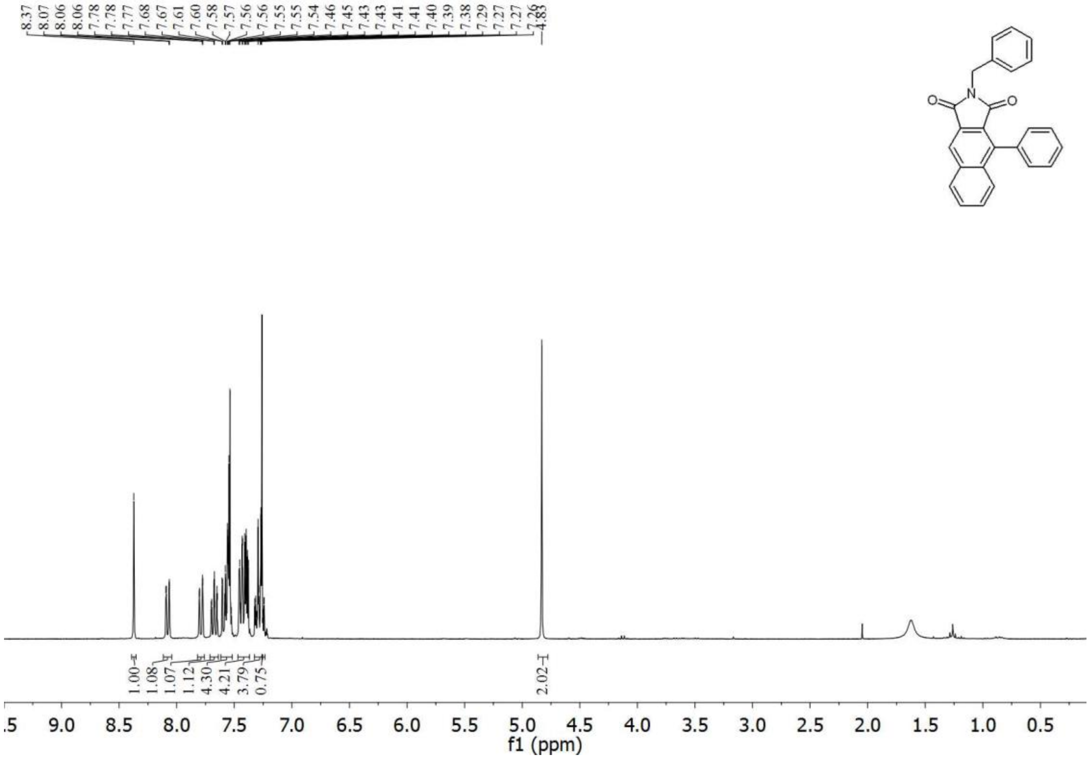

300 MHz ^{1}H NMR spectrum of compound **4o** recorded in $CDCl_3$ at T = 298 K.

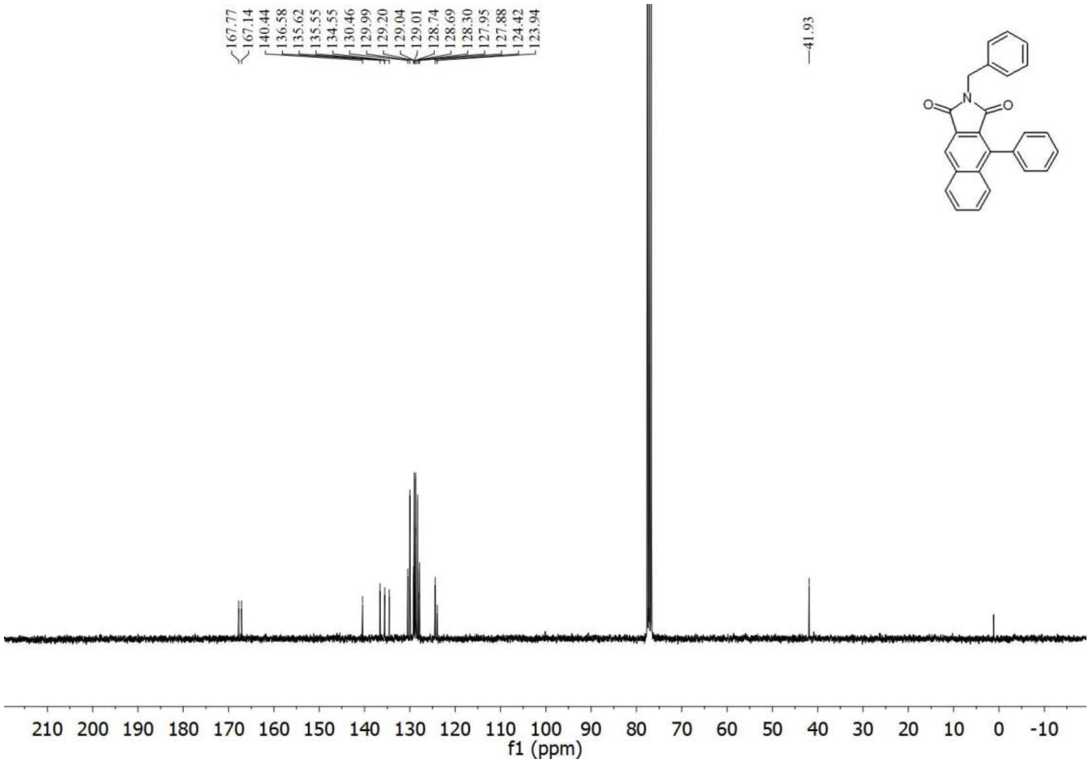

75 MHz ^{13}C NMR spectrum of compound **4o** recorded in $CDCl_3$ at T = 298 K.

4.21. 4-Phenyl-2-(prop-2-yn-1-yl)-1*H*-benzo[*f*]isoindole-1,3(2*H*)-dione (4p)

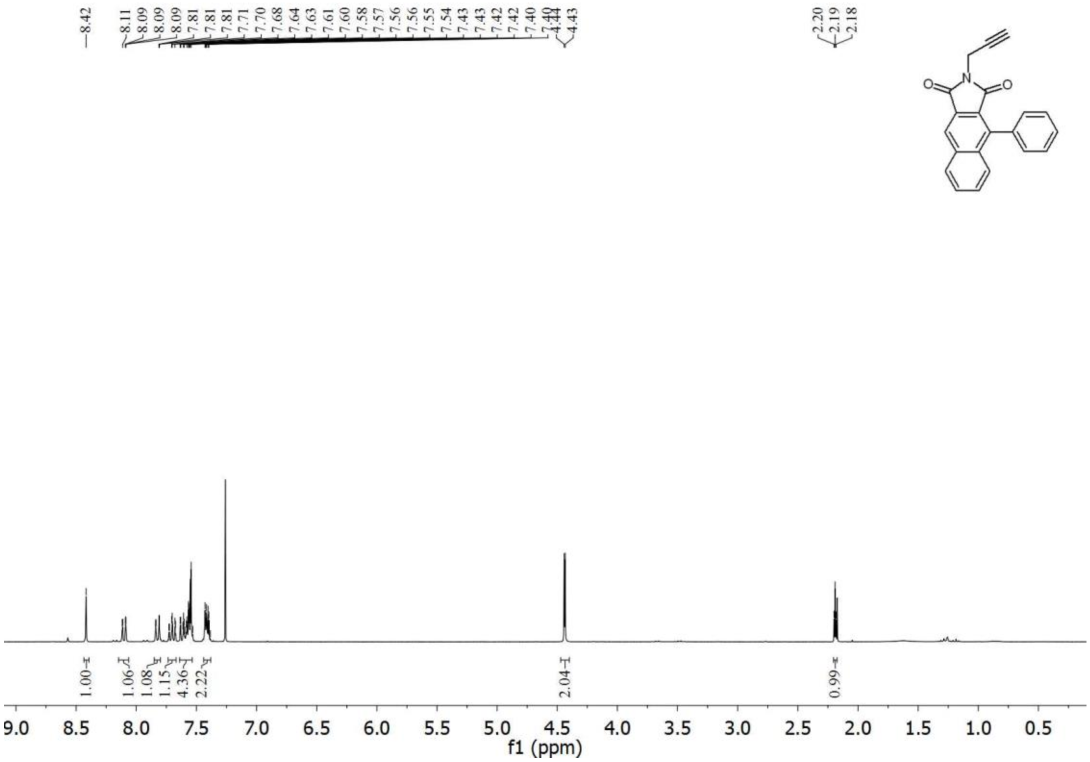

300 MHz ^{1}H NMR spectrum of compound **4p** recorded in $CDCl_3$ at T = 298 K.

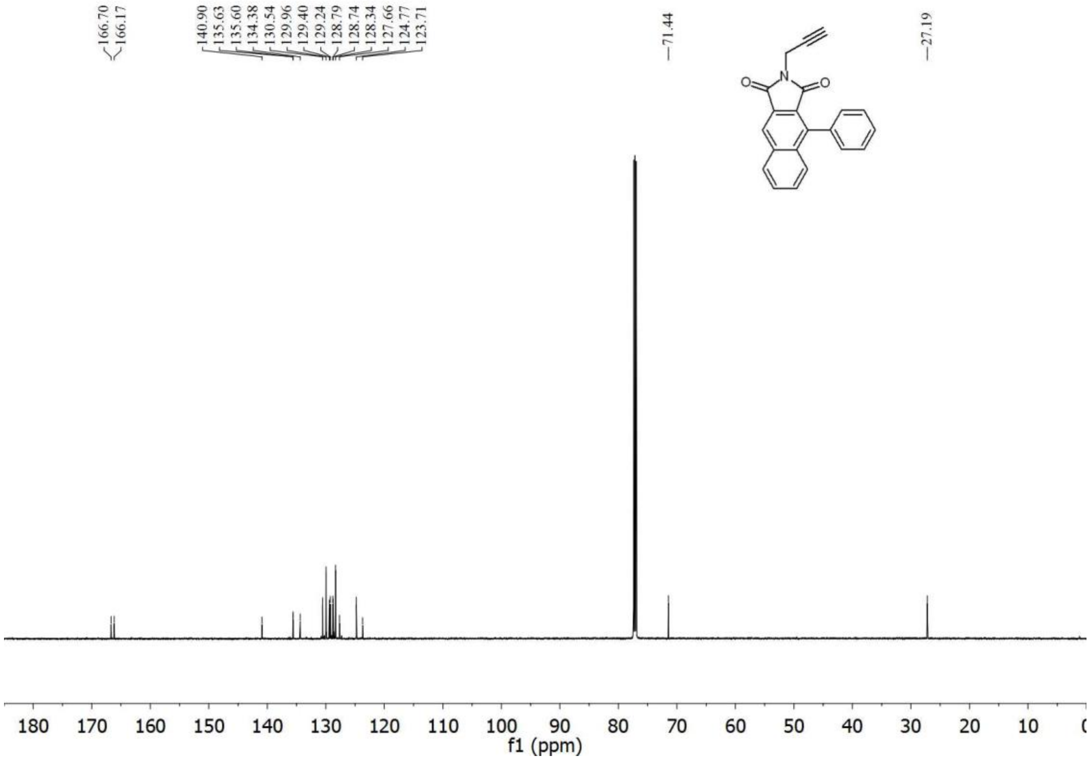

151 MHz ^{13}C NMR spectrum of compound **4p** recorded in $CDCl_3$ at T = 298 K.

4.22. 2-*n*-Hexyl-4-phenyl-1*H*-benzo[*f*]isoindole-1,3(2*H*)-dione (4q)

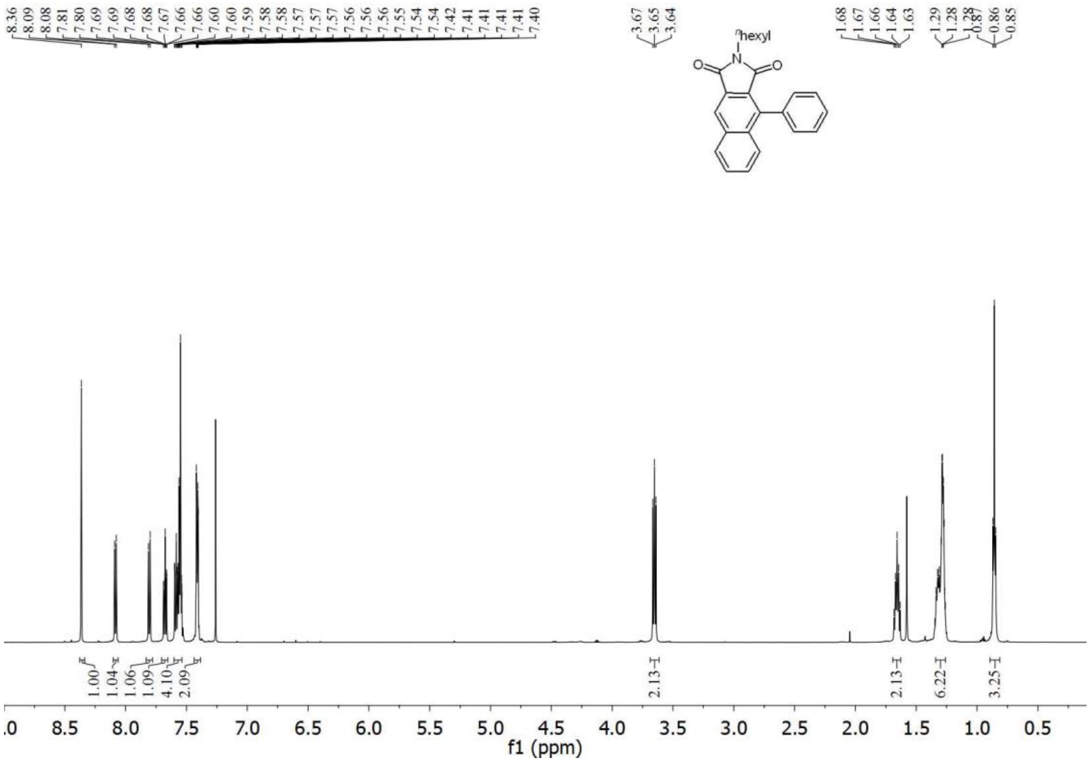

600 MHz ^{1}H NMR spectrum of compound **4q** recorded in $CDCl_3$ at *T* = 298 K.

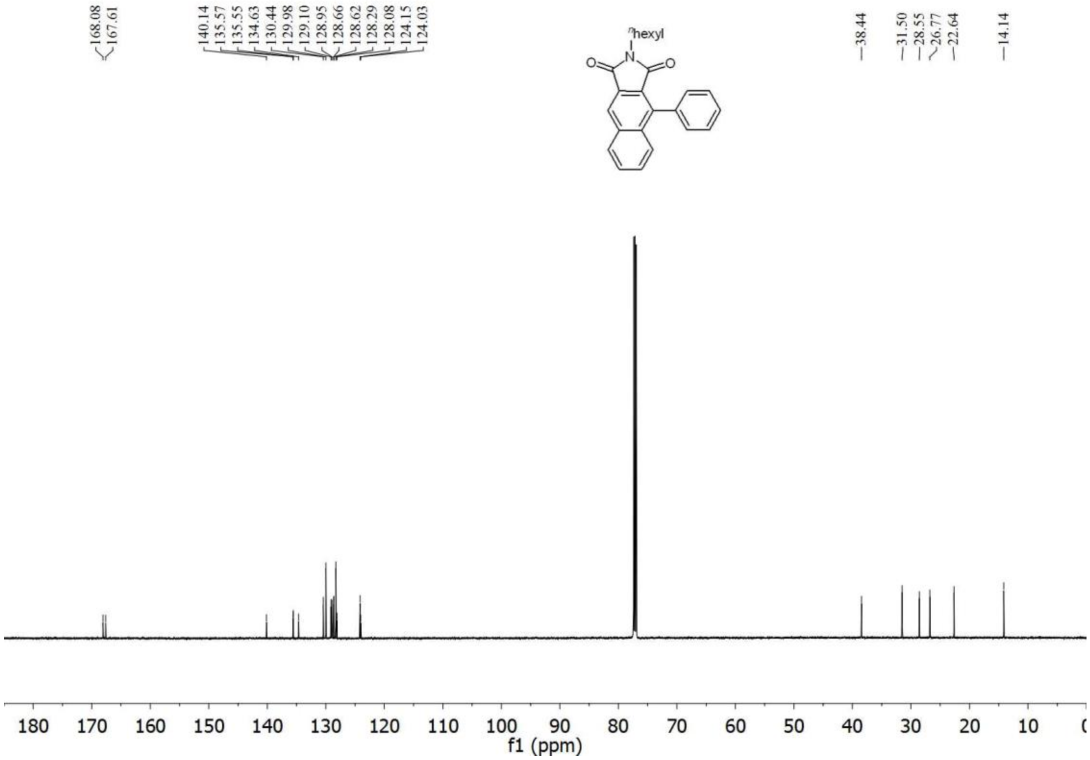

151 MHz ^{13}C NMR spectrum of compound **4q** recorded in $CDCl_3$ at *T* = 298 K.

4.23. 2-*n*-Butyl-4-phenyl-1*H*-benzo[*f*]isoindole-1,3(2*H*)-dione (4r)

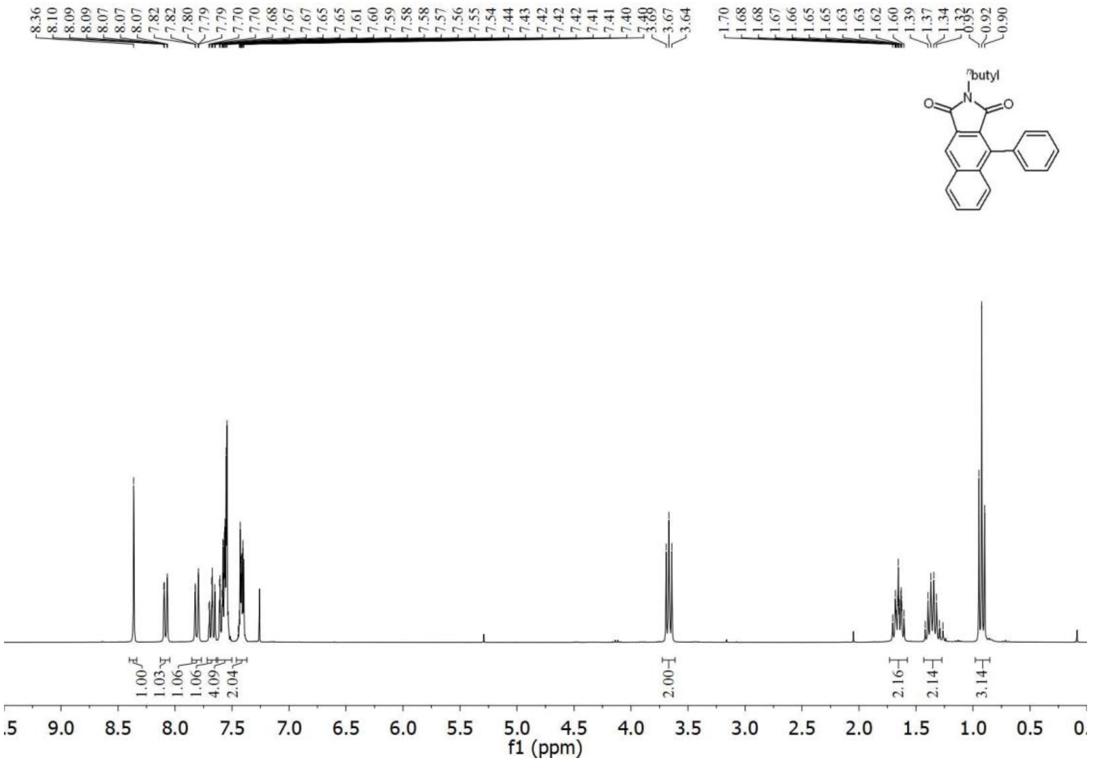

300 MHz ^{1}H NMR spectrum of compound **4r** recorded in $CDCl_3$ at T = 298 K.

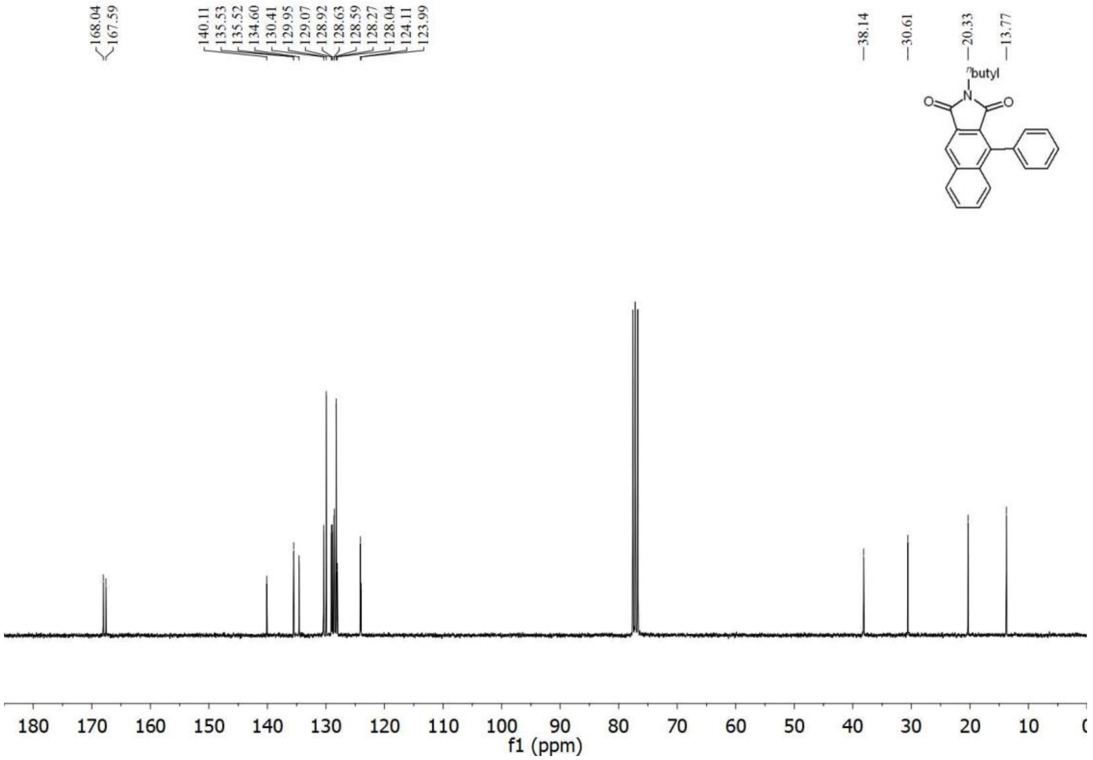

75 MHz ^{13}C NMR spectrum of compound **4r** recorded in $CDCl_3$ at T = 298 K.

4.24. 2-(2,6-Dimethylphenyl)-6-methoxy-4-(4-methoxyphenyl)-1*H*-benzo[*f*]isoindole-1,3(2*H*)-dione (4s)

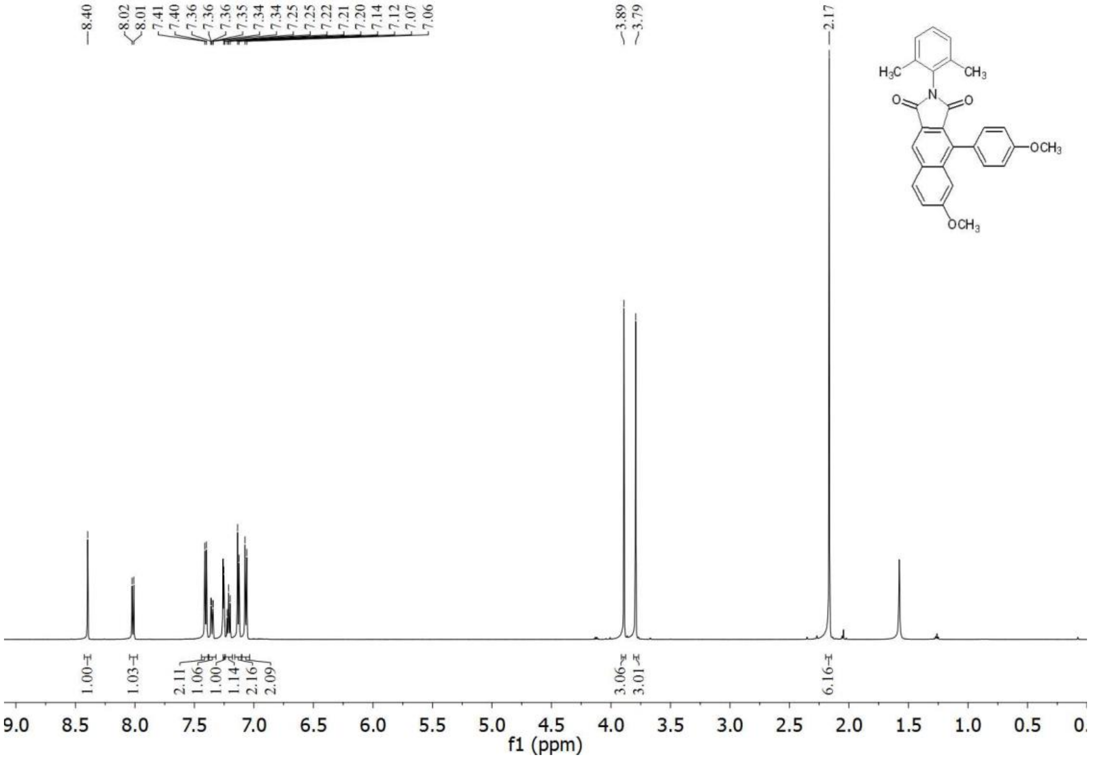

600 MHz ^{1}H NMR spectrum of compound **4s** recorded in $CDCl_3$ at T = 298 K.

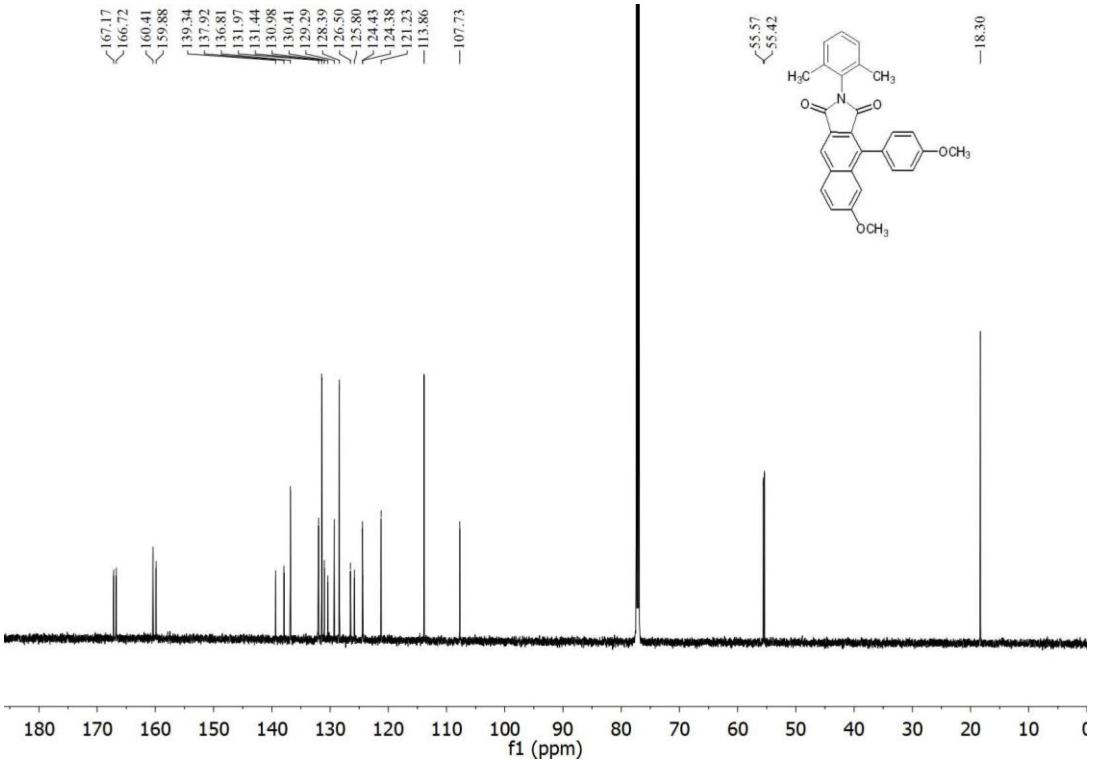

151 MHz ^{13}C NMR spectrum of compound **4s** recorded in $CDCl_3$ at T = 298 K.

4.25. (*E*)-2,9-Diphenyl-3-(phenylimino)-2,3-dihydro-1*H*-benzo[*f*]isoindol-1-one (5)

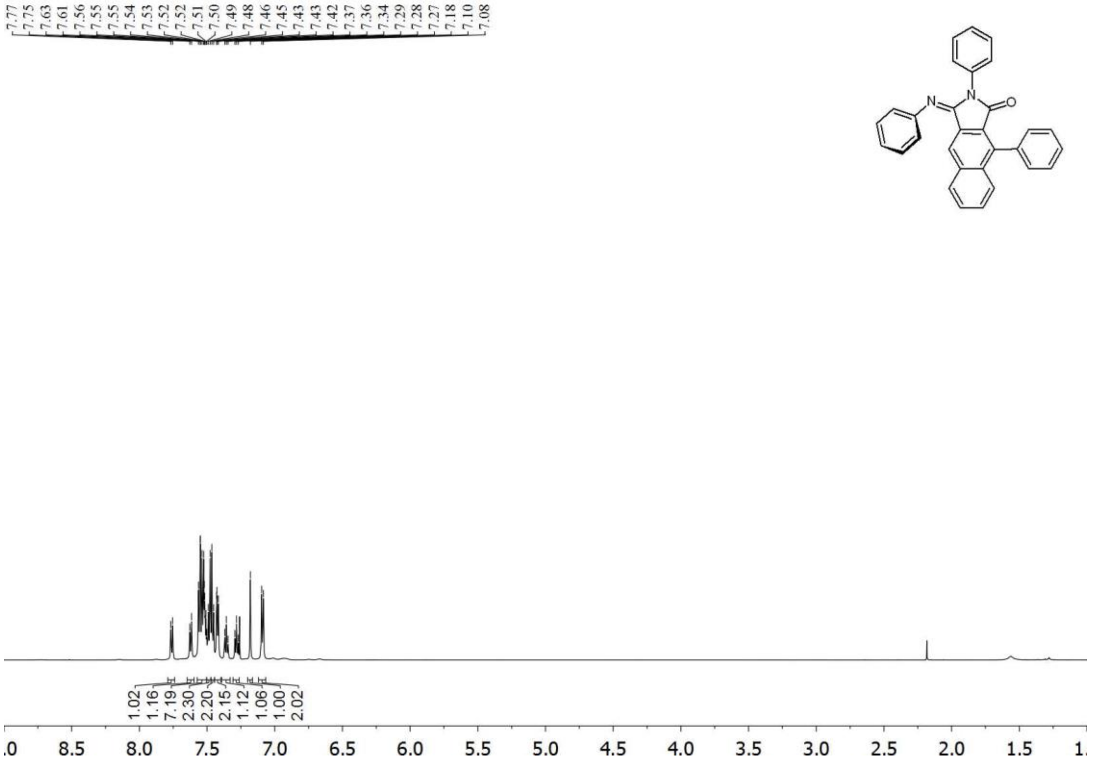

600 MHz ^{1}H NMR spectrum of compound **5** recorded in $CDCl_3$ at T = 298 K.

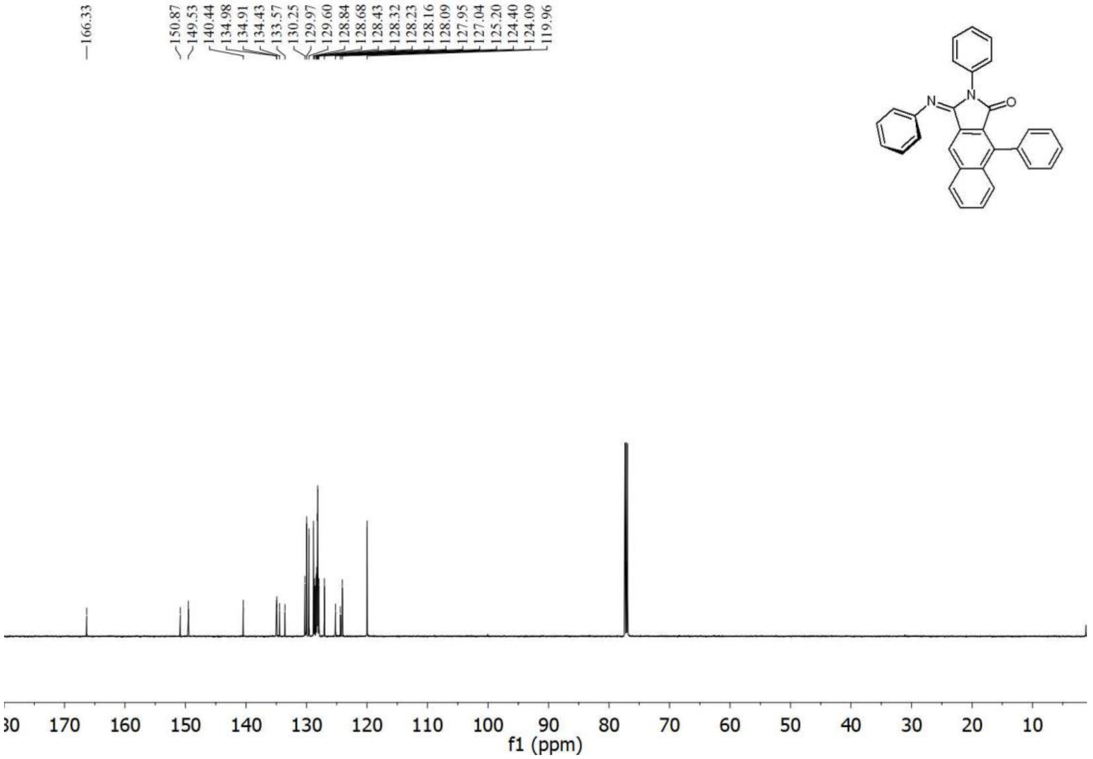

151 MHz ^{13}C NMR spectrum of compound **5** recorded in $CDCl_3$ at T = 298 K.

4.26. 6-Phenyl-12*H*-benzo[*f*]benzo[4,5]-imidazo[2,1-*a*]isoindol-12-one (6)

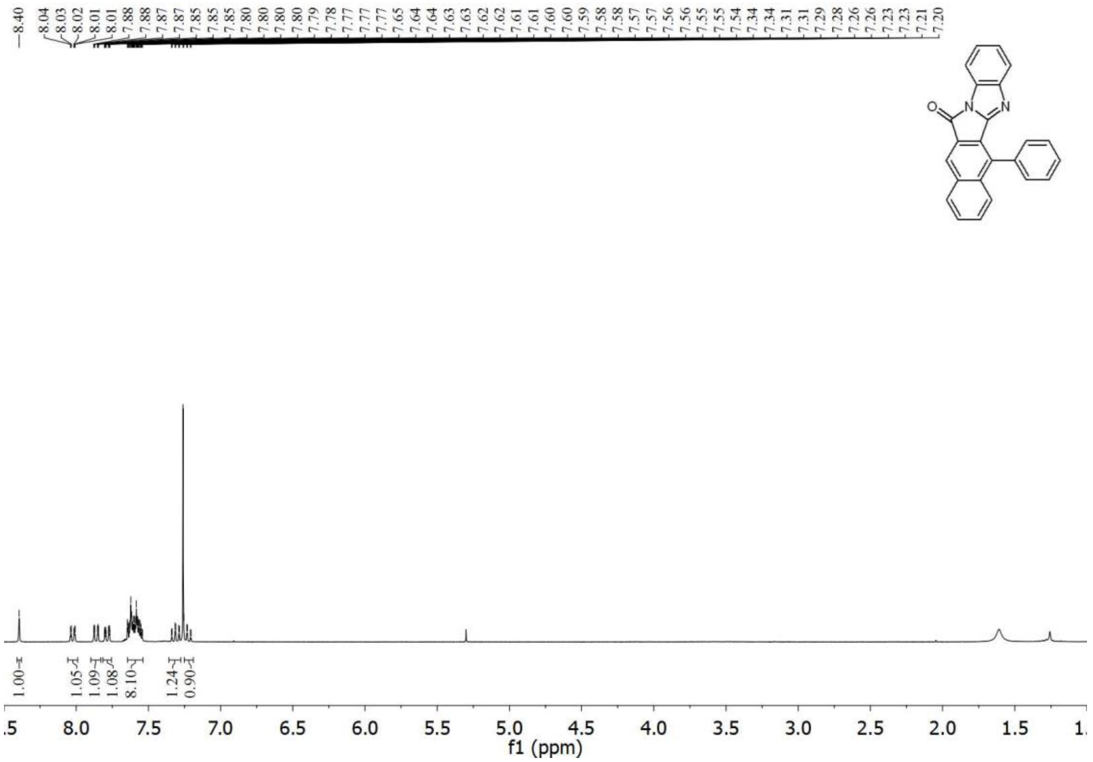

600 MHz ^{1}H NMR spectrum of compound **6** recorded in $CDCl_3$ at T = 298 K.

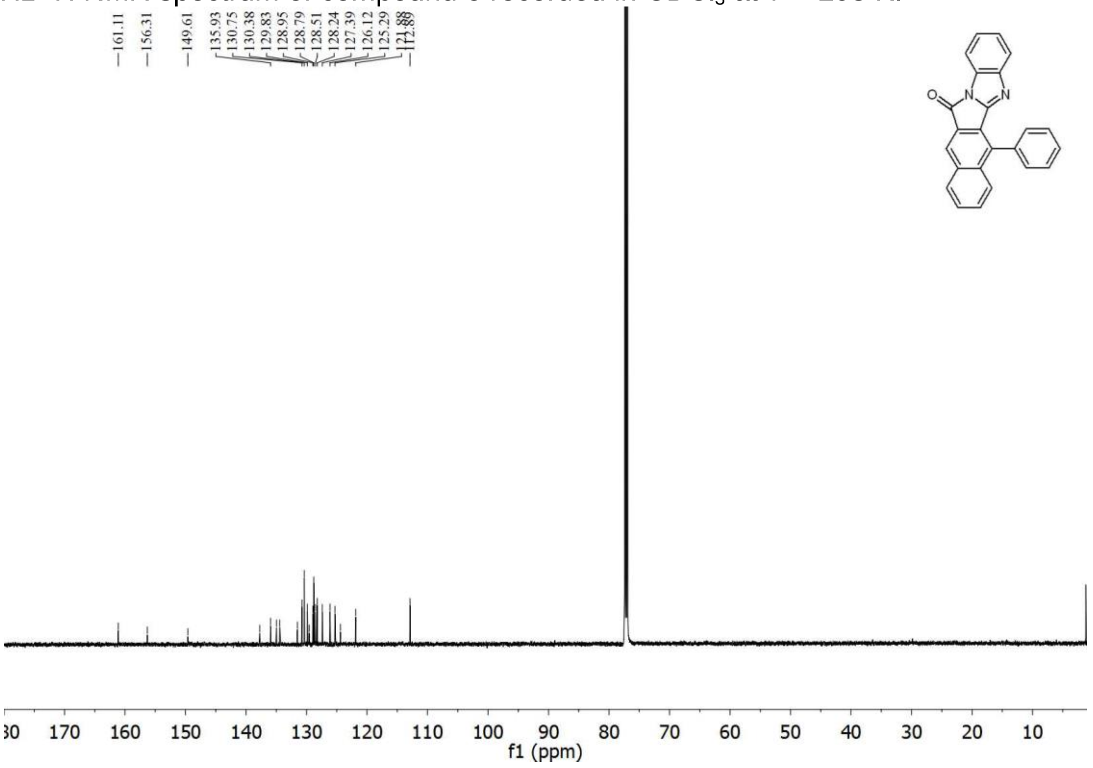

151 MHz ^{13}C NMR spectrum of compound **7b** recorded in $CDCl_3$ at T = 298 K.

5. Crystal Structures of 4b, 5, and 6

All studied crystals were selected directly from a representative sample of the compound. For the data collection the crystal were glued on a thin glas thread. Diffraction data collection for **4b** and **5** was performed using a STOE IPDS-2T two-circle diffractometer.[8] For the data collection of **6** a Bruker four-circle diffractometer equipped with the APEX2 detector[9] was used. Data collection follows in all cases the standard procedures. The same is true for the refinement using the SHELX program system.[10] The DIAMOND[11] software was used for the figures showing the crystal structures presented in this paper.

CCDC 1563985 **4b**, 1563988 **5**, 1564617 **6** contain the supplementary crystallographic data for this paper. The data can be obtained free of charge from The Cambridge Crystallographic Data Centre via www.ccdc.cam.ac.uk/structures.

5.6. Crystal structure of 6-methoxy-4-(4-methoxyphenyl)-2-phenyl-1*H*-benzo[*f*]isoindole-1,3(2*H*)-dione (4b)

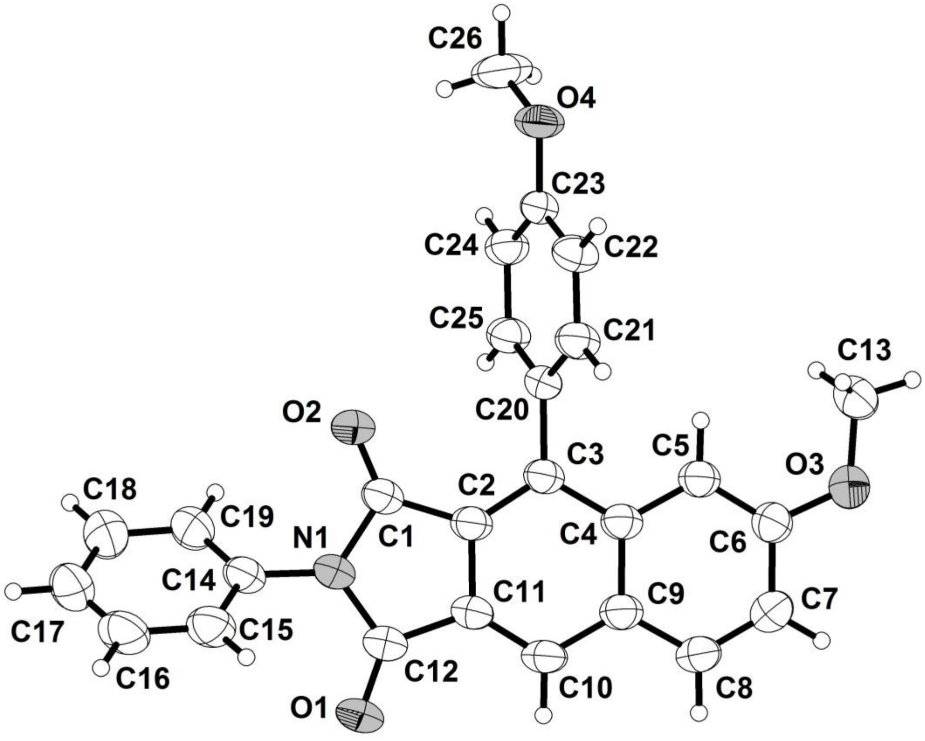

Crystal data and the results of the structure refinement for 4b

Empirical formula	$C_{26}H_{19}NO_4$	
Formula weight	409.42	
Temperature	290 K	
Wavelength	0.71073 Å	
Crystal system	monoclinic	
Space group	$P2_1/n$	
Unit cell dimensions	a = 12.9077(7) Å	
	b = 7.6736(3) Å	β = 101.011(4)°
	c = 21.1316(10) Å	
Volume	2054.52(17) $Å^3$	
Z	4	
Density (calculated)	1.324 g/cm^3	
Absorption coefficient μ	0.09 mm^{-1}	
Crystal size	0.40 x 0.25 x 0.20 mm^3	
Theta range of data collection	2.0 - 29.7°	
Index ranges	$-16 \leq h \leq 16$, $-9 \leq k \leq 9$, $-26 \leq l \leq 26$	
Reflections collected	19278	
Independent reflections	4252 [R_{int} = 0.056]	
Observed reflections	3532	
Completeness	>99%	
Number of refined parameters	282	
Refinement method	Full-matrix least-squares on F^2	
Final R indices [$F^2>2\sigma(F^2)$]	$R1$ = 0.0576, $wR2$ = 0.1085	
Final R indices	$R1$ = 0.0696, $wR2$ = 0.1166	
Largest diff. Peak and hole	0.131 and -0.138 $eÅ^{-3}$	

5.7. Crystal structure of (*E*)-2,9-diphenyl-3-(phenylimino)-2,3-dihydro-1*H*-benzo[*f*]isoindol-1-one (5)

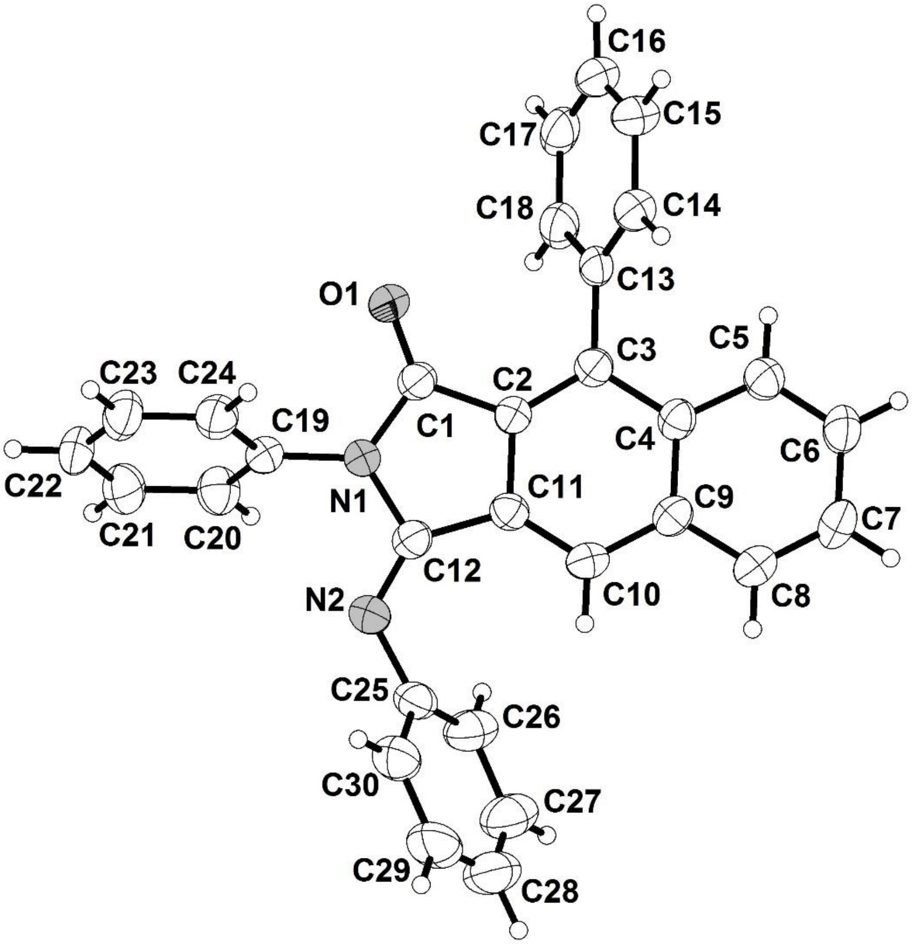

Crystal data and the results of the structure refinement for 5

Empirical formula	$C_{30}H_{20}N_2O$	
Formula weight	424.48	
Temperature	290 K	
Wavelength	0.71073 Å	
Crystal system	triclinic	
Space group	P-1	
Unit cell dimensions	*a* = 9.8395(7) Å	α = 110.995(5)°
	b = 10.05691(7) Å	β = 100.926(5)°
	c = 12.2419(8) Å	γ = 93.129(6)°
Volume	1100.51(14) Å^3	
Z	2	
Density (calculated)	1.281 g/cm^3	

Absorption coefficient μ	0.08 mm^{-1}
Crystal size	0.20 x 0.15 x 0.10 mm^3
Theta range for data collection	1.8 - 27.9°.
Index ranges	$-11 \le h \le 11$, $-11 \le k \le 11$, $-14 \le l \le 14$
Reflections collected	8281
Independent reflections	3824 [R_{int} = 0.063]
Observed reflections	2693
Completeness	>98%
Number of refined parameters	298
Refinement method	Full-matrix least-squares on F^2
Final R indices [$F^2>2\sigma(F^2)$]	$R1$ = 0.0776, $wR2$ = 0.1305
Final R indices	$R1$ = 0.1149*, $wR2$ = 0.1462
Largest diff. Peak and hole	0.14 and -0.16 eÅ^{-3}

* Data collection using a small and weakly diffracting crystal increases this factor systematically, but the factors base on observed reflections and the *wR*2 on all reflections verifies the structural assignement.

5.8. Crystal structure of 6-phenyl-12*H*-benzo[*f*]benzo[4,5]imidazo[2,1-*a*]isoindol-12-one (6)

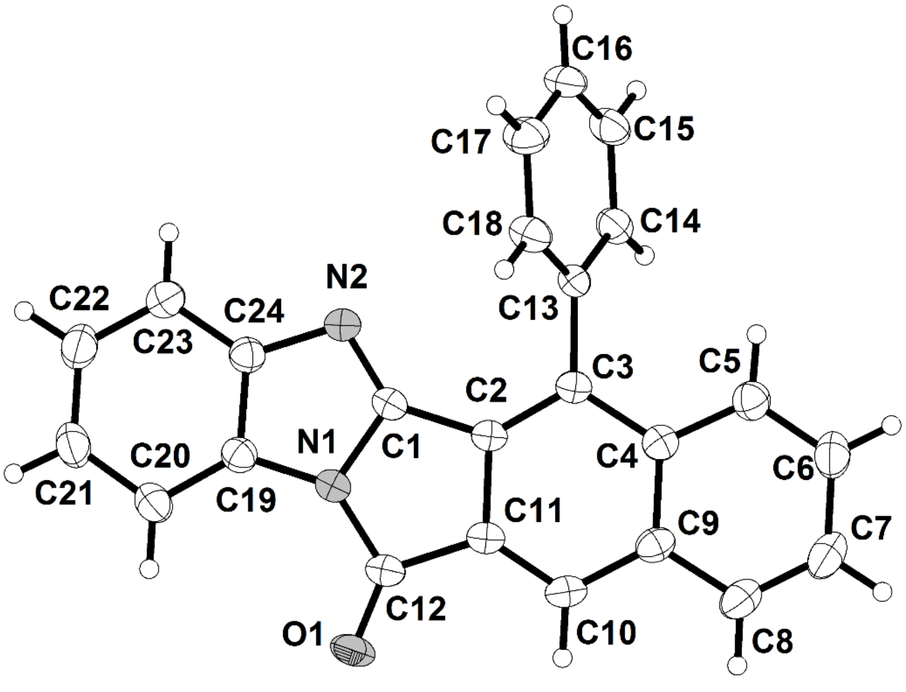

Crystal data and structure refinement for compound 6

Empirical formula	$C_{24}H_{14}N_2O$	
Formula weight	346.37	
Temperature	292 K	
Wavelength	0.71073 Å	
Crystal system	triclinic	
Space group	*P*-1	
Unit cell dimensions	*a* = 9.3052(4) Å	α = 65.898 (2)°
	b = 10.2684(4) Å	β = 64.802(2)°
	c = 10.7616(4) Å	γ = 89.798(2)°
Volume	831.05(6) $Å^3$	
Z	2	
Density (calculated)	1.384 g/cm^3	
Absorption coefficient μ	0.09 mm^{-1}	
Crystal size	0.37 x 0.15 x 0.15 mm^3	
Theta range for data collection	2.2 – 27.5°.	
Index ranges	-12 ≤ *h* ≤ 12, -13 ≤ *k* ≤13, -13 ≤ l ≤13	
Reflections collected	23834	
Independent reflections	3794 [*R*(int) = 0.0275]	
Observed reflections	3235	
Completeness	>99%	
Number of refined parameters	245	
Refinement method	Full-matrix least-squares on F^2	
Final R indices [F^2>2σ (F^2)]	*R*1 = 0.0412, *wR*2 = 0.0855	
Final R indices	*R*1 = 0.0480, *wR*2 = 0.0894	
Largest diff. Peakt and hole	0.25 and -0.21 e$Å^{-3}$	

6. Hammett–Taft correlations of compounds 4a,b,d–f

Table S4. Hammett-Taft parameters[1213]

Substituents	σ_p	σ_R	σ_{p+}	σ_{p-}
MeO	-0.27	-0.43	-0.78	-0.26
H	0	0	0	0
Cl	0.23	-0.16	0.11	0.19
CF_3	0.54	0.09	0.61	0.65
CN	0.66	0.18	0.66	1.00

Table S5. Selected photophysical data ([nm], [cm^{-1}]) of compounds **4a,b,d–f**.

Compound	Substituent	$\lambda_{max,abs}$		$\lambda_{max,em}$		Stokes shift $\Delta\tilde{\nu}$
		[nm]	[cm^{-1}]	[nm]	[cm^{-1}]	[cm^{-1}]
**4b**	MeO	379	26400	444	22500	3900
**4a**	H	364.5	27400	408.5	24500	3000
**4e**	Cl	366	27300	417	24000	3300
**4f**	CF_3	359	27900	397	25200	2700
**4d**	CN	365	27400	401	24900	2500

Table S6. Regression analyses of the σ – photophysical properties correlations of compounds **4a,b,d–f**.

σ parameter	Absorption $\lambda_{max,abs}$ [cm^{-1}]	Emission $\lambda_{max,em}$ [cm^{-1}]	Stokes shift $\Delta\tilde{\nu}$ [cm^{-1}]
σ_p	$\lambda_{max,abs} = 1097 \cdot \sigma_p + 27024$; $R^2 = 0.6$	$\lambda_{max,em} = 2394.2 \cdot \sigma_p + 23666$; $R^2 = 0.7504$	$\Delta\tilde{\nu} = -1297.2 \cdot \sigma_p + 3358$; $R^2 = 0.7859$
**σ_R**	**$\lambda_{max,abs} = 1954.3 \cdot \sigma_R + 27404$; $R^2 = 0.669$**	**$\lambda_{max,em} = 4273.8 \cdot \sigma_R + 24495$; $R^2 = 0.925$**	**$\Delta\tilde{\nu} = -2319.4 \cdot \sigma_R + 2908.6$; $R^2 = 0.989$**
σ_{p+}	$\lambda_{max,abs} = 834.27 \cdot \sigma_{p+} + 27179$; $R^2 = 0.8061$	$\lambda_{max,em} = 1741.1 \cdot \sigma_{p+} + 24013$; $R^2 = 0.9218$	$\Delta\tilde{\nu} = -906.81 \cdot \sigma_{p+} + 3165.9$; $R^2 = 0.8921$
σ_{p-}	$\lambda_{max,abs} = 714.2 \cdot \sigma_{p-} + 27053$; $R^2 = 0.4473$	$\lambda_{max,em} = 1694.7 \cdot \sigma_{p-} + 23686$; $R^2 = 0.6613$	$\Delta\tilde{\nu} = -980.46 \cdot \sigma_{p-} + 3366.9$; $R^2 = 0.7897$

[1] Kofler, L. *Sci. Pharm.* **1966**, 147-166.

[2] Cabré-Castellvi, J.; Palomo-Coll, A.; Palomo-Coll, A. L. *Synthesis* **1981**, 616-620. DOI: 10.1055/s-1981-29544

[3] Baddar, F. G.; El-Assal, L. S.; Gindy, M. *J. Chem. Soc.* **1948**, 1270-1272. DOI: 10.1039/JR9480001270

[4] Katritzky, A. R.; Ozcan, S.; Todadze, E. *Org. Biomol. Chem.* **2010**, *8*, 1296-1300. DOI: 10.1039/C000684J

[5] Kim, K. H.; Lim, C. H.; Lim, J. W.; Kim, J. N. *Adv. Synth. Catal.* **2014**, *356*, 697-704. DOI: 10.1002/adsc.201301169

[6] Islam, A. M.; Elsherief, A. M. S.; Hamzah, M. A. *Indian J. Chem. Sect. B* **1978**, *16*, 686-688.

[7] Islam, A. M.; Khalil, A. M.; Elmaghra, A. A. *Egypt. J. Chem.* **1974**, 17, 749-757.

[8] STOE & Cie, X-Area data collection and reduction software, Darmstadt, 2009.

[9] Bruker, *SAINT, APEX2*. Bruker AXS Inc., Madison, Wisconsin, USA, 2012.

[10] Sheldrick. G. M. *Acta Crystallogr.* **2015**, *C71*, 3-8. doi: 10.1107/S2053229614024218

[11] Brandenburg, K.: *DIAMOND*. Visual Crystal Structure Information System. Version 3.2i. Crystal Impact, Bonn, Germany 2012.

[12] Hansch, C.; Leo, A.; Taft, R. W. *Chem. Rev.* **1991**, *91*, 165-195. DOI: 10.1021/cr00002a004
